# Stereo-cell deciphers the spatial and functional heterogeneity of polyploid hepatocytes

**DOI:** 10.1093/gigascience/giag023

**Published:** 2026-03-02

**Authors:** Yongqing Yang, Jiahui Luo, Yier Cai, Pengcheng Guo, Qiang Guo, Hong Wu, Longqi Liu, Shijie Hao

**Affiliations:** College of Life Sciences, University of Chinese Academy of Sciences, 19A Yuquan Road, Shijingshan District, Beijing 100049, China; BGI Research , 203 Zhenzhong Road, Xihu District, Hangzhou 310030, China; BGI Research, 203 Zhenzhong Road, Xihu District, Hangzhou 310030, China; School of Biology and Biological Engineering, South China University of Technology, 382 Waihuan East Road, Panyu District, Guangzhou 510006, China; Guangdong Country Garden School, 101 Country Garden Road, Shunde District, Foshan 528311, China; BGI Research , 203 Zhenzhong Road, Xihu District, Hangzhou 310030, China; School of Biology and Biological Engineering, South China University of Technology, 382 Waihuan East Road, Panyu District, Guangzhou 510006, China; State Key Laboratory of Genome and Multi-omics Technologies, BGI Research, 203 Zhenzhong Road, Xihu District, Hangzhou 310030, China; BGI Research , 203 Zhenzhong Road, Xihu District, Hangzhou 310030, China; College of Life Sciences, University of Chinese Academy of Sciences, 19A Yuquan Road, Shijingshan District, Beijing 100049, China; BGI Research , 203 Zhenzhong Road, Xihu District, Hangzhou 310030, China; BGI Research , 203 Zhenzhong Road, Xihu District, Hangzhou 310030, China; State Key Laboratory of Genome and Multi-omics Technologies, BGI Research, 203 Zhenzhong Road, Xihu District, Hangzhou 310030, China; BGI Research , 203 Zhenzhong Road, Xihu District, Hangzhou 310030, China; School of Biology and Biological Engineering, South China University of Technology, 382 Waihuan East Road, Panyu District, Guangzhou 510006, China; State Key Laboratory of Genome and Multi-omics Technologies, BGI Research, 203 Zhenzhong Road, Xihu District, Hangzhou 310030, China

**Keywords:** Hepatocyte ploidy, imaging-based ploidy identification, spatially resolved single-cell sequencing, hepatocyte metabolism

## Abstract

A characteristic feature of the liver is the presence of numerous polyploid hepatocytes. However, the functional distinctions among diploid, tetraploid, and octoploid hepatocytes remain poorly understood. In this study, we employed the spatially resolved single-cell sequencing technology, Stereo-cell, to dissect the transcriptomic and functional heterogeneity across hepatocyte ploidy subtypes. We detail the development of Stereo-cell imaging-based ploidy identification (SCIPI), a technical pipeline that integrates bright-field cell contour recognition, DAPI-based nuclear area and number quantification, and UMI-barcoded single-cell transcriptomics. This approach enables precise identification of 6 core hepatocyte subtypes: mononucleated diploid (2n×1), mononucleated tetraploid (4n×1), binucleated tetraploid (2n×2), mononucleated octoploid (8n×1), binucleated octoploid (4n×2), and binucleated hexadecaploid (8n×2) hepatocytes. Single-cell transcriptomic analysis based on ploidy annotation revealed that gene expression levels positively with increasing ploidy and nuclear number. Metabolic pathway-associated genes were significantly upregulated in polyploid cells, suggesting that cellular polyploidization enhances the metabolic activity of hepatocytes. Furthermore, this SCIPI strategy is broadly applicable to the study of various polyploid tissues, offering a novel and versatile framework for innovative ploidy-resolved research across diverse biological researches.

## Background

Polyploidy, a state characterized by cells containing more than a diploid DNA content, represents a physiological function and a biological phenomenon of particular significance in the mammalian liver [1–5]. The liver has the ability to dynamically adjust its ploidy constituent throughout development and in response to diverse stresses, employing polyploidization as a fundamental mechanism to govern growth regulation and functional adaptation [6, 7]. Tetraploid hepatocytes are predominantly found in the liver and arise as a result of a developmentally programmed cytokinesis failure during postnatal maturation [6, 8, 9]. The formation of tetraploid hepatocytes begins during the weaning transition, where a diploid hepatocyte either completes normal cytokinesis to generate 2 diploid cells or undergoes incomplete division to form a binucleated tetraploid (2n×2) [9–11]. Hepatocytes of even higher ploidy are generated from binucleated tetraploids via aberrant cell cycles [12, 13]. Polyploidization is critically regulated by the PI3K-AKT pathway [14], MAPK pathway [15], and E2F transcription factors [16–18], and knockdown of E2F8 demonstrates a decrease in polyploidy in the liver. Previous studies have suggested that polyploidy serves as a genomic buffer to enhance organ resilience under physiological conditions [19], but accelerates liver dysfunction in the pathological process [20–22]. Upon hepatocyte-specific knockout of the master regulators HNF4α or CEBPA, polyploid hepatocytes buffer the genetic lesion by non-randomly selecting the wild-type allele, thereby compensating for the haploinsufficiency and alleviating senescence in the liver through the enrichment of completely wild-type nuclei [23]. Premature weaning or knockdown of E2F8 to alter liver ploidy revealed that polyploidy acts as a tumor suppressor to prevent oncogenic progression in the liver [24]. In contrast, in nonalcoholic fatty liver disease, oxidative stress activates the ATR/p53/p21 DNA damage checkpoint, promoting endoreplication that generates highly polyploid mononuclear cells which facilitate hepatocellular carcinoma (HCC) development [22]. Hepatocyte-specific RAD51 [25] or PRMT5 [26] knockout also induces G2/M arrest and promotes the accumulation of highly polyploid mononucleated cells, ultimately leading to progression to fibrosis and HCC. These findings imply polyploidization as a double-edged sword, physiologically beneficial yet pathologically malignant when dysregulated. However, the precise hepatocyte ploidy subtypes and underlying functional heterogeneity remain largely unexplored.

The state of hepatocyte ploidy is accompanied by a concomitant increase in nuclear size [15]. Two primary approaches have been used to study hepatocyte polyploidy, each with inherent limitations. The first method involves co-staining liver cryosections with nucleic acid and plasma membrane markers by identifying ploidy through measurement of maximum nuclear diameter or area *in situ* [15, 27]. While this technique classifies all ploidy subtypes, it does not connect the ploidy state with transcriptomic information. The second method is fluorescence-activated cell sorting (FACS), which is employed to isolate cells or nuclei based on nucleic acid fluorescence intensity [28, 29]. The sorted diploid, tetraploid, and higher-ploidy populations obtained through this method can subsequently be analyzed by single-cell RNA-seq (scRNA-seq) or single-nucleus RNA-seq (snRNA-seq). Although polyploidy can be characterized with FACS at the single-cell level, it must be noted that this method fundamentally loses the critical biological distinction between mononucleated and binucleated polyploid subtypes.

To address these limitations, we developed Stereo-cell [30–35] imaging-based ploidy identification (SCIPI), a comprehensive pipeline which uniquely integrates spatial transcriptomics with imaging to enable simultaneous transcriptome profiling and ploidy determination within the same cell [36]. The strategy precisely classifies 6 key hepatocyte ploidy subtypes: mononucleated diploid (2n×1), mononucleated tetraploid (4n×1), binucleated tetraploid (2n×2), mononucleated octoploid (8n×1), binucleated octoploid (4n×2), and binucleated hexadecaploid (8n×2) hepatocytes. We performed single-cell transcriptomic analysis of these polyploid hepatocytes, constructing transcriptomic profiles of ploidy subtypes and providing a comprehensive resource for gene expression patterns in hepatocyte polyploidy.

## Results

### Integrated Stereo-cell matrix of gene expression and ploidy

Polyploidization is critical for liver development, functional maturation, and disease pathogenesis [12]. To investigate the functional implications of under characterized hepatocyte ploidy subtypes, we isolated single hepatocytes from C57BL/6 mice at 8 weeks of age using an optimized two-step collagenase perfusion protocol [37–43] to obtain highly viable cells. Due to the sensitivity of primary hepatocytes to handling-induced death, Stereo-cell [33] sequencing was performed immediately to ensure viability. We conducted 2 biologically independent replicates (hep56_H8 and hep56_M1) in total. Given the large size of hepatocytes, we loaded a total of 7,721 cells onto 2 Stereo chips to achieve an optimal cell density on the chip surface. After quality control, 4,888 cells were obtained, resulting in an overall cell capture rate of 68.5%. High reproducibility was observed between the 2 biological replicate chips (Supplementary Fig. S1a).

**Figure 1:**
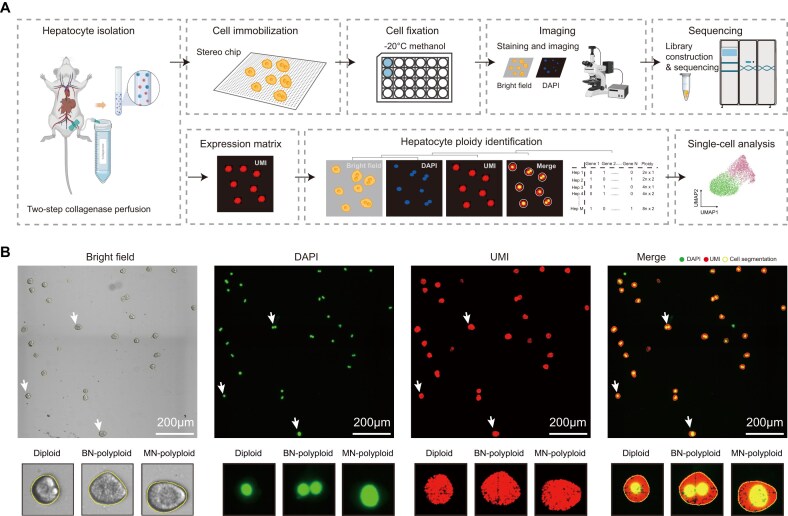
Comprehensive Stereo-cell experimental workflow for hepatocytes. (a) Schematic of the Stereo-cell experimental workflow for hepatocytes. (b) Representative images show hepatocytes across multiple fields of view on the Stereo chip. Arrowheads indicate selected cells. BN-polyploid (binucleated polyploid hepatocyte); MN-polyploid (mononucleated polyploid hepatocyte). Scale bar: 200 µm.

To obtain high-confidence identification of polyploid hepatocytes, we established the SCIPI multimodal analytical framework by integrating spatially resolved transcriptional profiles with the concurrent bright-field images and DAPI-based nuclear quantification (Fig. 1a). This tripartite data architecture enables the systematic segmentation of individual hepatocytes, followed by automated ploidy classification through nuclear area quantification that distinguishes mononucleated and binucleated subtypes (Fig. 1b). Identity-matched matrices subsequently align these ploidy annotations with their corresponding transcriptomes while preserving spatial coordinates, permitting comprehensive ploidy-resolved single-cell analysis. The whole experimental workflow from hepatocyte isolation to sequencing typically requires 4 days. SCIPI integrates the multimodal data (imaging and transcriptomics) described above, requiring ∼1 day from raw data to final ploidy annotation.

### Ploidy identification workflow and accuracy assessment

**Figure 2:**
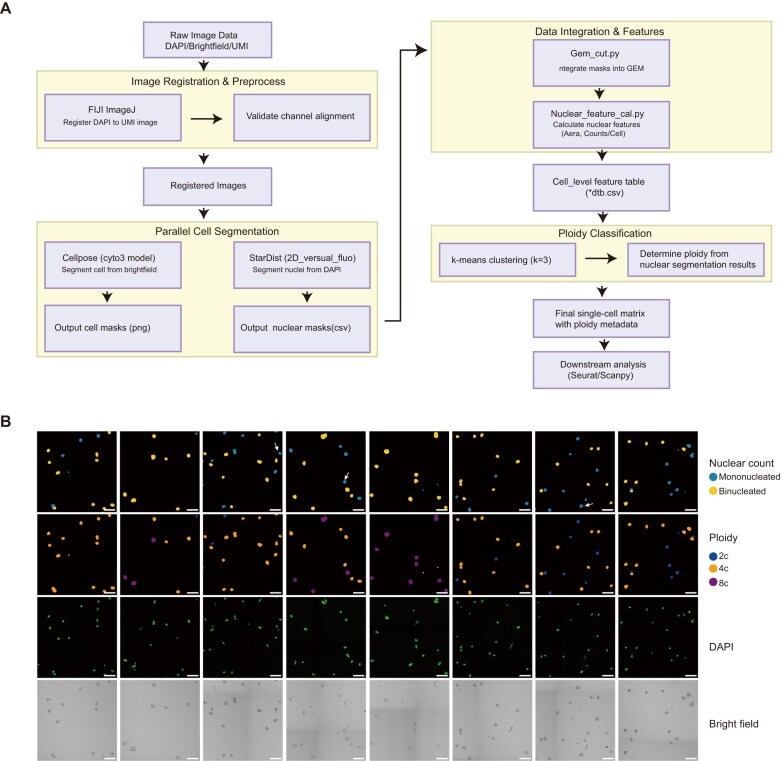
SCIPl pipeline for spatially resolved hepatocyte ploidy profiling. (a) Workflow for identifying hepatocyte ploidy subtypes. (b) Representative field showing identification of hepatocyte ploidy subtypes. Arrows indicate misidentified cells. Scale bar: 100 µm.

The Stereo-cell raw sequencing data was processed with SAW [44] to obtain GEM-format files that have undergone sequence demultiplexing and alignment. This file contains Gene IDs, gene coordinates (x, y), molecular identifier count (MID Count), and exon counts. Based on this information, we can generate in situ unique molecular identifier (UMI) counts image (UMI image) with script gem2mask.py. (Fig. 1a, UMI). Then, perform cell segmentation on the brightfield and DAPI images after registration to the UMI image. According to DAPI masks, cell masks, DAPI area and nuclear count per cell to classify hepatocyte ploidy subtypes (Fig. 2a). To classify hepatocytes into diploid, tetraploid, and octoploid subtypes, we applied k-means clustering to all nuclei segmented by StarDist. This algorithm groups nuclei into clusters by maximizing between-cluster differences in nuclear area, a feature that correlates with the stepwise increase in DNA content from diploid to polyploid states. Based on this nuclear classification, each nucleus was assigned a corresponding ploidy label. Subsequently, considering the number of nuclei contained within each cell (mononucleated or binucleated), the ploidy subtype of each individual cell was comprehensively determined.

The accuracy of ploidy identification is primarily monitored through the following key steps. First, ensure perfect registration between DAPI image and UMI image across multiple regions, with UMIs remaining untransformed. Switch between brightfield (green channel) and UMI (red channel) to confirm that cell boundaries in brightfield images align with UMI image in multiple selected regions. Second, assess cell size parameters during cell segmentation with Cellpose [45]. Manually verify that the majority of hepatocyte boundaries are accurately identified post-segmentation, and adjust size settings if necessary (Cellpose’s automatic size estimation is generally reliable). Finally, cross-validate results using original DAPI, brightfield, and nuclear feature maps. Since the majority of cell nuclei are either single or double, and although a few reports have mentioned cells with 3 nuclei, for the sake of universality and accuracy, only cells with 1 or 2 nuclei are retained here for accuracy testing. Ensure the misidentification error within multiple randomly selected region is below 3 cells per region (Fig. 2b).

### SCIPI for quantifying polyploid hepatocyte subpopulation

**Figure 3:**
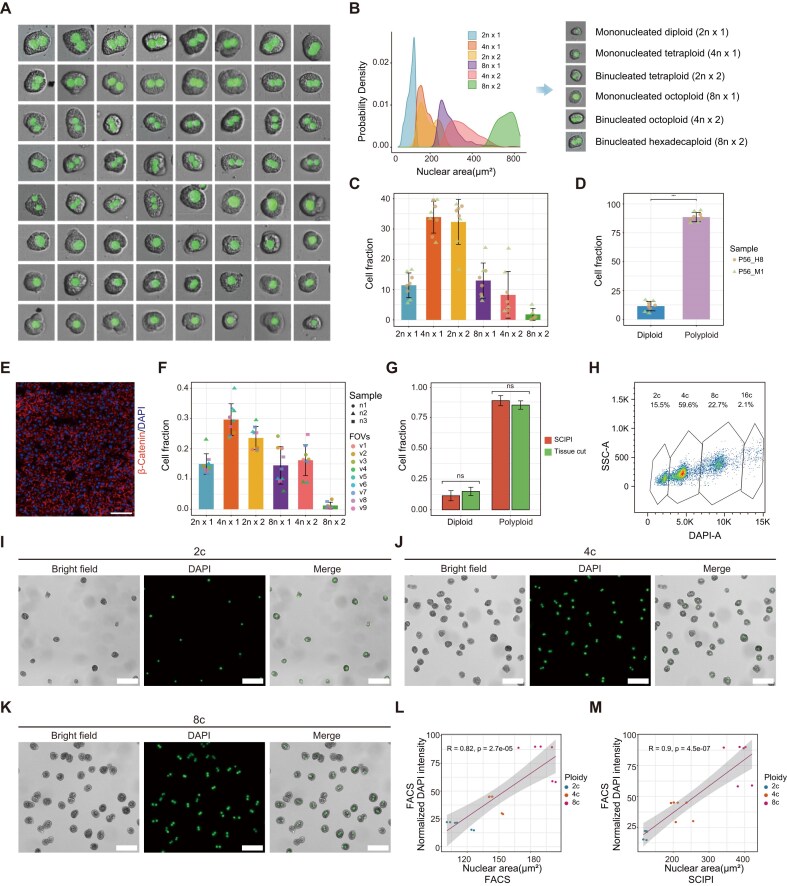
Quantifying and classifying hepatocyte polyploidy. (a) Representative hepatocytes with varying nuclear sizes, showing binucleated and mononucleated types. (b) Density curves for nuclear area distribution of hepatocyte ploidy subpopulations (2n×1: diploid; 4n×1: mononucleate tetraploid; 2n×2: binucleate tetraploid; 8n×1: mononucleate octoploid; 4n×2: binucleate octoploid; and 8n×2: binucleate hexadecaploid). (c) Proportions of the hepatocyte ploidy subpopulations as identified by SCIPI. (d) Proportion of diploid and polyploid hepatocytes in SCIPI. Two-sided unpaired Student’s *t*-test; *P < 0.05, **P < 0.01, ***P < 0.001; ns, no significance. (e) Immunofluorescence of β-Catenin and DAPI in P56 C57BL/6 mouse liver sections (*n* = 3 biologically independent animals). Scale bar: 100 µm. (f) Hepatocyte ploidy subpopulation fractions in P56 C57BL/6 mouse liver sections. Data are from 3 mice (n1–n3), with 3 random fields of view (v1–v9) per mouse. (g) Hepatocyte ploidy fractions (diploid vs. polyploid) from SCIPI and liver section analysis. Two-sided unpaired Student’s t-test; ns, no significance. (h) Flow cytometric DNA content analysis (DAPI staining) of hepatocytes, showing diploid (2c, 15.5%), tetraploid (4c, 59.6%), octoploid (8c, 22.7%), and hexadecaploid (16c, 2.1%) cells. (i–k) Brightfield, DAPI, and merged images of flow-sorted diploid (2c) in (i), tetraploid (4c) in (j), and octoploid cells in (k). Scale bar, 100 µm. (l) Correlation between nuclear area and normalized DAPI fluorescence intensity in flow-sorted diploid, tetraploid, and octoploid hepatocytes (*n* = 3 P56 C57BL/6 mice; *R* = 0.84, *P* = 2.7e-05). (m) Correlation between nuclear area (measured by SCIPI) and normalized DAPI fluorescence intensity (assessed by FACS) in flow-sorted 2c, 4c, and 8c hepatocytes (*n* = 3 P56 C57BL/6 mice; Pearson correlation *R* = 0.9, *P* = 4.5e-07).

Polyploidization constitutes an essential biological feature of mammalian hepatocytes, with developmental programming driving nuclear DNA accumulation through both cytokinesis failure and nuclear duplication [8]. In our staining image, the nuclear area of polyploid hepatocytes was significantly larger than that of diploid nuclei (Fig. 3a), consistent with previously reported studies showing a linear correlation between nuclear area and DNA content [15]. Based on nuclear area distribution, we classified hepatocytes into 6 ploidy classes (Fig. 3b): 2n×1: diploid; 4n×1: mononucleate tetraploid; 2n×2: binucleate tetraploid; 8n×1: mononucleate octoploid; 4n×2: binucleate octoploid; and 8n×2: binucleate hexadecaploid. Distinct nuclear area distribution patterns were observed across diploid (2c), tetraploid (4c), octoploid (8c), and hexadecaploid (16c) cells levels, whereas similar nuclear area distribution profiles were exhibited by different nuclear types within the same ploidy level.

Quantitative analysis revealed that polyploid hepatocytes accounted for over 85% of total hepatocytes in P56 mice (Fig. 3c–d). The ploidy distribution was as follows: 11.4% diploid, 66.2% tetraploid (33.9% mononucleated and 32.3% binucleated), 21.2% octoploid (13.0% mononucleated and 8.2% binucleated), and 1.8% hexadecaploid. This distribution is consistent with previously reported proportions in adult mouse liver [10, 46–48]. To further validate the robustness of the SCIPI method for ploidy identification, we performed immunofluorescence staining on liver tissue sections and applied the same ploidy recognition strategy. This analysis yielded a similar overall ploidy distribution profile, and no significant difference was observed in the identification of polyploid vs. diploid cells between the SCIPI-based and tissue-section-based methods (Fig. 3f–g).

To confirm that the Stereo-cell platform underlying SCIPI technology captures all hepatocyte ploidy types without bias, we performed FACS to isolate diploid, tetraploid, octoploid, and hexadecaploid hepatocytes. The resulting ploidy proportions were highly consistent with those obtained by SCIPI (Fig. 3h, Supplementary Fig. S1b–f). Furthermore, FACS-sorted diploid, tetraploid, and octoploid hepatocytes were plated on slides for microscopic imaging to obtain nuclear size measurements (Fig. 3i–k). Statistical analysis of the DAPI fluorescence intensity vs. the corresponding nuclear area for these FACS-sorted cells showed a significant positive correlation (*R* = 0.82, *P* = 2.7e-05, Fig. 3l). Finally, we assessed the consistency between the fluorescence intensity of FACS-sorted cells of different ploidies and the nuclear area measured by SCIPI for corresponding ploidy classes. A high correlation was also observed here (*R* = 0.9, *P* = 5.1e-07, Fig. 3m). In summary, these results demonstrate the accuracy and reliability of the SCIPI method for classifying hepatocyte ploidy.

### Polyploid hepatocyte distribution across liver lobule

**Figure 4:**
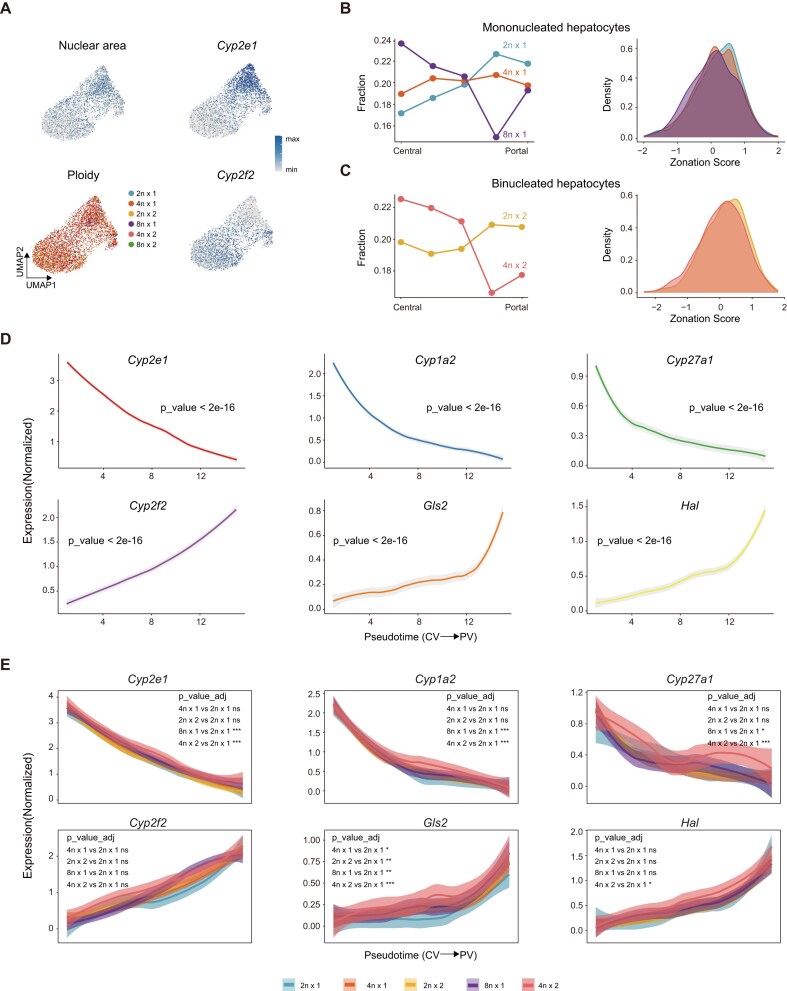
Gene expression along CV to PV trajectory by ploidy. (a) UMAP plots show hepatocyte nuclear area, ploidy subpopulation distribution, and expression of zonation genes *Cyp2e1* and *Cyp2f2*. (b) Zonal distribution of mononucleated hepatocytes (2n×1; 4n×1; and 8n×1) from the central vein (CV) to the portal vein (PV), and the corresponding density curves. (c) Zonal distribution of binucleated hepatocytes (2n×2; 4n×2) from the CV to the PV, and the corresponding density curves. (d) Zonation gene expression from the CV to the PV. ANOVA *F*-test; *P < 0.05, **P < 0.01, ***P < 0.001; ns, no significance. (e) Expression profiles of zonation genes in hepatocyte ploidy subpopulations along the CV–PV axis. Wilcoxon rank-sum test with Benjamini–Hochberg (BH) correction; *P < 0.05, **P < 0.01, ***P < 0.001; ns, no significance.

Following ploidy identification, we obtained high-quality single-cell data annotated with ploidy information. These data were integrated with a published single-cell transcriptomic dataset of homeostasis adult mouse hepatocytes [32], demonstrating successful integration (Supplementary Fig. S2a, c–h). Regardless of integration, hepatocytes of different ploidy subtypes showed an irregularly intermixed distribution in uniform manifold approximation and projection (UMAP) (Fig. 4a, Supplementary Fig. S2b).

Based on the expression of liver zonation marker genes (e.g. pericentral marker *Cyp2e1* and periportal marker *Cyp2f2*), we categorized hepatocytes into pericentral (PC) and periportal (PP) lobule zone subpopulations (Fig. 4a). Weighted gene signature scoring for PC- and PP-specific genes allowed us to accurately mapped the spatial distribution of different hepatocyte ploidy subtypes across the hepatic lobule (Fig. 4b and c). Due to low cell counts and high statistical variance, hexadecaploid hepatocytes were excluded from this analysis. The lobule distribution patterns of SCIPI-identified hepatocyte ploidy subtypes aligned with results from previous studies based on nuclear and membrane staining of liver tissue sections [15]. SCIPI analysis revealed significant spatial distribution features: 4n×2 and 8n×1 hepatocytes were enriched in the pericentral region, whereas 2n×1 and 2n×2 hepatocytes were predominantly located in the periportal region, suggesting a preferential enrichment of higher-ploidy hepatocytes in the pericentral zone.

To investigate the expression dynamics of zonation-related genes across different hepatocyte ploidy states, we analyzed the expression patterns of CV-enriched marker genes (*Cyp2e1, Cyp1a2*, and *Cyp27a1*) and PV-enriched marker genes (*Cyp2f2, Gls2*, and *Hal*) along the hepatic lobular axis (Fig. 4d). The expression gradients of these genes along the CV-to-PV axis were consistent with the well-characterized zonation patterns reported previously [28, 32, 49, 50]. Subsequently, we stratified the analysis by 5 hepatocyte ploidy subtypes (2n×1, 4n×1, 2n×2, 8n×1, and 4n×2) (Fig. 4e). Most genes retained a conserved zonated expression trend across these subtypes. However, their expression levels along the CV–PV axis showed significant differences correlated with increasing ploidy.

### The transcriptomic profiles analysis of polyploidy hepatocyte

**Figure 5:**
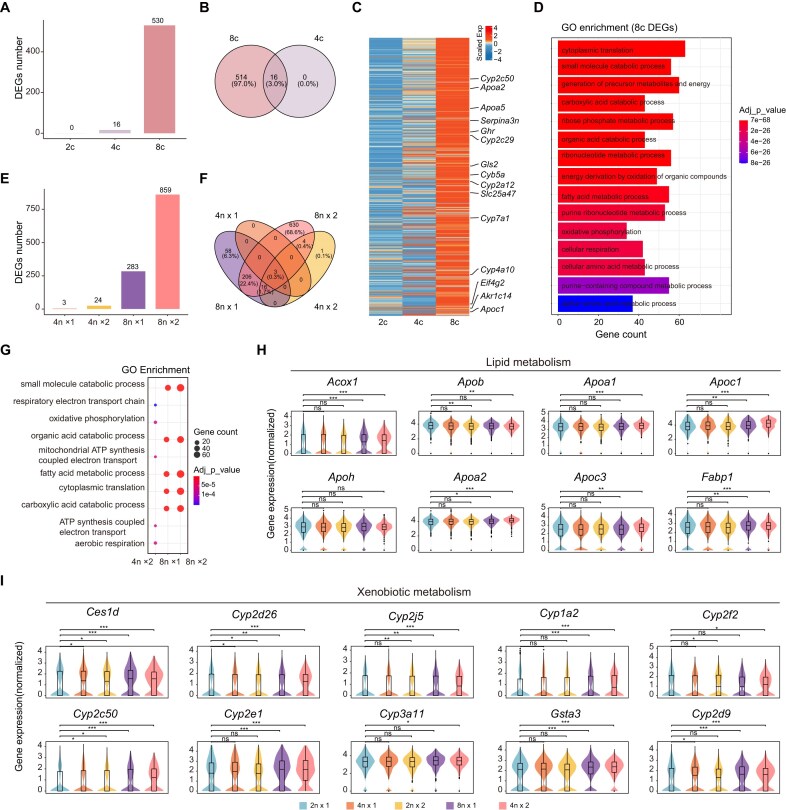
Transcriptomic diversity across hepatocyte ploidy subpopulations. (a) Number of upregulated DEGs in diploid (2c), tetraploid (4c), and octoploid (8c) cells, identified by adjusted *P*-value (adj_p_val) < 0.05 and average log2 fold change (avg_log2FC) >0. (b) Venn diagram depicting the overlap of upregulated DEGs between 8c and 4c cells. (c) Heatmap illustrating the scaled expression profiles of upregulated DEGs across 2c, 4c, and 8c cells, with representative genes labeled on the right. (d) Bar plot of gene ontology (GO) biological process enrichment analysis for upregulated DEGs in 8c cells. (e) Number of upregulated DEGs in mononuclear tetraploid (4n×1), binuclear tetraploid (2n×2), mononuclear octoploid (8n×1), and binuclear octoploid (4n×2) cells. Adj_p_val < 0.05 and avg_log2FC >0. (f) Venn diagram showing the overlap of upregulated DEGs among 4n×1, 2n×2, 8n×1, and 4n×2 cells. (g) Dot plot of integrated GO enrichment analysis, displaying the top functional pathways of upregulated DEGs in 2n×2, 8n×1, and 4n×2 cells. (h–i) Violin plots showing the normalized expression levels of representative lipid metabolism-related and xenobiotic metabolism-related genes across 2n×1, 4n×1, 2n×2, 8n×1, and 4n×2 cells. Kruskal–Wallis overall test followed by Wilcoxon pairwise tests with BH correction (ns, not significant; *P < 0.05; **P < 0.01; ***P < 0.001).

To investigate whether gene expression is correspondingly enhanced with increasing genomic ploidy, we performed comparative transcriptomic profiling of diploid (2c), tetraploid (4c), and octoploid (8c) hepatocytes. Differentially expressed genes (DEGs) were identified by comparing tetraploid vs. diploid, octoploid vs. diploid, and diploid vs. polyploid populations, with significance defined as an adjusted *P*-value <0.05. We observed that tetraploid cells exhibited fewer upregulated DEGs compared with octoploid cells (Fig. 5a), and the upregulated gene set in octoploid hepatocytes encompassed those upregulated in tetraploid cells (Fig. 5b). No upregulated DEGs were detected in diploid cells relative to polyploid hepatocytes. Heatmap analysis revealed a gradual increase in expression of these octoploid-associated genes from 2c to 8c (Fig. 5c). GO enrichment analysis of the DEGs highlighted terms related to cytoplasmic translation, small molecule catabolism, generation of precursor metabolites and energy, reflecting enhanced metabolic and biosynthetic activity in octoploid hepatocytes (Fig. 5d).

Leveraging the advantage of SCIPI, we further explored nuclear count-associated transcriptional changes independent of total DNA content. We found that binucleated hepatocytes displayed more upregulated DEGs than mononucleated cells, both within tetraploid and octoploid populations (Fig. 5e). Notably, the upregulated DEGs in both mononucleated and binucleated tetraploid hepatocytes were largely contained within the octoploid DEG set (Fig. 5f). GO analysis revealed that tetraploid and octoploid hepatocytes shared enriched pathways such as oxidative phosphorylation, ATP synthesis coupled electron transport, and aerobic respiration, while octoploid cells additionally showed enrichment in small molecule metabolism, organic acid metabolism, and fatty acid metabolism (Fig. 5g, Supplementary Fig. S3a–c). Representative lipid metabolism genes (e.g. *Acox1, Apoa1*) and xenobiotic metabolism genes (e.g. *Ces1d, Cyp2e1*) confirmed that their expression levels were progressively elevated with increasing ploidy and nuclear number (Fig. 5h–i). Hepatic transcription factors (e.g. *Hnf4a, Cebpa*) displayed a similar trend, with their expression increasing progressively with higher ploidy (Supplementary Fig. S3d).

## Discussion

We developed the SCIPI method, which reliably classifies hepatocyte ploidy subtypes. In P56 mice, the identified proportions were as follows: 2n×1 (11.4%), 4n×1 (33.9%), 2n×2 (32.3%), 8n×1 (13.0%), 4n×2 (8.1%), and 8n×2 (1.8%). These results showed high concordance with ploidy distributions determined by nuclear area measurement in liver tissue sections and by FACS. During the imaging of FACS-sorted cells, we found the contamination of the sorted diploid cell population with ∼13% binucleated cells (supplementary Fig. S1g). This observation underscores the necessity for stringent gating in future FACS-based studies investigating transcriptional differences across ploidy types.

Our study provides a comprehensive map of transcriptional heterogeneity in polyploid hepatocytes. The proportion of 8n×1 and 4n×2 hepatocytes progressively decreased along the CV to PV axis, whereas the proportions of 2n×1 and 2n×2 hepatocytes gradually increased. Transcriptomic analysis revealed that octoploid hepatocytes upregulated a greater number of DEGs compared to tetraploid cells, relative to diploid counterparts. Comparative analysis between cellular ploidy and nuclear number indicated that binucleated polyploid hepatocytes exhibited more upregulated DEGs than their mononucleated counterparts. The upregulated DEGs in polyploid cells were significantly enriched in metabolic pathways, suggesting that polyploid hepatocytes possess more active metabolic functions.

Compared to FACS-sorted scRNA-seq or snRNA-seq [28, 29], SCIPI enables the recovery of a greater number of qualified cells for analysis and achieves higher accuracy in ploidy classification, though it exhibits a relatively lower RNA capture efficiency. Relative to image-based ploidy identification methods such as MERFISH [51], SCIPI offers distinct advantages. In tissue sections used for MERFISH, nuclei are three-dimensional and only partially visible, preventing accurate conversion of nuclear area to total DNA content, relying instead on nuclear count to define “multinucleated/mononucleated” status. In contrast, SCIPI dissociates tissue and spreads cells onto a two-dimensional plane, allowing the acquisition of complete, unobstructed nuclear images. By combining bright-field cell contour recognition with DAPI-based nuclear area measurement, it directly correlates nuclear area with DNA content, thereby enabling precise ploidy determination and detailed karyotype analysis of polyploid cells. However, this approach inevitably results in the loss of native tissue microenvironmental information. Furthermore, while MERFISH is limited to a predefined panel of 317 genes focused on markers for hepatocytes and non-parenchymal cells, SCIPI is built on a universal high-throughput sequencing platform, enabling whole-transcriptome analysis. This comprehensive coverage provides a more complete molecular interpretation of functional differences associated with ploidy.

In summary, the SCIPI method provides a novel technical approach for in-depth investigation of the regulatory mechanisms of hepatic polyploidy in liver regeneration, development, and various liver diseases [22, 25, 26]. This methodological advancement also opens new avenues for studying polyploid cells—such as cardiomyocytes [3, 52–54] and skeletal muscle fibers [55–57]—which have been challenging to profile reliably with single-cell transcriptomics.

## Methods

### Hepatocytes isolation and preparation

Primary hepatocytes were isolated from C57BL/6 8-week-old male mice using a modified two-step collagenase perfusion protocol [37–43], in which mouse livers were perfused via the inferior vena cava sequentially with perfusion buffer consisting of 0.05 mM ethylenediaminetetraacetic acid (EDTA)(Thermo, AM9260G) and Ca²⁺-free HBSS (Gibco, 14175–095), and digestion buffer (0.1 mg/ml collagenase IV, Sigma, C5138-5 G; and 1.6 mM CaCl₂, Sigma, 793639–500 G). The digested liver tissue was transferred to ice-cold Dulbecco’s Modified Eagle’s Medium (DMEM)(Cytiva, SH30243.01) for gentle cell dissociation. The cell suspension was sequentially filtered through 70 and 40 µm cell strainers to remove residual clusters. The cell suspension was centrifuged at 50 × *g* for 3 min to purify the hepatocytes. The cell suspension was layered over a 50% Percoll solution (Sigma, P7828-500ML) and centrifuged at 100 × *g* for 10 min to isolate viable hepatocytes. The pelleted hepatocytes were resuspended in DMEM, washed twice, and finally resuspended in Dulbecco’s Phosphate-Buffered Saline (DPBS)(Sigma, D8537-500ML) with 0.04% BSA (Aladdin, A116563-25 g). Hepatocytes were counted with a hemocytometer, and cell viability was confirmed to be >96%. Hepatocytes were immediately processed for Stereo-cell sequencing to prevent cell death associated with prolonged storage (≤1 h post-isolation at 4°C).

### Stereo chip preprocessing for hepatocyte loading and microimaging

The manufacturer’s kits were used (Stereo-seq Spatiotemporal Single Cell Transcriptome Reagent Kit, STOmics, STC1000R; Stereo-seq Spatiotemporal Single Cell Barcode Amplification Reagent Kit, STOmics, STC1000P), and the experimental procedures were performed according to the previously reported protocol [33]. The Stereo chip had been placed in a 24-well plate was washed twice with nuclease-free water. Transfer the chip to a new well, coat it with 400 µl of 0.01% poly-l-lysine solution, and incubate it on an orbital shaker (50–250 rpm) at room temperature (RT) for 1 h. After aspiration of the coating solution, the chip was rinsed twice with nuclease-free water and moved to a clean well. After aspiration of the coating solution, the chip was rinsed twice with nuclease-free water and moved to a clean well. A suspension of hepatocytes in 20 µl of DPBS containing 0.04% BSA was carefully applied to the chip surface. It is critical to gently spread the suspension with a pipette tip for even cell distribution while avoiding direct contact with the chip surface. The chip was incubated at RT for 15 min to allow cell attachment, followed by fixation in ice-cold methanol (−20°C) for 30 min. Fixed cells are subsequently stained with DAPI solution (Thermo, 62248), with systematic exclusion of glycerol in mounting protocols to prevent hydrodynamic displacement of large polyploid hepatocytes during high-resolution imaging. Final micrographs are acquired using a Motic PA53 FS6 microscope operating synchronized bright-field and DAPI fluorescence channels, ensuring co-registered.

### Library construction and sequencing

The Stereo-cell library construction for hepatocytes refers to Liao et al. in 2025 [33]. After imaging, hepatocytes were permeabilized with 0.1% pepsin at 37°C for 30 s. RNA released from the permeabilized cells was captured by the chip’s DNA Nanoballs (DNBs) and subsequently reverse transcribed overnight at 42°C. Chips containing cDNA were then treated overnight at 55°C for 3 h with a cDNA release mixture. The released cDNA was collected and amplified using sequencing sample barcode primers to generate Stereo-cell libraries. These libraries were quantified using the Qubit dsDNA Assay Kit (Thermo, Q32854) and either the Agilent 2100 Bioanalyzer or Qsep, respectively. Eighty nanograms of DNA samples was used as input for DNA nanoball (DNB) generation. The DNB library was sequenced on an MGI DNBSEQ-Tx platform using ∼50 bp paired-end sequencing.

### Immunohistochemistry and analysis

For liver frozen sections, all experiments were performed using the BeyoTSA™ Triple-Label Four-Color Multiplex Fluorescent Staining Kit (Beyotime, P1351S). Samples were fixed and permeabilized separately at RT. Endogenous peroxidase was blocked with blocking solution (P0100A) at RT for 10–15 min. Nonspecific background was blocked with appropriate blocking buffer (P0102) at RT. β-Catenin antibody (Cell Signaling, 9582) was diluted properly and incubated with samples at RT for 2 h. Corresponding secondary antibody was diluted and incubated at RT for 60 min. TSA staining working solution was added for 10 min at RT in the dark. Subsequently, DAPI staining solution was added for nuclear counterstaining at RT in the dark for 5 min. Staining results were observed under a fluorescence microscope. After completing microscopic imaging, 3 fields of view were randomly selected for each mouse replicate. Following the same workflow as Stereo-cell (without the need for registration), we performed cell segmentation on β-Catenin fluorescence images and nuclear segmentation on DAPI fluorescence images, respectively. Subsequently, ploidy identification was conducted using the identical method, and the proportions of different ploidy types were quantified separately.

### Flow cytometry and analysis

For hepatocyte flow cytometry sorting, 1–2 × 10^6^; prepared hepatocyte suspension was adjusted to 500 µl and placed in a 15 ml centrifuge tube. Ice-cold absolute ethanol was slowly added to 2 ml (final concentration 75%), and the cells were fixed at 4°C for 60 min. After centrifugation at 100 × *g* for 5 min, the supernatant was discarded. Then, 1 ml of 1 × phosphate-buffered saline (PBS)(Gibco, 10010001) was added to resuspend the cells, followed by 2 additional centrifugation washes. The supernatant was discarded, and 2 µg/ml DAPI staining solution was added; after mixing, the cells were stained at 4°C for 20 min in the dark. Finally, the cells were washed twice with 1 × PBS, filtered through a 40 µm filter, and hepatocyte populations with different fluorescence intensities were sorted by flow cytometry. For each ploidy type (2c, 4c, 8c) across different batches, cells were evenly spread onto glass slides and imaged under a microscope. Sorting was performed with imaging via the BASIC channel and DAPI channel. Ultimately, cell images and nuclear images for each ploidy type were acquired. Subsequently, we completed cell segmentation and assign nuclei to their corresponding cells. This process yielded *_nucle_info.csv files, which contain metrics such as the number of nuclei per cell and nuclear area. Cells with <1 or >2 nuclei were first filtered out, and the average nuclear area was then calculated. By integrating the mean fluorescence intensity of different ploidy populations previously determined via flow cytometry, the correlation between nuclear area and DNA content can be directly assessed.

### Raw sequencing data preprocessing

After sequencing, fastq format files will be obtained, which contain DNA base sequences, spatial coordinate sequences, and unique molecular identifier (UMI) sequences, after processing with SAW [44], we can obtain GEM-format files that have undergone sequence demultiplexing and alignment. This file contains Gene IDs, gene coordinates (x, y), molecular identifier count (MID Count), and exon counts. Based on this information, we can generate *in situ* UMI counts image (UMI image) with script gem2mask.py.

### Image registration and preprocessing

Image registration was performed using FIJI ImageJ [58]. Since the microscope stage remained stationary during sequential imaging, the brightfield and DAPI images were inherently aligned. Thus, ImageJ was solely used to register the DAPI image with the corresponding UMI image. Both DAPI and brightfield images were converted from red, green, blue (RGB) formatto 8-bit to reduce memory usage, and brightness was adjusted to achieve consistency between the DAPI and UMI signals. A TrackEM2 project was created, and the folder containing the brightfield, DAPI, and UMI images was imported. Switch the active image to the UMI image. Unlink the UMI image from other images to ensure that adjustments to the DAPI image do not affect the UMI image. Maintain the link between the DAPI and brightfield images. Select the DAPI layer as the active layer. Configure the UMI image as the red channel and DAPI as the green channel in the layer settings. Register the DAPI image using the Transform tool, after registration, validate the alignment by zooming into multiple fields of view to ensure that the majority of the DAPI signal (green channel) overlaps with the UMI regions (red channel). Export and save both the DAPI and brightfield images in TIFF format.

### Cell segmentation and ploidy analysis

Perform cell segmentation on both the DAPI and brightfield images that have been co-registered with the UMI image. For the brightfield image, use Cellpose with the cyto3 model [45]. Typically, first run the software to determine the diameter using the calibrate option for automatic assessment. Save the results as PNG-format images. For the DAPI image, use StarDist with the 2D_versatile_fluo model [59] to generate DAPI masks. Save the output as CSV-format files. Next, identify hepatocytes polyploidy. First, perform cell segmentation on the GEM file using the results from Cellpose. The processed GEM file will include an additional mask column, where different mask values represent individual cells. Calculate the correspondence between DAPI masks and cell segmentation results. We define that if ≥50% of a DAPI mask’s area overlaps with a cell region, the DAPI mask is assigned to that cell. Record the correspondence between DAPI masks and cell masks, DAPI areas, DAPI counts per cell, and other relevant nuclear information in a new DataFrame, which is then saved. Generate a single-cell matrix file from the new GEM file, which can then be imported into Seurat or Scanpy for downstream analysis. Nuclear information is incorporated into the metadata.

### Pseudotime analysis of hepatic zonation

To construct the pseudotime of liver zonation, we utilized the AddModuleScore function in Seurat software. Specifically, we defined 2 gene modules: the CV module consisting of (*Cyp2e1, Glul, Cyp1a2, Cyb5a, Cyp2a5*, and *Cyp27a1*) and the periportal (PV) module consisting of (*Cyp2f2, Cdh1, Hal, Sds, Aldob*, and *Gls2*). Subsequently, we calculated the Zonation Score as the difference between the PV score and the CV score (PV score − CV score). This Zonation Score was then evenly divided into 20 bins to generate the pseudotime score for liver zonation.

## Additional files

**Supplementary Figure 1:** Flow cytometric DNA content analysis combined with nuclear area assessment. (a) Correlation between two SCIPI-generated biological replicates (hep56_H8 and hep56_M1) in P56 mouse liver; *R*² = 0.87. (b–e) Flow cytometry analysis of P56 C57BL/6 mouse hepatocytes. Hepatocyte gating (89.4%) in (b). Single-cell gating (100%) in (c). Intact cell gating (89.9%) in (d). DNA content analysis showing diploid (2c, 15.5%), tetraploid (4c, 59.6%), and octoploid (8c, 22.7%) cells in (e). (f) Brightfield, DAPI, and merged images of flow-sorted cell fragments. Scale bar, 100 μm. (g) The fraction of mononuclear (red, nuclear count = 1) and binuclear (blue, nuclear count = 2) cells among flow-sorted 2c, 4c, and 8c hepatocytes (*n* = 3 biologically independent P56 C57BL/6 mice).

**Supplementary Figure 2:** Integrated analysis of stereo-cell data and published scRNA-seq datasets. (a) UMAP plots of integrated scRNA-seq data showing the zonation annotation. (b) UMAP plots of integrated scRNA-seq data showing the distribution of ploidy subpopulations across mononuclear diploid (2n×1), mononuclear tetraploid (4n×1), binuclear tetraploid (2n×2), mononuclear octoploid (8n×1), binuclear octoploid (4n×2), and binucleated hexadecaploid (8n×2) cells. (c–h) Integrated scRNA-seq UMAP plots displaying zonation gene expression.

**Supplementary Figure 3:** GO enrichment analysis for the specific effects of nuclear and cellular ploidy. (a–c) Cnetplot of GO enrichment analysis for DEGs in binuclear tetraploid (2n×2), mononuclear octoploid (8n×1), and binuclear octoploid (4n×2) cells. (d) Violin plots showing the normalized expression levels of representative liver-specific genes across mononuclear diploid (2n×1), mononuclear tetraploid (4n×1), binuclear tetraploid (2n×2), mononuclear octoploid (8n×1), and binuclear octoploid (4n×2) cells. Kruskal–Wallis overall test followed by Wilcoxon pairwise comparisons with Benjamini–Hochberg (BH) correction (ns, not significant; *P < 0.05; **P < 0.01; ***P < 0.001).

## Availability of source code and requirements

Project name: Hep_Ploidy_protocol

Project homepage: https://github.com/JeffLuo9/Hep_Ploidy_protocol

License: MIT license

Operating system: CentOS Stream 10 (Linux 6.12.0-126.el10.x86_64); Windows 11

Programming language: Python; R; Shell.

Package management: Conda; pip (Python); Bioconductor/CRAN (R)

Hardware requirements: Minimum: 64 GB RAM, 4-core CPU, 256 GB SSD; Recommended: 64 GB RAM, 16-core CPU, NVIDIA RTX 5090 GPU, 1 TB SSD

RRID:SCR_027937

BioTools ID: scipi.

## Supplementary Material

giag023_Supplemental_Files

giag023_Authors_Response_To_Reviewer_Comments_original_submission

giag023_GIGA-D-25-00452_original_submission

giag023_GIGA-D-25-00452_Revision_1

giag023_Reviewer_1_Report_original_submission Reviewer 1 -- 12/30/2025

giag023_Reviewer_1_Report_revision_1 Reviewer 1 -- 2/16/2026

giag023_Reviewer_2_Report_original_submission Reviewer 2 -- 1/4/2026

giag023_Reviewer_2_Report_revision_1 Reviewer 2 -- 2/17/2026

## Data Availability

Raw sequencing data supporting the findings of this study have been deposited in the CNGB Nucleotide Sequence Archive (CNSA) under accession number CNP0008257. Supplementary material, including processed data, imaging data, and code used to generate figures, are available in the GigaDB [60].

## References

[1] Rios A C, Fu N Y, Jamieson P R et al. Essential role for a novel population of binucleated mammary epithelial cells in lactation. Nat Commun. 2016;7:11400. 10.1038/ncomms11400. 27102712 PMC4844753

[2] Unhavaithaya Y, Orr-Weaver T L. Polyploidization of glia in neural development links tissue growth to blood-brain barrier integrity. Genes Dev. 2012;26:31–36. 10.1101/gad.177436.111. 22215808 PMC3258963

[3] Anatskaya O V, Vinogradov A E. Genome multiplication as adaptation to tissue survival: evidence from gene expression in mammalian heart and liver. Genomics. 2007;89:70–80. 10.1016/j.ygeno.2006.08.014. 17029690

[4] Zanet J, Freije A, Ruiz M et al. A mitosis block links active cell cycle with human epidermal differentiation and results in endoreplication. PLoS One. 2010;5:e15701. 10.1371/journal.pone.0015701. 21187932 PMC3004957

[5] Sher N, Von Stetina J R, Bell G W et al. Fundamental differences in endoreplication in mammals and *Drosophila* revealed by analysis of endocycling and endomitotic cells. Proc Natl Acad Sci USA. 2013;110:9368–9373. 10.1073/pnas.1304889110. 23613587 PMC3677442

[6] Anatskaya O V, Vinogradov A E, Kudryavtsev B N. Hepatocyte polyploidy and metabolism/life-history traits: hypotheses testing. J Theor Biol. 1994;168:191–199. 10.1006/jtbi.1994.1098. 8022197

[7] Gentric G, Desdouets C. Liver polyploidy: Dr Jekyll or Mr Hide?. Oncotarget. 2015;6:8430–8431. 10.18632/oncotarget.3809. 25940698 PMC4496158

[8] Wang M J, Chen F, Lau JTY et al. Hepatocyte polyploidization and its association with pathophysiological processes. Cell Death Dis. 2017;8:e2805. 10.1038/cddis.2017.167. 28518148 PMC5520697

[9] Margall-Ducos G, Celton-Morizur S, Couton D et al. Liver tetraploidization is controlled by a new process of incomplete cytokinesis. J Cell Sci. 2007;120:3633–3639. 10.1242/jcs.016907. 17895361

[10] Fang J, de Bruin A, Villunger A et al. Cellular polyploidy in organ homeostasis and regeneration. Protein Cell. 2023;14:560–578. 10.1093/procel/pwac064. 37526344 PMC10392032

[11] Fortier M, Celton-Morizur S, Desdouets C. Incomplete cytokinesis/binucleation in mammals: the powerful system of hepatocytes. Methods Cell Biol. 2017;137:119–142. 10.1016/bs.mcb.2016.04.006. 28065301

[12] Donne R, Saroul-Aïnama M, Cordier P et al. Polyploidy in liver development, homeostasis and disease. Nat Rev Gastroenterol Hepatol. 2020;17:391–405. 10.1038/s41575-020-0284-x. 32242122

[13] Guidotti J E, Brégerie O, Robert A et al. Liver cell polyploidization: a pivotal role for binuclear hepatocytes. J Biol Chem. 2003;278:19095–19101. 10.1074/jbc.M300982200. 12626502

[14] Celton-Morizur S, Merlen G, Couton D et al. The insulin/akt pathway controls a specific cell division program that leads to generation of binucleated tetraploid liver cells in rodents. J Clin Invest. 2009;119:1880–1887. 10.1172/jci38677. 19603546 PMC2701880

[15] Chao H W, Doi M, Fustin J M et al. Circadian clock regulates hepatic polyploidy by modulating Mkp1-Erk1/2 signaling pathway. Nat Commun. 2017;8:2238. 10.1038/s41467-017-02207-7. 29269828 PMC5740157

[16] Pandit S K, Westendorp B, Nantasanti S et al. E2F8 is essential for polyploidization in mammalian cells. Nat Cell Biol. 2012;14:1181–1191. 10.1038/ncb2585. 23064264

[17] Chen H Z, Ouseph M M, Li J et al. Canonical and atypical E2Fs regulate the mammalian endocycle. Nat Cell Biol. 2012;14:1192–1202. 10.1038/ncb2595. 23064266 PMC3616487

[18] Wilkinson P D, Delgado E R, Alencastro F et al. The polyploid state restricts hepatocyte proliferation and liver regeneration in mice. Hepatology. 2019;69:1242–1258. 10.1002/hep.30286. 30244478 PMC6532408

[19] Wang M J, Chen F, Li J X et al. Reversal of hepatocyte senescence after continuous in vivo cell proliferation. Hepatology. 2014;60:349–361. 10.1002/hep.27094. 24711261

[20] Gorla G R, Malhi H, Gupta S. Polyploidy associated with oxidative injury attenuates proliferative potential of cells. J Cell Sci. 2001;114:2943–2951. 10.1242/jcs.114.16.2943. 11686298

[21] Malhi H, Gorla G R, Irani A N et al. Cell transplantation after oxidative hepatic preconditioning with radiation and ischemia-reperfusion leads to extensive liver repopulation. Proc Natl Acad Sci USA. 2002;99:13114–13119. 10.1073/pnas.192365499. 12244212 PMC130595

[22] Gentric G, Maillet V, Paradis V et al. Oxidative stress promotes pathologic polyploidization in nonalcoholic fatty liver disease. J Clin Invest. 2015;125:981–992. 10.1172/jci73957. 25621497 PMC4362240

[23] Yin K, Büttner M, Deligiannis I K et al. Polyploidisation pleiotropically buffers ageing in hepatocytes. J Hepatol. 2024;81:289–302. 10.1016/j.jhep.2024.03.043. 38583492

[24] Zhang S, Zhou K, Luo X et al. The polyploid state plays a tumor-suppressive role in the liver. Dev Cell. 2018;44:447–59..e447. 10.1016/j.devcel.2018.01.010. 29429824 PMC5828993

[25] Bu W, Sun X, Xue X et al. Early onset of pathological polyploidization and cellular senescence in hepatocytes lacking RAD51 creates a pro-fibrotic and pro-tumorigenic inflammatory microenvironment. Hepatology. 2025;81:491–508. 10.1097/hep.0000000000000821. 38466833 PMC11737125

[26] Wang J, Huang X, Zheng D et al. PRMT5 determines the pattern of polyploidization and prevents liver from cirrhosis and carcinogenesis. J Genet Genom. 2023;50:87–98. 10.1016/j.jgg.2022.04.008. 35500745

[27] Tanami S, Ben-Moshe S, Elkayam A et al. Dynamic zonation of liver polyploidy. Cell Tissue Res. 2017;368:405–410. 10.1007/s00441-016-2427-5. 27301446

[28] Richter M L, Deligiannis I K, Yin K et al. Single-nucleus RNA-seq2 reveals functional crosstalk between liver zonation and ploidy. Nat Commun. 2021;12:4264. 10.1038/s41467-021-24543-5. 34253736 PMC8275628

[29] Yang L, Wang X, Zheng J X et al. Determination of key events in mouse hepatocyte maturation at the single-cell level. Dev Cell. 2023;58:1996–2010.e6..e1996. 10.1016/j.devcel.2023.07.006. 37557173

[30] Wei X, Fu S, Li H et al. Single-cell stereo-seq reveals induced progenitor cells involved in axolotl brain regeneration. Science. 2022;377:eabp9444. 10.1126/science.abp9444. 36048929

[31] Chen A, Liao S, Cheng M et al. Spatiotemporal transcriptomic atlas of mouse organogenesis using DNA nanoball-patterned arrays. Cell. 2022;185:1777–1792..e1721. 10.1016/j.cell.2022.04.003. 35512705

[32] Xu J, Guo P, Hao S et al. A spatiotemporal atlas of mouse liver homeostasis and regeneration. Nat Genet. 2024;56:953–969. 10.1038/s41588-024-01709-7. 38627598

[33] Liao S, Zhou X, Liu C et al. Stereo-cell: spatial enhanced-resolution single-cell sequencing with high-density DNA nanoball-patterned arrays. Science. 2025;389(6762):eadr0475. 10.1126/science.adr0475. 40839715

[34] Zhou L, Peng X, Chen M et al. Unveiling patterns in spatial transcriptomics data: a novel approach utilizing graph attention autoencoder and multiscale deep subspace clustering network. GigaScience. 2025;14:giae103. 10.1093/gigascience/giae103. PMC11727722 39804726

[35] Liu X, Qu C, Liu C et al. StereoSiTE: a framework to spatially and quantitatively profile the cellular neighborhood organized iTME. GigaScience. 2024;13:giae078. 10.1093/gigascience/giae078. PMC11503478 39452614

[36] Jiahui L, Shijie H, Yongqing Y et al. SCIPI: a tool for hepatocyte ploidy subpopulation identification. 10.48546/WORKFLOWHUB.WORKFLOW.2079.3. Accessed 9 February 2026.

[37] Berry M N, Friend D S. High-yield preparation of isolated rat liver parenchymal cells: a biochemical and fine structural study. J Cell Biol. 1969;43:506–520. 10.1083/jcb.43.3.506. 4900611 PMC2107801

[38] MacParland S A, Liu J C, Ma X Z et al. Single cell RNA sequencing of human liver reveals distinct intrahepatic macrophage populations. Nat Commun. 2018;9:4383. 10.1038/s41467-018-06318-7. 30348985 PMC6197289

[39] Casciano D A . Development and utilization of primary hepatocyte culture systems to evaluate metabolism, DNA binding, and DNA repair of xenobiotics. Drug Metab Rev. 2000;32:1–13. 10.1081/dmr-100100561. 10711405

[40] Klaunig J E, Goldblatt P J, Hinton D E et al. Mouse liver cell culture. I. Hepatocyte isolation. In Vitro. 1981;17:913–925. 10.1007/bf02618288. 6273298

[41] Li W C, Ralphs K L, Tosh D. Isolation and culture of adult mouse hepatocytes. Methods Mol Biol. 2010;633:185–196. 10.1007/978-1-59745-019-5_13. 20204628

[42] Severgnini M, Sherman J, Sehgal A et al. A rapid two-step method for isolation of functional primary mouse hepatocytes: cell characterization and asialoglycoprotein receptor based assay development. Cytotechnology. 2012;64:187–195. 10.1007/s10616-011-9407-0. 22105762 PMC3279583

[43] Charni-Natan M, Goldstein I. Protocol for primary mouse hepatocyte isolation. STAR Protocols. 2020;1:100086. 10.1016/j.xpro.2020.100086. 33111119 PMC7580103

[44] Gong C, Li S, Wang L et al. SAW: an efficient and accurate data analysis workflow for stereo-seq spatial transcriptomics. GigaByte. 2024;2024:1. 10.46471/gigabyte.111. PMC10905255 38434930

[45] Stringer C, Wang T, Michaelos M et al. Cellpose: a generalist algorithm for cellular segmentation. Nat Methods. 2021;18:100–106. 10.1038/s41592-020-01018-x. 33318659

[46] Saeter G, Lee C Z, Schwarze P E et al. Changes in ploidy distributions in human liver carcinogenesis. J Natl Cancer Inst. 1988;80:1480–1484. 10.1093/jnci/80.18.1480. 2846856

[47] Duncan A W, Taylor M H, Hickey R D et al. The ploidy conveyor of mature hepatocytes as a source of genetic variation. Nature. 2010;467:707–710. 10.1038/nature09414. 20861837 PMC2967727

[48] Duncan A W, Hanlon Newell A E, Smith L et al. Frequent aneuploidy among normal human hepatocytes. Gastroenterology. 2012;142:25–28. 10.1053/j.gastro.2011.10.029. 22057114 PMC3244538

[49] Halpern K B, Shenhav R, Matcovitch-Natan O et al. Single-cell spatial reconstruction reveals global division of labour in the mammalian liver. Nature. 2017;542:352–356. 10.1038/nature21065. 28166538 PMC5321580

[50] Ben-Moshe S, Itzkovitz S. Spatial heterogeneity in the mammalian liver. Nat Rev Gastroenterol Hepatol. 2019;16:395–410. 10.1038/s41575-019-0134-x. 30936469

[51] Watson B R, Paul B, Rahman R U et al. Spatial transcriptomics of healthy and fibrotic human liver at single-cell resolution. Nat Commun. 2025;16:319. 10.1038/s41467-024-55325-4. 39747812 PMC11697218

[52] Bensley J G, De Matteo R, Harding R et al. Three-dimensional direct measurement of cardiomyocyte volume, nuclearity, and ploidy in thick histological sections. Sci Rep. 2016;6:23756. 10.1038/srep23756. 27048757 PMC4822151

[53] Østergaard K H, Baandrup U T, Wang T et al. Left ventricular morphology of the giraffe heart examined by stereological methods. Anat Rec. 2013;296:611–621. 10.1002/ar.22672. 23420662

[54] Hirose K, Payumo A Y, Cutie S et al. Evidence for hormonal control of heart regenerative capacity during endothermy acquisition. Science. 2019;364:184–188. 10.1126/science.aar2038. 30846611 PMC6541389

[55] Millay D P . Regulation of the myoblast fusion reaction for muscle development, regeneration, and adaptations. Exp Cell Res. 2022;415:113134. 10.1016/j.yexcr.2022.113134. 35367215 PMC9058940

[56] Petrany M J, Swoboda C O, Sun C et al. Single-nucleus RNA-seq identifies transcriptional heterogeneity in multinucleated skeletal myofibers. Nat Commun. 2020;11:6374. 10.1038/s41467-020-20063-w. 33311464 PMC7733460

[57] Sun C, Swoboda C O, Morales F M et al. Lineage tracing of nuclei in skeletal myofibers uncovers distinct transcripts and interplay between myonuclear populations. Nat Commun. 2024;15:9372. 10.1038/s41467-024-53510-z. 39477931 PMC11526147

[58] Schindelin J, Arganda-Carreras I, Frise E et al. Fiji: an open-source platform for biological-image analysis. Nat Methods. 2012;9:676–682. 10.1038/nmeth.2019. 22743772 PMC3855844

[59] Schmidt U, Weigert M, Broaddus C et al. Medical Image Computing and Computer assisted Intervention—MICCAI 2018 11071, 265–73. 10.1007/978-3-030-00934-2_30. Accessed 20 September 2018.

[60] Yongqing Y, Jiahui L, Yier C et al. Stereo-cell deciphers the spatial and functional heterogeneity of polyploid hepatocytes. GigaScience Database. 10.5524/102774. Accessed 28 February 2026. PMC13100898 41770024

