# Supplementary material for: Stereo-cell deciphers the spatial and functional heterogeneity of polyploid hepatocytes
Source: Gigascience. 2026 Mar 2;15:giag023. doi: 10.1093/gigascience/giag023 (PMC13100898; doi:10.1093/gigascience/giag023)
Supplement: giag023_Supplemental_Files [file giag023_supplemental_files.zip › Supplementary figure legend_260211.docx]

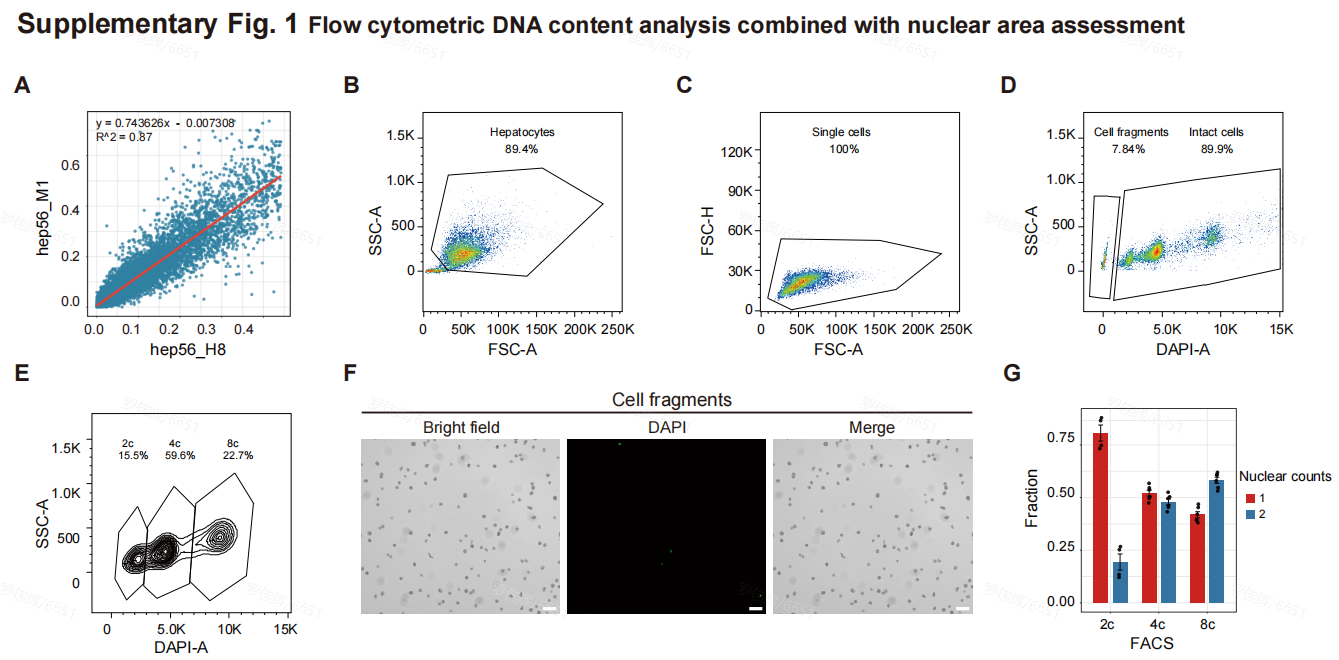


Supplementary figure 1 Flow cytometric DNA content analysis combined with nuclear area assessment. (A) Correlation between two SCIPI-generated biological replicates (hep56_H8 and hep56_M1) in P56 mouse liver; R²=0.87. (B-E) Flow cytometry analysis of P56 C57BL/6 mouse hepatocytes. Hepatocyte gating (89.4%) in (B). Single-cell gating (100%) in (C). Intact cell gating (89.9%) in (D). DNA content analysis showing diploid (2c, 15.5%), tetraploid (4c, 59.6%), and octoploid (8c, 22.7%) cells in (E). (F) Brightfield, DAPI, and merged images of flow-sorted cell fragments. Scale bar, 100 μm. (G) The fraction of mononuclear (red, nuclear count=1) and binuclear (blue, nuclear count=2) cells among flow-sorted 2c, 4c, and 8c hepatocytes (n=3 biologically independent P56 C57BL/6 mice).


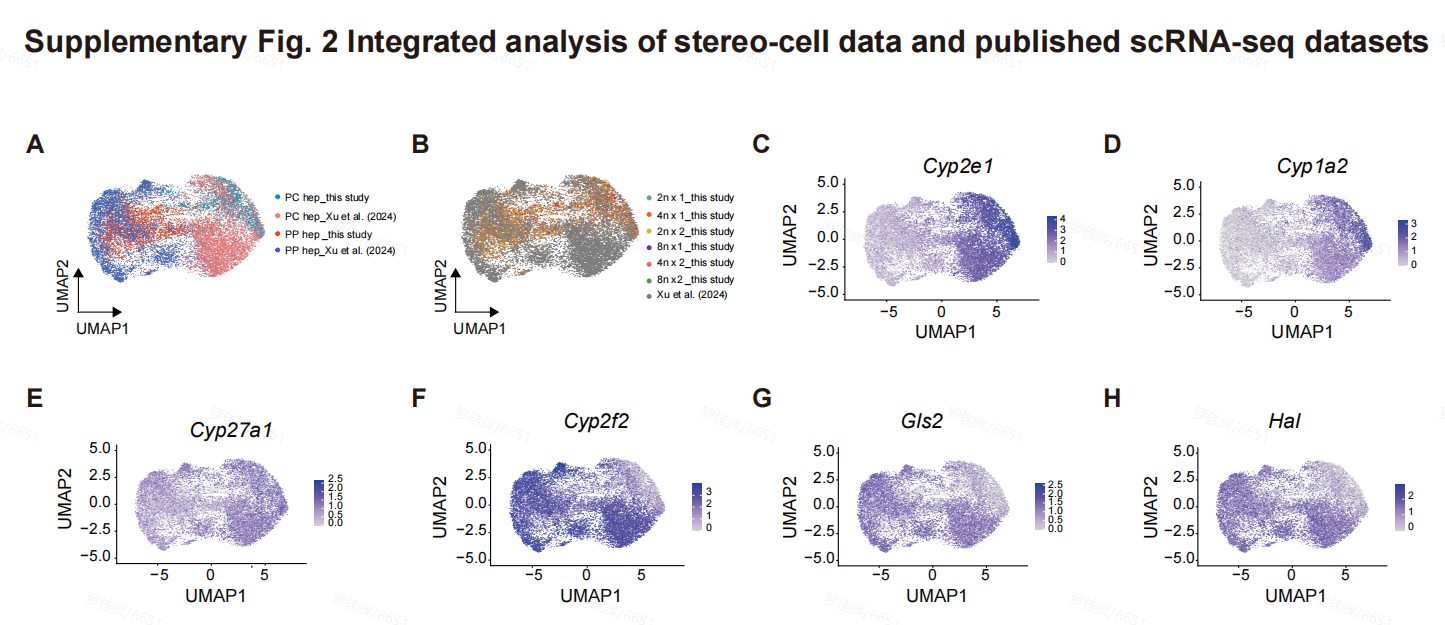


Supplementary figure 2 Integrated analysis of stereo-cell data and published scRNA-seq datasets. (A) UMAP plots of integrated scRNA-seq data showing the zonation annotation. (B) UMAP plots of integrated scRNA-seq data showing the distribution of ploidy subpopulations across mononuclear diploid (2n×1), mononuclear tetraploid (4n×1), binuclear diploid (2n×2), mononuclear octoploid (8n×1), and binuclear tetraploid (4n×2) cells. (C-H) Integrated scRNA-seq UMAP plots displaying zonation gene expression.


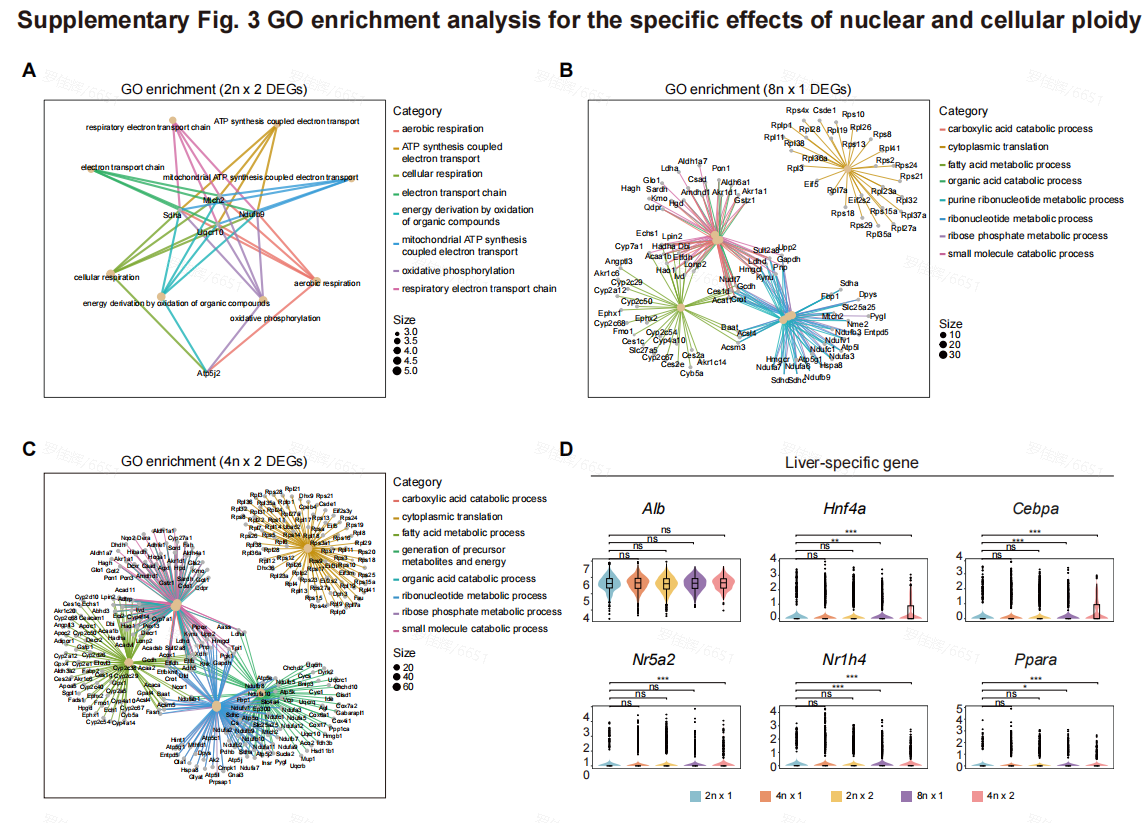


Supplementary figure 3 GO enrichment analysis for the specific effects of nuclear and cellular ploidy. (A-C) Cnetplot of GO enrichment analysis for DEGs in binuclear tetraploid (2n×2), mononuclear octoploid (8n×1), and binuclear octoploid (4n×2) cells. (D) Violin plots showing the normalized expression levels of representative liver-specific genes across mononuclear diploid (2n×1), mononuclear tetraploid (4n×1), binuclear diploid (2n×2), mononuclear octoploid (8n×1), and binuclear octoploid (4n×2) cells. Kruskal-Wallis overall test followed by Wilcoxon pairwise comparisons with Benjamini-Hochberg (BH) correction (ns: not significant; *p<0.05; **p<0.01; ***p<0.001).
