# Supplementary material for: Stereo-cell deciphers the spatial and functional heterogeneity of polyploid hepatocytes
Source: Gigascience. 2026 Mar 2;15:giag023. doi: 10.1093/gigascience/giag023 (PMC13100898; doi:10.1093/gigascience/giag023)

Supplementary Fig. 3 GO enrichment analysis for the specific effects of nuclear and cellular ploidy

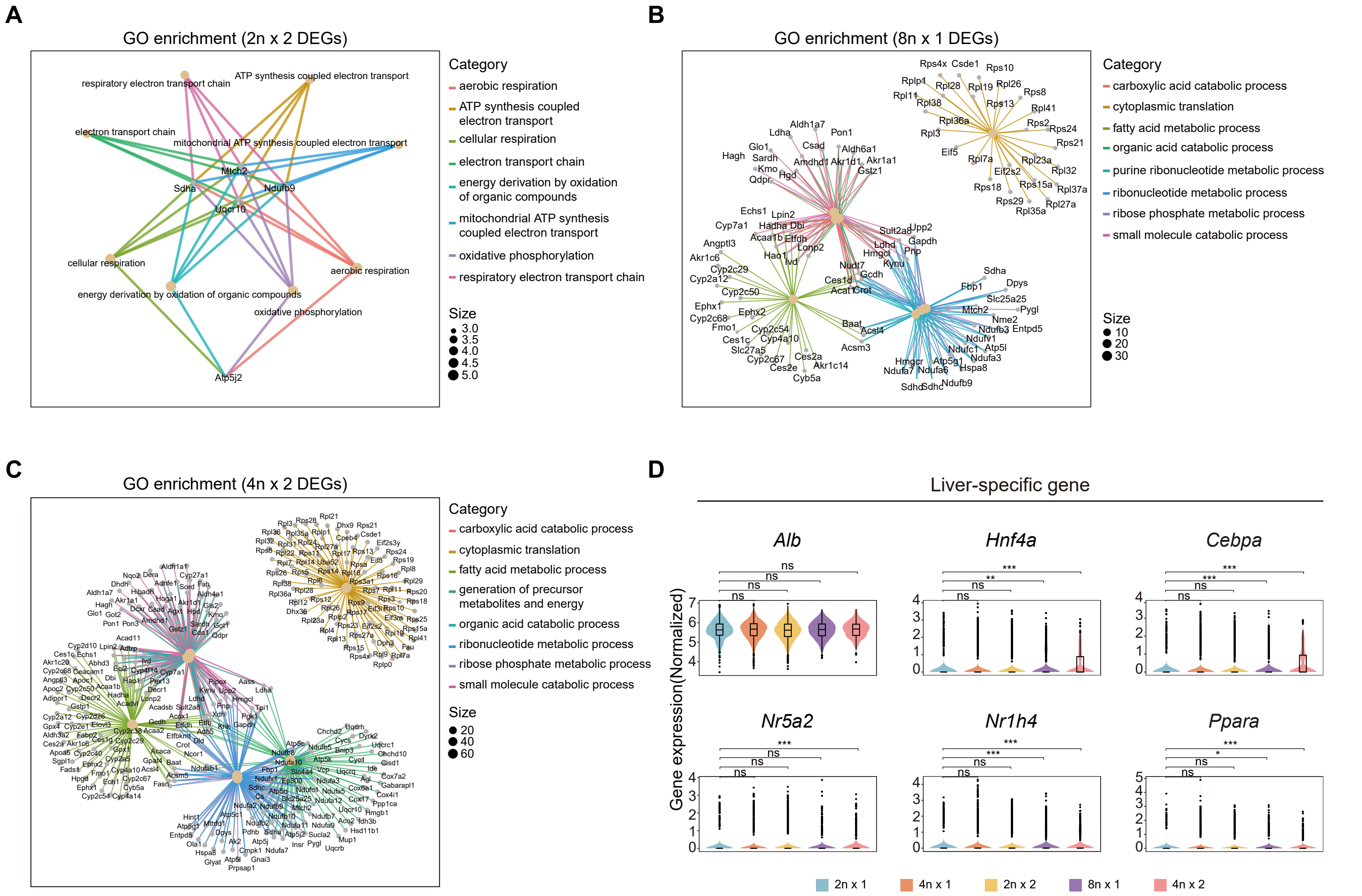

Supplement: giag023_Supplemental_Files [file giag023_supplemental_files.zip › Supplementary_Fig3_260226.pdf]
