# Supplementary material for: Stereo-cell deciphers the spatial and functional heterogeneity of polyploid hepatocytes
Source: Gigascience. 2026 Mar 2;15:giag023. doi: 10.1093/gigascience/giag023 (PMC13100898; doi:10.1093/gigascience/giag023)

GigaScience

Stereo-cell Deciphers the Spatial and Functional Heterogeneity of Polyploid Hepatocytes

--Manuscript Draft--

**Manuscript Number:**	GIGA-D-25-00452
**Full Title:**	Stereo-cell Deciphers the Spatial and Functional Heterogeneity of Polyploid Hepatocytes
**Article Type:**	Technical Note
**Funding Information:**	
**Abstract:**	Most mammalian cells are diploid, but polyploidy occurs in specific organs to enable specialized physiological functions—notably in hepatocytes, myocytes and mammary epithelial cells. However, elucidating these polyploid subtypes and profiling their distinct transcriptomes has long been constrained by technical limitations. To overcome this challenge, we used Stereo-cell, a spatially resolved single-cell sequencing method utilizing high-density DNA nanoball (DNB)-patterned arrays to allow for simultaneous transcriptome profiling and ploidy determination within the same cell, overcoming fundamental limitations of conventional approaches. Here, we detail a comprehensive method for the Stereo-cell imaging-based ploidy identification (SCIPI) pipeline. Our multimodal classification framework combines bright-field cell contour delineation, DAPI-guided nuclear area, and UMI-barcoded transcriptional profiling to precisely resolve four key hepatocyte subtypes—mononucleated diploid (2n × 1), binucleated tetraploid (2n × 2), mononucleated tetraploid (4n × 1), and binucleated octoploid (4n × 2)—and their differential gene expression pattern. This SCIPI strategy is broadly applicable to polyploidy tissues, unlocking unprecedented ploidy-resolved analysis across diverse biological scenarios.
**Corresponding Author:**	Shijie Hao<br>BGI Group<br>Shenzhen, --- Select a state --- CHINA
**Corresponding Author Secondary Information:**	
**Corresponding Author's Institution:**	BGI Group
**Corresponding Author's Secondary Institution:**	
**First Author:**	Shijie Hao
**First Author Secondary Information:**	
**Order of Authors:**	Shijie Hao
	Yongqing Yang
	Jiahui Luo
	Hong Wu
	Pengcheng Guo
**Order of Authors Secondary Information:**	
**Additional Information:**	
**Question**	**Response**
Are you submitting this manuscript to a special series or article collection?	No
**Experimental design and statistics**	Yes

Powered by Editorial Manager® and ProduXion Manager® from Aries Systems Corporation

Full details of the experimental design and statistical methods used should be given in the Methods section, as detailed in our Minimum Standards Reporting Checklist. Information essential to interpreting the data presented should be made available in the figure legends.<br><br>Have you included all the information requested in your manuscript?	
**Resources**<br><br>A description of all resources used, including antibodies, cell lines, animals and software tools, with enough information to allow them to be uniquely identified, should be included in the Methods section. Authors are strongly encouraged to cite Research Resource Identifiers (RRIDs) for antibodies, model organisms and tools, where possible.<br><br>Have you included the information requested as detailed in our Minimum Standards Reporting Checklist?	Yes
**Availability of data and materials**<br><br>All datasets and code on which the conclusions of the paper rely must be either included in your submission or deposited in publicly available repositories (where available and ethically appropriate), referencing such data using a unique identifier in the references and in the "Availability of Data and Materials" section of your manuscript.<br><br>Have you have met the above requirement as detailed in our Minimum Standards Reporting Checklist?	Yes
GigaScience has policies and guidelines in place for the use of generative AI-writing tools such as ChatGPT. If you have used such writing tools to assist with	No

writing the manuscript this must be declared and cited in the text. Authors should not list AI-writing tools and other AI-assisted technologies as an author or co-author and should acknowledge that they are fully responsible for text generated or refined by AI-writing tools.<p><br><br>A summary of use (particularly in the introduction or among methods) needs to be included at the end of the paper, and the outputs should also be included as a supplementary file hosted in GigaDB or other open repositories. Please read our guidelines for more information. <p><br><br>By submitting to GigaScience, you are aware of the journal's AI-writing tools policy, and if you have declared use of such tools below, you have acknowledged this where appropriate in your manuscript and have made a summary of use and outputs available. <p><br><b>AI-assisted writing tools have been used in the preparation of this manuscript?	

Manuscript Click here to access/download;Manuscript;GS_manuscript.docx

Stereo-cell Deciphers the Spatial and Functional Heterogeneity of Polyploid Hepatocytes

Yongqing Yang[1,2,†], Jiahui Luo[2,3,†], Hong Wu[1,2], Pengcheng Guo[2,3,4] and Shijie Hao[2,3,4,*]

[1]College of Life Sciences, University of Chinese Academy of Sciences, Beijing 100049, China.
[2]BGI Research, Hangzhou 310030, China.
[3]School of Biology and Biological Engineering, South China University of Technology, Guangzhou 510006, China.
[4]State Key Laboratory of Genome and Multi-omics Technologies, BGI Research, Hangzhou 310030, China.
[†]These authors contributed equally to this work.
[*]To whom correspondence should be addressed: Shijie Hao, E-mail: haoshijie@genomics.cn.

Abstract

Most mammalian cells are diploid, but polyploidy occurs in specific organs to enable specialized physiological functions—notably in hepatocytes, myocytes and mammary epithelial cells. However, elucidating these polyploid subtypes and profiling their distinct transcriptomes has long been constrained by technical limitations. To overcome this challenge, we used Stereo-cell, a spatially resolved single-cell sequencing method utilizing high-density DNA nanoball (DNB)-patterned arrays to allow for simultaneous transcriptome profiling and ploidy determination within the same cell, overcoming fundamental limitations of conventional approaches. Here, we detail a comprehensive method for the Stereo-cell imaging-based ploidy identification (SCIPI) pipeline. Our multimodal classification framework combines bright-field cell contour delineation, DAPI-guided nuclear area, and UMI-barcoded transcriptional profiling to precisely resolve four key hepatocyte subtypes—mononucleated diploid (2n × 1), binucleated tetraploid (2n × 2), mononucleated tetraploid (4n × 1), and binucleated octoploid (4n × 2)—and their differential gene expression pattern. This SCIPI strategy is broadly applicable to polyploidy tissues, unlocking unprecedented ploidy-resolved analysis across diverse biological scenarios.

Background

Polyploidy, a state characterized by cells containing more than two complete chromosome sets, represents an evolutionarily conserved biological phenomenon with particular significance in mammalian biology [1-5]. As the only organ capable of dynamically modulating its ploidy throughout ontogeny and in response to diverse stressors, the liver employs polyploidization as a fundamental mechanism governing growth regulation and functional adaptation [6, 7]. Livers predominantly contain binucleated tetraploid hepatocytes, a state arising through developmentally programmed cytokinesis failure during postnatal maturation [6, 8, 9]. This process initiates during the weaning transition, where diploid hepatocytes either complete normal cytokinesis to generate diploid progeny or undergo incomplete division to form binucleated tetraploids (2n x 2) [9-11]. Subsequent cell cycles in these binucleated tetraploids yield either mononucleated tetraploids via successful mitosis or binucleated octoploids (4n x 2) through recurrent cytokinesis failure [12, 13]. Molecularly, the insulin/AKT pathway [14], *Mkp1-Erk1/2* pathway[15] and E2F [16-18] transcription factors critically regulate polyploidization, with *E2f8* knockdown experiments demonstrating a polyploidy decrease in the liver. Beyond development, polyploidization escalates with aging [19] and cellular stress [20-22], serving as a genomic buffer that enhances organ resilience under physiological conditions but accelerates liver dysfunction in pathological contexts. This protective role is exemplified by haploinsufficiency of hepatic master regulators (HNF4A or CEBPA), which induces premature tetraploidization to mitigate age-related functional decline through non-random allelic segregation [23]. Critically, such tetraploidization concurrently establishes a tumor suppressive mechanism, wherein the polyploid state actively constrains genomic instability and oncogenic progression in the liver [24].

Pathologically, chronic insults trigger aberrant polyploidization that drive disease progression. In nonalcoholic fatty liver disease (NAFLD), oxidative stress activates the ATR/p53/p21 DNA damage checkpoint, promoting endoreplication that generates highly polyploid mononuclear cells which facilitate hepatocellular carcinoma (HCC) development [22]. Similarly, hepatocyte-specific RAD51 deletion induces G2/M arrest and accelerates

mononucleated polyploidy, precipitating premature senescence that progresses to fibrosis and HCC [25]. Complementing this, loss of PRMT5 causes p21 hyperaccumulation and endoreplication-dependent formation of pathological mononucleated polyploid hepatocytes, culminating in cirrhosis and HCC [26]. Collectively, these findings establish polyploidization as a double-edged sword—physiologically beneficial yet pathologically malignant when dysregulated.

The complexity and variability of hepatocyte ploidy states during liver development and disease are critically linked to hepatic physiology and pathology. Accurate identification of polyploidy patterns is therefore essential for understanding liver parenchymal biology. The process of hepatocyte polyploidization is accompanied by a concomitant increase in nuclear size[15]. Historically, two primary approaches have been used to study hepatocyte polyploidization, each with significant limitations. The conventional method involves co-staining liver cryosections with nuclear and plasma membrane markers, followed by ploidy classification through measurement of maximal nuclear diameter or area in situ[15, 27]. While this technique identifies all ploidy classes, it irrecoverably severs the critical connection between ploidy and transcriptional information. More recently, fluorescence-activated cell sorting (FACS) has been employed to isolate polyploid hepatocytes based on nuclear DNA content [28, 29]. Purified diploid (2n), tetraploid (4n), and higher-ploidy populations obtained through this method can subsequently undergo single-cell RNA-seq (scRNA-seq) or single-nucleus RNA-seq (snRNA-seq). However, despite generating transcriptional profiles, the FACS-based method fundamentally cannot discriminate between mononucleated and binucleated polyploid subtypes, a critical biological distinction lost during experiment.

To overcome the limitations of conventional polyploidy analysis methods, we developed Stereo-cell imaging-based ploidy identification (SCIPI) method, a comprehensive pipeline built upon the spatially resolved single-cell transcriptomics technology Stereo-cell[30-33]. This technology uniquely integrates spatial transcriptomics with imaging techniques to enable simultaneous transcriptome profiling and ploidy determination within the same cell. The SCIPI pipeline precisely classifies four key hepatocyte subtypes: mononucleated diploid ($2n \times 1$),

binucleated tetraploid (2n × 2), mononucleated tetraploid (4n × 1), and binucleated octoploid (4n × 2). Applying this framework, we reveal the distinct transcriptomic profiles of these four hepatocyte subtypes, providing a comprehensive resource for hepatocyte polyploid analysis.

Results

Integrated Stereo-cell Matrix of Gene Expression and Ploidy

Hepatocyte polyploidization is critical for liver development, functional maturation, and disease pathogenesis[12]. To decipher the functional implications of this undercharacterized biological phenomenon, we applied Stereo-cell[33] to generate single-cell matrix of gene expression including ploidy data. The Stereo-cell workflow for classifying polyploid hepatocytes (**Fig. 1A, B**) begins with isolating primary mouse hepatocytes via an optimized two-step collagenase perfusion protocol[34-40], which achieves high viability through enhanced enzymatic dissociation parameters. Primary mouse hepatocytes, characterized by large cell size, high aggregation propensity, and sensitivity to handling-induced death, require immediate processing post-isolation to ensure viability. For Stereo-cell library construction, approximately 5,000 hepatocytes per chip (minimum concentration 250 cells/μL) are strategically deposited onto poly-L-lysine pre-coated DNB-patterned Stereo chip. This coating leverages engineered electrostatic interactions between the cationic chip surface and anionic hepatocyte membranes to ensure consistent cellular adhesion. Following immobilization, cells are fixed with methanol and subsequently stained with DAPI for concurrent bright-field and fluorescence imaging to define cellular boundaries and nuclearity per cell. Stereo-cell libraries are prepared through template-switch oligo-mediated in situ reverse transcription, followed by in vitro cDNA amplification via PCR. Post-sequencing data processing incorporates standardized bioinformatic workflows for spatial barcode demultiplexing, UMI deduplication, and gene count matrix generation, ultimately producing spatially resolved transcriptional profiles.

We next established a multimodal analytical framework by integrating these spatially

resolved transcriptional profiles with the concurrently acquired bright-field images and DAPI-based nuclear quantification (**Fig. 1A, Hepatocyte ploidy identification**). This tripartite data architecture enables the systematic segmentation of individual hepatocytes, followed by automated ploidy classification through nuclear area quantification that distinguishes mononucleated diploid (2n × 1), binucleated tetraploid (2n × 2), mononucleated tetraploid (4n × 1), and binucleated octoploid (4n × 2) subtypes. Identity-matched matrices subsequently align these ploidy annotations with their corresponding transcriptomes while preserving spatial coordinates, permitting comprehensive ploidy-resolved single-cell analysis. The complete experimental workflow from hepatocyte isolation to sequencing typically requires 4 days. To ensure high-confidence identification of polyploid hepatocytes, we developed SCIPI method, a dedicated computational pipeline for Stereo-cell data analysis. SCIPI integrates the multimodal data (imaging and transcriptomics) described above, requiring approximately 1 day from raw data to final ploidy annotation.

To improve the identification accuracy of low-abundance hepatocyte ploidy subpopulations, we analyzed two biological replicates on 1 cm × 1 cm Stereo-chips. After quality control, which involved exclusion of trinuclear and tetranuclear hepatocytes, a total of approximately 5,000 cells were retained for downstream analysis. Both replicates exhibited consistent performance in classifying polyploid hepatocytes. Representative fields of view are presented in **Fig. 1B**.

Fig. 1 Comprehensive Stereo-cell Experimental Workflow for Hepatocytes

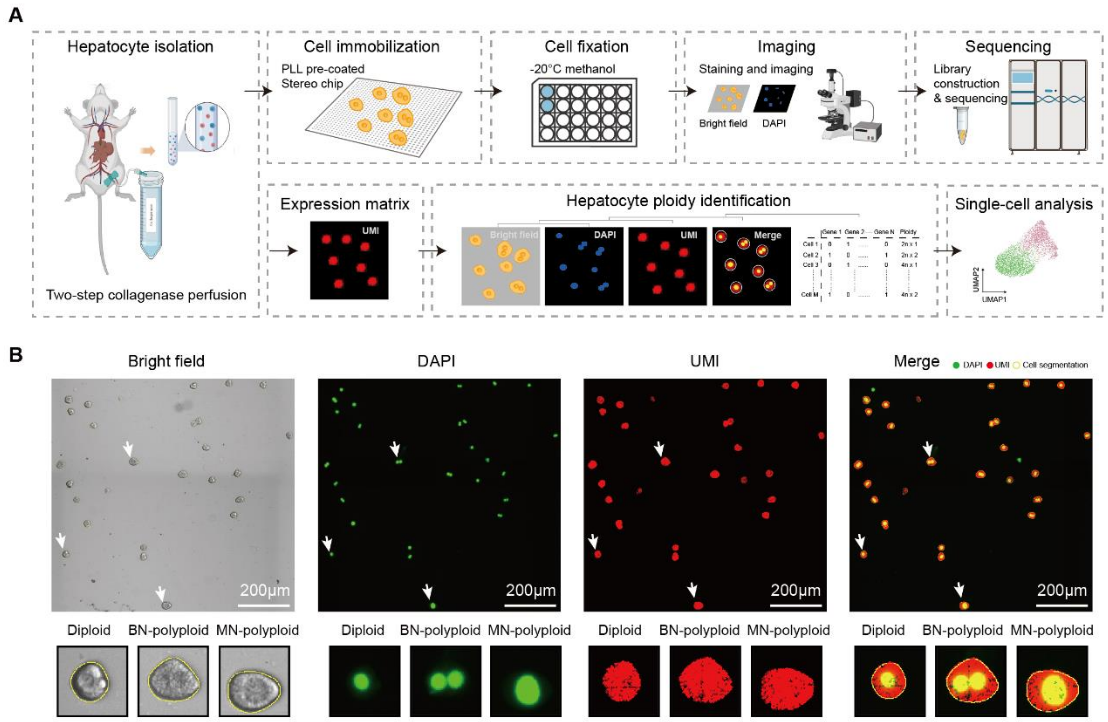

Figure1: Comprehensive Stereo-cell experimental workflow for hepatocytes. (A) Schematic of the Stereo-cell experimental workflow for hepatocytes. (B) Representative images show hepatocytes across multiple fields of view on the Stereo chip. Arrowheads indicate selected cells. BN-polyploid (binucleated polyploid hepatocyte); MN-polyploid (mononucleated polyploid hepatocyte). Scale bar: 200 µm.

Ploidy identification workflow and accuracy assessment

The Stereo-cell raw sequencing data was processed with SAW[41] to obtain GEM-format files that have undergone sequence demultiplexing and alignment. This file contains Gene IDs, gene coordinates (x, y), molecular identifier count (MID Count), and exon counts. Based on this information, we can generate in situ UMI counts image (UMI image) with script gem2mask.py. (**Fig. 1A, UMI**). Then, staining DAPI image was registered with UMI image. Perform cell segmentation on the DAPI image that has been registered to the UMI image and on the brightfield image. According to DAPI masks, cell masks, DAPI area and nuclearity per cell to classify hepatocyte ploidy subtypes (**Fig. 2A**). The classification between single-nucleus diploid and single-nucleus tetraploid uses k-means. In essence, k-means will converge on

several groups of points with the largest differences, which is similar to the sudden increase in nuclear area from diploid to tetraploid.

The accuracy of ploidy identification is primarily monitored through the following key steps. Firstly, ensure perfect registration between DAPI image and UMI image across multiple regions, with UMIs remaining untransformed. Switch between brightfield (green channel) and UMI (red channel) to confirm that cell boundaries in brightfield images align with UMI image in multiple selected regions. Secondly, assess cell size parameters during cell segmentation with Cellpose[42]. Manually verify that the majority of hepatocyte boundaries are accurately identified post-segmentation, and adjust size settings if necessary (Cellpose's automatic size estimation is generally reliable). Finally, cross-validate results using original DAPI, brightfield, and nuclear feature maps. Since the majority of cell nuclei are either single or double, and although a few reports have mentioned cells with three nuclei, for the sake of universality and accuracy, only cells with 1 or 2 nuclei are retained here for accuracy testing. Ensure the misidentification error within multiple randomly selected region is below 3 cells per region (**Fig. 2B**).

Fig. 2 SCIPI pipeline for spatially resolved hepatocyte polyploidy profiling

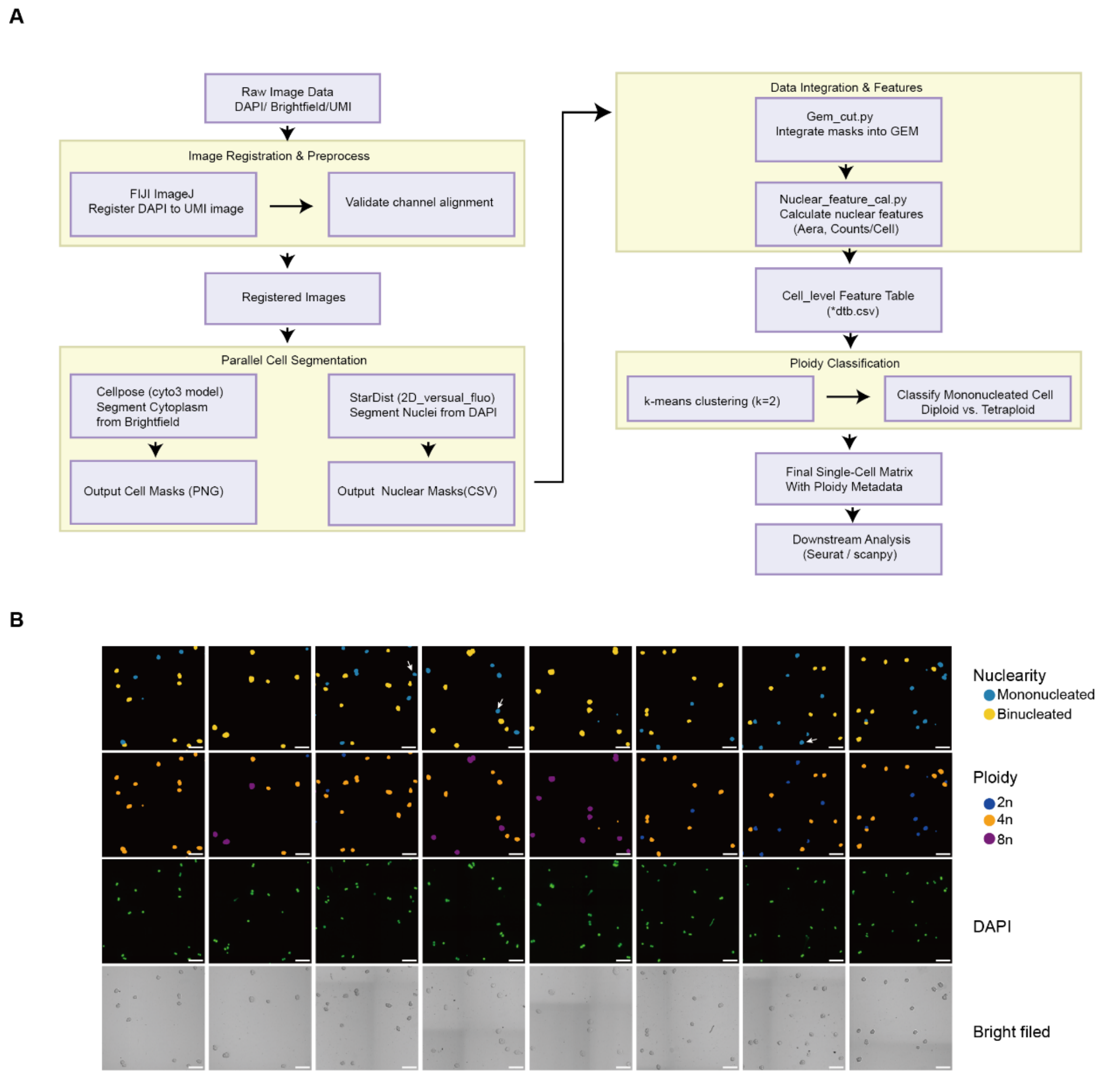

Figure 2: SCIPl pipeline for spatially resolved hepatocyte polyploidy profiling. (A) Workflow for identifying hepatocyte ploidy subtypes. (B) Representative field showing identification of hepatocyte ploidy subtypes. Arrows indicate misidentified cells. Scale bar: 100 μm.

SCIPI for classifying polyploid hepatocyte subpopulation

Polyploidization constitutes an essential biological feature of mammalian hepatocytes, with developmental programming driving nuclear DNA accumulation through both cytokinesis failure and nuclear duplication [8]. In hepatocytes, nuclear area is correlated with ploidy, as it increases with higher ploidy levels[15]. In our stained image, polyploid hepatocytes exhibit significantly larger nuclear areas compared to diploid nuclei (**Fig. 3A**). Based on the

distribution of nuclear areas, hepatocytes were classified into distinct ploidy classes (**Fig. 3B**): 2n × 1, 2n × 2, 4n × 1, and 4n × 2. The nuclear area of 4n × 2 hepatocytes is larger than that of 4n × 1, which in turn is larger than that of 2n × 1. Notably, 4n × 1 nuclei are derived from 2n × 2 cells through normal mitotic division, which explains why 2n × 2 and 4n × 1 nuclei exhibit comparable nuclear areas.

This evolutionary conserved mechanism establishes substantial polyploid populations, reaching ~90% cellular prevalence in rodents and ~50% in human livers under homeostatic conditions [10, 43-45]. In our data, quantitative analysis revealed polyploid hepatocytes constituting >75% of parenchymal cells in P56 mice **(Fig. 3C, D**). Among these ploidy types, the proportions were quantified as follows: approximately 20% were 2n × 1, over 30% each for 2n × 2 and 4n × 1, and about 10% were 4n × 2.

Fig. 3 Quantifying and Classifying Hepatocyte Polyploidy

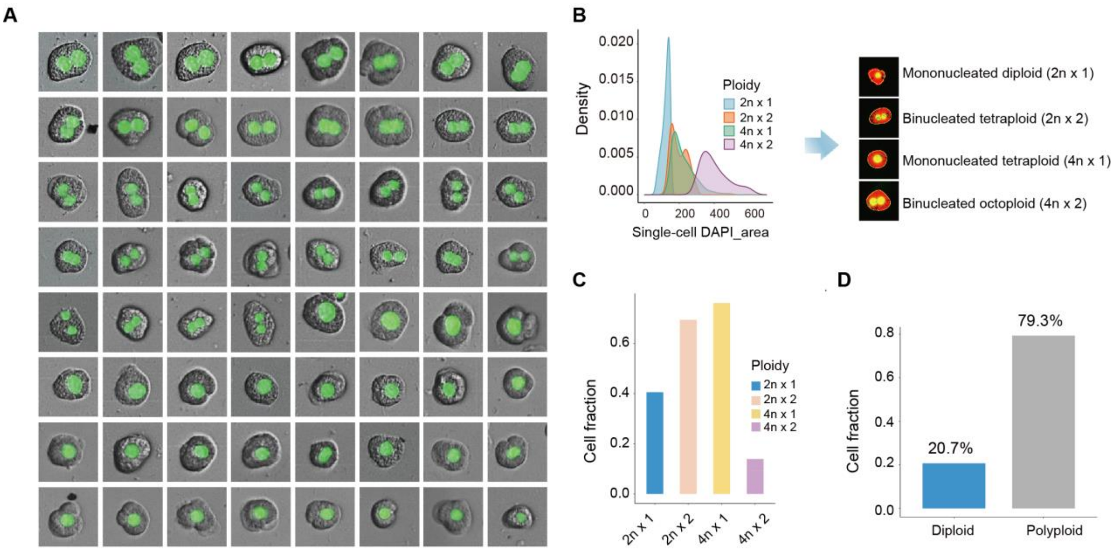

Figure 3: Quantifying and classifying hepatocyte polyploidy. (A) Representative hepatocytes with varying nuclear sizes, showing binucleated and mononucleated types. DAPI (green). (B) Quantification of hepatocyte nuclear area. (C) Proportions of the four hepatocyte ploidy subpopulations. (D) Proportion of diploid and polyploid Hepatocytes.

Polyploid hepatocyte distribution across liver lobule

To spatially resolve the heterogeneity of these polyploid populations, we applied our SCIPI

pipeline to generate a single-cell gene expression matrix augmented with ploidy. We resolved hepatocyte subpopulations into pericentral (PC) and periportal (PP) zonation identities based on the expression of established marker genes (*Cyp2e1* for pericentral, PC; *Cyp2f2* for periportal, PP)[46, 47] (**Fig. 4A**). By integrating these spatial transcriptomic and imaging-based classifications, we precisely mapped the lobular distributions of key hepatocyte subtypes (**Fig. 4B**). The lobular distribution of hepatocyte ploidy subpopulations identified by SCIPI were consistent with previous studies that employed nuclear and cell membrane staining on histological liver sections[15]. SCIPI analysis revealed a pronounced spatial patterning, with 4n × 1 and 4n × 2 hepatocytes enriched in the pericentral zone. A contrasting pattern was observed for 2n × 1 and 2n × 2 hepatocytes, which predominated in the periportal zone, suggesting a preferential enrichment of high-ploidy hepatocytes in the pericentral region.

To further investigate the expression dynamics of zonation-associated genes across different hepatocyte ploidy states, we selected established central vein (CV)-enriched genes (*Cyp2e1*, *Cyp1a2*, *Cyp27a1*) and periportal vein (PV)-enriched genes (*Cyp2f2*, *Gls2*, *Xbp1*) **(Fig. 4C-E**). The overall expression gradients of these genes from CV to PV were consistent with previously reported patterns. When these expression profiles were stratified into four hepatocyte ploidy subtypes, most genes exhibited generally conserved zonation trends across subtypes[28, 32, 46, 47]. However, a notable divergence was observed for *Xbp1*. While its expression increased progressively from CV to PV in the periportal 2n × 2 and 4n × 1 subtypes, it displayed a declining trend in the 2n × 1 and 4n × 2 subtypes.

Fig. 4 Gene Expression along CV to PV Trajectory by Ploidy

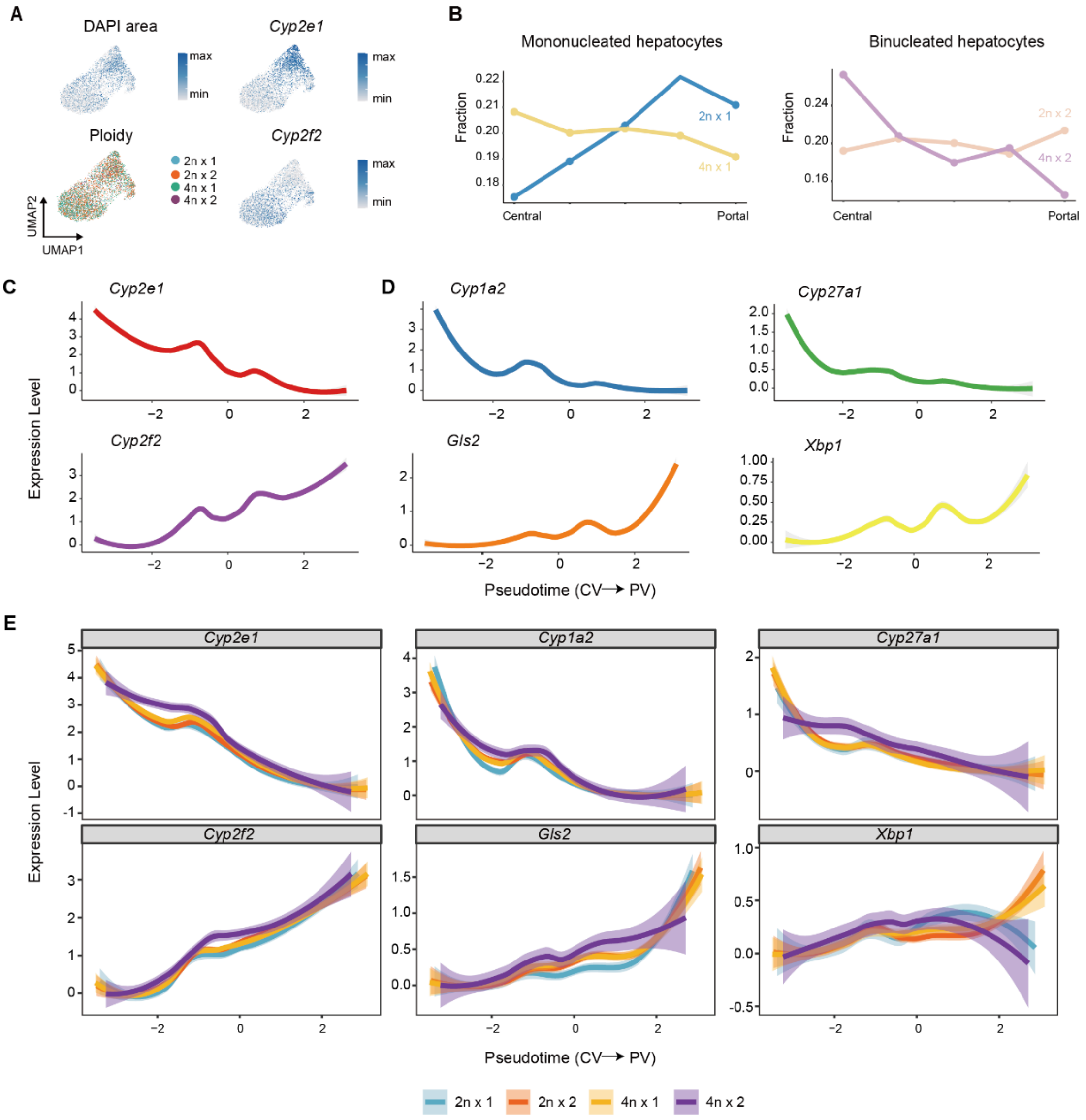

Figure 4: Gene expression along CV to PV trajectory by ploidy. (A) UMAP plots showing hepatocyte nuclear area (DAPI area), ploidy subpopulation distribution, and expression of *Cyp2e1* and *Cyp2f2*. (B) Zonal distribution of mononucleated and binucleated hepatocytes from central vein (CV) to portal vein (PV). (C-D) Zonation gene expression from CV to PV. (E) Expression of zonation genes across hepatocyte ploidy subtypes along the CV–PV axis.

The transcriptomic profiles analysis of polyploidy hepatocyte

To investigate whether gene expression increases correspondingly with genomic ploidy in hepatocytes, we compared and analyzed the transcriptomic differences among 2n × 2, 4n × 1, and 4n × 2 hepatocytes. Transcriptomic profiling further demonstrated that 2n × 2, 4n × 1 and

4n × 2 hepatocytes exhibited significantly elevated RNA molecule counts (RNA count) and gene numbers (RNA feature) compared to 2n × 1 counterparts (**Fig. 5A**). Notably, 4n × 2 subtypes also exhibited marked transcriptional differences relative to both 4n × 1 and 2n × 2 populations, and both 4n × 1 and 2n × 2 significantly more transcripts and 2n x 1. Differential gene expression analysis revealed ploidy-associated signatures, with a majority of expressed genes showing consistent upregulation patterns in 2n × 2, 4n × 1 and 4n x 2 hepatocytes relative to 2n × 1 hepatocytes (**Fig. 5B**).

To accurately assess gene expression differences among the four hepatocyte ploidy subpopulations, we analyzed a panel of liver-specific genes—including *Alb*, *Hnf4a*, and *Mup20*—as well as key genes involved in metabolic processes such as glucose metabolism (*Aldob*, *Gys2*, *Pygl*), urea synthesis (*Arg1*, *Ass1*, *Glud1*), lipid metabolism (*Apoa1*, *Apoc1*, *Apoh*), and xenobiotic metabolism (*Cyp2c23*, *Cyp2d26*, *Cyp2j5*) (**Fig. 4C-G**). Among the liver-specific markers, *Alb* and *Hnf4a* showed no significant differences in expression across diploid (2n × 1), binucleated diploid (2n × 2) and mononucleated tetraploid hepatocytes (4n × 1) but were significantly upregulated in binucleated octoploid (4n × 2) cells. In contrast, *Mup20* was markedly downregulated in both mononucleated tetraploid and binucleated octoploid populations (4n × 1 and 4n × 2), whereas *Mup3* expression remained stable across all ploidy types. For urea cycle-related genes, *Arg1*, *Ass1*, *Got1*, and *Otc* were significantly upregulated in binucleated tetraploid (2n × 2), mononucleated tetraploid (4n × 1), and binucleated octoploid (4n × 2) subpopulations. *Gpt* and *Asl* were elevated specifically in the 4n × 2 group, while *Glud1* was upregulated in both mononucleated tetraploid subtypes and the octoploid group.

Based on these expression profiles, we categorized the genes into four recurrent patterns (**Fig. 4H**): Type 1, upregulated exclusively in octoploid (4n × 2) hepatocytes; Type 2, elevated in both octoploid and mononucleated tetraploid cells; Type 3, upregulated across all tetraploid and octoploid subtypes; and Type 4, showing no notable variation among ploidy classes. Representative genes exemplifying these patterns in glucose metabolism included Type 1 (*Aldob*, *Pck1*, *Ugp2*), Type 2 (*Gys2*, *Pygl*, *Pcx*), Type 3 (*Fbp1*), and Type 4 (*G6pc*). In lipid metabolism, Type 1 included *Apoe*, *Mttp*, and *Apoa2*; Type 2 featured *Apoh*, *Acox1*, and *Apoc1*;

and Type 4 was represented by *Apob*. For xenobiotic metabolism, Type 1 genes encompassed *Cyp2d26*, *Cyp3a11*, and *Cyp2d9*; Type 2 included *Ces1d*, *Cyp1a2*, and *Cyp2e1*; Type 3 contained *Cyp2j5* and *Cyp2c23*; and Type 4 was represented by *Cyp7b1*. Most genes conformed to Type 1 or Type 2 patterns, with fewer following Type 3 or Type 4. Furthermore, deviating from the typical Type 2 pattern, a subset of genes such as *Cyp2f2* and *Apoc3* were specifically upregulated in octoploid and binucleated tetraploid hepatocytes, not in mononucleated tetraploid cells. This observation indicates that nuclear number, independent of overall ploidy, can modulate gene expression and implies a role for nucleo-cytoplasmic interactions in transcriptional regulation. The coordinated increase in the expression of these metabolic genes with ploidy suggests that hepatocytes with higher ploidy exhibit enhanced metabolic activity.

These results demonstrate that the increase in genomic DNA content in polyploid cells is accompanied by a corresponding elevation in transcriptional abundance. Collectively, our findings indicate that hepatocyte polyploidization significantly augments diverse metabolic functions within the liver.

Fig. 5 Transcriptomic Diversity Across Hepatocyte Ploidy Subpopulations

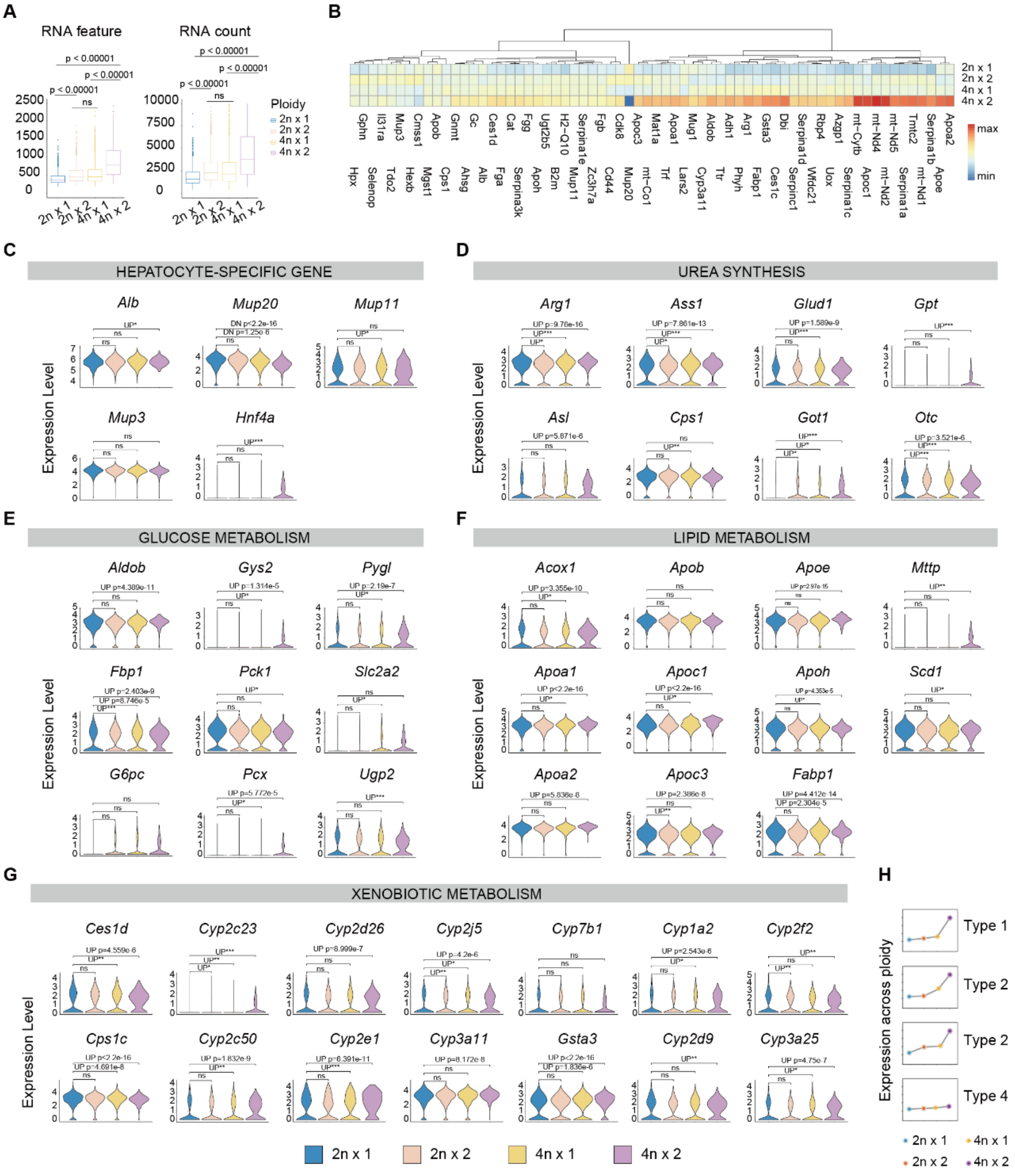

Figure 5: Transcriptomic diversity across hepatocyte ploidy subpopulations. (A) Transcript feature (RNA feature) and count (RNA count) across the four hepatocyte ploidy subpopulations. (B) Heatmap showing gene expression across the four hepatocyte ploidy subpopulations. (C) Violin plots of gene expression for key liver-specific genes across hepatocyte ploidy subpopulations. (D-G) Violin plots display gene expression across hepatocyte ploidy subpopulations for urea synthesis (D), glucose metabolism (E), lipid metabolism (F), and xenobiotic metabolism (G). (H) Schematic illustration of gene expression patterns across the four hepatocyte ploidy subtypes.

Discussion

We developed SCIPI technology that enables high-confidence identification of hepatocyte ploidy subtypes while achieving robust gene detection sensitivity. In this study, SCIPI constructed a comprehensive atlas of polyploid hepatocyte heterogeneity, achieving both a greater cell number and higher classification accuracy than conventional nuclear-sorting methods. Our results demonstrate that in P56 C57BL/6J male mice, polyploid hepatocytes account for over 75% of the total hepatocyte population, with the tetraploid forms (2n x 2 and 4n x 1) being the most abundant and present in comparable proportions. Spatially within the hepatic lobule, the proportion of 2n x 1 and 2n x 2 hepatocytes gradually decreases along the portal vein-to-central vein axis, whereas the proportion of 4n x 1 and 4n x 2 hepatocytes progressively increases. At the transcriptomic level, the number of genes and transcripts detected in polyploid hepatocytes was substantially higher than in diploid hepatocytes. Furthermore, polyploid hepatocytes exhibited enhanced expression of metabolism-related genes, suggesting they perform more active metabolic functions. Collectively, SCIPI provides a new method for further investigating the mechanisms of liver polyploidy in regeneration, development, and various liver diseases. This technical advancement also opens new avenues for investigating previously intractable polyploid cell populations, including cardiomyocytes[3, 48-50] and skeletal myofibers[51-53], which have historically resisted reliable single-cell trancriptomic analysis due to their complex nuclear architectures.

Compared to the FACS-based method for hepatocyte ploidy analysis (**Table 1**), our approach offers several critical advantages. First, it utilizes fresh, viable hepatocytes rather than isolated nuclei from frozen tissue, enabling the comprehensive capture of whole-cell transcriptomes. This intact-cell methodology is crucial for accurately discriminating binucleated polyploid subtypes (e.g., 2n × 2, 4n × 2), a capability lost in nuclear-enriched FACS approaches. Second, SCIPI consistently yields a greater number of high-quality cells per batch. For instance, a single chip in our P56 mouse dataset routinely provided ~2,500 hepatocytes after quality control, dramatically exceeding the ~400 hepatocytes typically obtainable through DNA-content-based FACS sorting of liver nuclei from P60 mice, as reported by Li et al[29].

Finally, the platform is highly scalable, efficiently processing cellular inputs ranging from minimal samples (~200 hepatocytes) to substantial populations (~5,000 hepatocytes), thereby generating high-quality, high-volume ploidy data.

SCIPI allows systematic detection of all polyploid hepatocyte subtypes. However, current limitations include inadequate adhesion of some hepatocytes to the chip surface, resulting in cell displacement or detachment during processing and consequently reducing both cell retention and RNA capture efficiency. Stereo chip’s encapsulation-free architecture, while conferring advantages over mainstream scRNA-seq methodologies, exhibits heightened susceptibility to lateral RNA diffusion. Although this effect remains minimal at optimal hepatocyte densities, it may nevertheless generate low-magnitude transcript leakage between adjacent hepatocytes. This limitation potentially addressable through future refinements in hepatocyte-specific permeabilization protocols and capture kinetics. Concurrently, ultra-low hepatocyte inputs compromise transcript detection efficiency due to diminished library complexity. Mitigation strategies such as sequencing depth optimization, background suppression techniques, multiplexed cellular barcoding, and implementation of spike-in controls. Furthermore, RNA capture efficiency for hepatocytes processed via SCIPI retains scope for improvement. These technical constraints will drive future refinements of the SCIPI for polyploid hepatocyte analysis. Collectively, our pilot studies demonstrate SCIPI's superior capacity for resolving hepatocyte polyploid subtypes compared to existing methodologies.

Table 1 | Comparison of different polyploid hepatocytes classifying methods										
	Category									
Method	Material	Treatment	Staining	Imaging	Classifying approach	Classifying condition	Ploidy subtypes	QC-passed cell yield/sample	Mean gene per cell	Sequencing
Co-staining[27]	Mouse P60 liver	Cryosection	DAPI, phalloidin	DAPI, FITC	Cryosection imaging	in situ	2nx1, 2nx2, 4nx1, 4nx2	~725	N.A.	N.A.

FACS snRNA-seq2[28]	Mouse P90 liver	Nuclear isolation	DAPI	N.A.	Nuclear DNA sorting	In dilute solution	2n, 4n	~401	~4000	snRNA-seq
FACS scRNA-seq[29]	Mouse P60 liver	Hepatocyte isolation	Hoechst 33342	N.A.	Whole-cell DNA sorting	In dilute solution	2n, 4n, 8n	~352	~6000	scRNA-seq
MERFISH[54]	Healthy human liver	Cryosection	DAPI, antibody stain	DAPI	Cryosection imaging	in situ	Single and multiple nuclei	~18000	~1000	scRNA-seq
SCIPI	Mouse P56 liver	Hepatocyte isolation	DAPI	Bright field, DAPI	Integrated spatial nuclear imaging and nuclear quantification	In dilute solution	2nx1, 2nx2, 4nx1, 4n x 2	~2500	~2800	scRNA-seq

Methods

Hepatocytes isolation and preparation

We implemented a modified two-step collagenase perfusion protocol[34-40], wherein 8-week-old C57BL/6J male mice livers were perfused via the inferior vena cava sequentially with EGTA-containing perfusion buffer followed by collagenase IV/$CaCl_2$ digestion buffer. The resultant homogenate underwent sequential filtration through 70 µm and 40 µm strainers to eliminate residual clusters, with microscopic examination confirming >96% viability prior to loading. Hepatocytes were resuspended in calcium-free 0.04% BSA in DPBS to prevent reaggregation. Immediately proceed to Stereo-cell following viability assessment to prevent hepatocyte death associated with prolonged storage (≤ 1 hr post-isolation at 4°C).

Stereo chip preprocessing for hepatocyte loading and microimaging

Following the Stereo-cell's guidelines[33] with modifications. A Stereo chip loaded in a 24-well plate was initially washed twice with nuclease-free water. To enhance cell adhesion, the

chip was transferred to a new well, coated with 400 μL of 0.01% poly-L-lysine solution, and incubated on an orbital shaker (50-250 rpm) at room temperature for one hour. After aspiration of the coating solution, the chip was rinsed twice with nuclease-free water and moved to a clean well. A suspension of 5,000 hepatocytes in 20 μL of DPBS containing 0.04% BSA was carefully applied to the chip surface. It is critical to gently spread the suspension with a pipette tip for even cell distribution while avoiding direct contact with the chip surface. The chip was incubated at room temperature for 15 minutes to allow cell attachment, followed by fixation in ice-cold methanol (-20 °C) for 30 minutes. Fixed cells are subsequently stained with DAPI solution, with systematic exclusion of glycerol in mounting protocols to prevent hydrodynamic displacement of large polyploid hepatocytes during high-resolution imaging. Final micrographs are acquired using a Motic PA53 FS6 microscope operating synchronized bright-field and DAPI fluorescence channels, ensuring co-registered.

Library construction and sequencing depth

The Stereo-cell library construction for hepatocytes refers to Liao et al in 2025[33]. After imaging, hepatocytes were permeabilized with 0.1% pepsin at 37°C for 30 seconds. RNA released from the permeabilized cells was captured by the chip's DNA Nanoballs (DNBs) and subsequently reverse transcribed overnight at 42°C. Chips containing cDNA were then treated overnight at 55°C for 3h with a cDNA release mixture. The released cDNA was collected and amplified using sequencing sample barcode primers to generate Stereo-cell libraries. These libraries were quantified using the Qubit dsDNA Assay Kit and either the Agilent 2100 Bioanalyzer or Qsep, respectively. We determined that a minimum of ~20 million paired-end reads per Stereo-cell library is sufficient for downstream analysis. Notably, the required sequencing depth should be determined based on the genome size of the species under investigation.

Raw sequencing data preprocessing

After sequencing, fastq format files will be obtained, which contain DNA base sequences, spatial coordinate sequences, and unique molecular identifier (UMI) sequences， after processing with SAW[41] (https://github.com/STOmics/SAW), we can obtain GEM-format files that have undergone sequence demultiplexing and alignment. This file contains Gene IDs, gene coordinates (x, y), molecular identifier count (MID Count), and exon counts. Based on this information, we can generate in situ UMI counts image (UMI image) with script gem2mask.py.

Image registration and preprocessing

Image registration was performed using FIJI ImageJ[55]. Since the microscope stage remained stationary during sequential imaging, the brightfield and DAPI images were inherently aligned. Thus, ImageJ was solely used to register the DAPI image with the corresponding UMI image. Both DAPI and brightfield images were converted from RGB to 8-bit to reduce memory usage, and brightness was adjusted to achieve consistency between the DAPI and UMI signals. A TrackEM2 project was created, and the folder containing the brightfield, DAPI, and UMI images was imported. The UMI image was set as the active layer and unlinked from other images to prevent unintended adjustments during registration, while the DAPI and brightfield images remained linked. The DAPI layer was then selected as the active layer, and channel assignments were configured with UMI as red and DAPI as green. The DAPI image was aligned to the UMI image using the Transform tool. Registration accuracy was verified by inspecting multiple fields of view to confirm overlap between the DAPI signal (green) and UMI regions (red). After validation, the aligned DAPI and brightfield images were exported as TIFF files.

Cell segmentation and ploidy analysis

Cellular segmentation was performed using a dual approach. Brightfield images were processed with Cellpose (cyto3 model; https://github.com/MouseLand/cellpose)[42], where cell diameter was estimated automatically using the calibration function. Resulting cell masks were saved as PNG files. Nuclei were segmented from registered DAPI images using StarDist (2D_versatile_fluo model; https://github.com/stardist/stardist)[56] via the StarDist_dapi.py

script, with output masks saved in CSV format. The Cellpose-derived segmentation masks were integrated into the GEM file using gem_cut.py, which adds a mask column assigning each cell a unique identifier. Nuclear-to-cellular correspondence was established using nuclear_feature_cal.py, where a DAPI mask was assigned to a cell if ≥50% of its area overlapped with the cellular region. Nuclear features—including mask associations, area, and counts per cell—were recorded in a DataFrame and saved as a "*dtb.csv" file. Ploidy classification was performed on mononucleated hepatocytes using k-means clustering (k=2) applied to nuclear area measurements. The threshold distinguishing diploid from tetraploid nuclei was defined as the minimum area within the higher-ploidy cluster, reflecting the discontinuous increase in nuclear size due to genome duplication. Ploidy assignment was implemented using the add_ploidy function in R_function.r.

A final single-cell matrix was generated incorporating ploidy annotations as metadata, enabling downstream analysis in Seurat or Scanpy.

Ethics approval

All relevant animal experimental procedures described in this study comply with ethical regulations for animal research and were performed under the approval of the Institute of Review Board of Bioethics and Biosafety of BGI (BGI-IRB), with the license number BGI-IRB A24013-T1.

Code availability

The SCIPI analysis pipeline is publicly accessible via GitHub: https://github.com/JeffLuo9/Hep_Ploidy_protocol.

Acknowledgements

We thank all team members of the Stereo-cell R&D group, with special acknowledgements to Xiaoxi Zhou, Hongyu Luo, Chang Liu, Yaling Huang, and Xuerong Li, for their robust support

and valuable technical assistance. We also thank Zhi Huang for his help with code development and optimization. All project analyses were performed using the STOmic Cloud platform (https://cloud.stomics.tech).

Author contributions

S.H. conceived and supervised this study, and designed the original analytical strategy for hepatocyte ploidy classification. Y.Y. implemented and optimized the Stereo-cell experimental protocol with assistance from J.L., H.W., and P.G. J.L. developed the computational pipeline and conducted most of the data analyses with assistance from Y.Y. Y.Y. and J.L. drafted the manuscript, with input from all authors.

Competing interests

The authors declare no competing interests.

Reference

[1] A.C. Rios, N.Y. Fu, P.R. Jamieson, B. Pal, L. Whitehead, K.R. Nicholas, G.J. Lindeman, J.E. Visvader, Essential role for a novel population of binucleated mammary epithelial cells in lactation, Nat Commun 7 (2016) 11400.
[2] Y. Unhavaithaya, T.L. Orr-Weaver, Polyploidization of glia in neural development links tissue growth to blood-brain barrier integrity, Genes Dev 26(1) (2012) 31-6.
[3] O.V. Anatskaya, A.E. Vinogradov, Genome multiplication as adaptation to tissue survival: evidence from gene expression in mammalian heart and liver, Genomics 89(1) (2007) 70-80.
[4] J. Zanet, A. Freije, M. Ruiz, V. Coulon, J.R. Sanz, J. Chiesa, A. Gandarillas, A mitosis block links active cell cycle with human epidermal differentiation and results in endoreplication, PLoS One

5(12) (2010) e15701.
[5] N. Sher, J.R. Von Stetina, G.W. Bell, S. Matsuura, K. Ravid, T.L. Orr-Weaver, Fundamental differences in endoreplication in mammals and Drosophila revealed by analysis of endocycling and endomitotic cells, Proc Natl Acad Sci U S A 110(23) (2013) 9368-73.
[6] O.V. Anatskaya, A.E. Vinogradov, B.N. Kudryavtsev, Hepatocyte polyploidy and metabolism/life-history traits: hypotheses testing, J Theor Biol 168(2) (1994) 191-9.
[7] G. Gentric, C. Desdouets, Liver polyploidy: Dr Jekyll or Mr Hide?, Oncotarget 6(11) (2015) 8430-1.
[8] M.J. Wang, F. Chen, J.T.Y. Lau, Y.P. Hu, Hepatocyte polyploidization and its association with pathophysiological processes, Cell Death Dis 8(5) (2017) e2805.
[9] G. Margall-Ducos, S. Celton-Morizur, D. Couton, O. Brégerie, C. Desdouets, Liver tetraploidization is controlled by a new process of incomplete cytokinesis, J Cell Sci 120(Pt 20) (2007) 3633-9.
[10] J. Fang, A. de Bruin, A. Villunger, R. Schiffelers, Z. Lei, J.P.G. Sluijter, Cellular polyploidy in organ homeostasis and regeneration, Protein Cell 14(8) (2023) 560-578.
[11] M. Fortier, S. Celton-Morizur, C. Desdouets, Incomplete cytokinesis/binucleation in mammals: The powerful system of hepatocytes, Methods Cell Biol 137 (2017) 119-142.
[12] R. Donne, M. Saroul-Aïnama, P. Cordier, S. Celton-Morizur, C. Desdouets, Polyploidy in liver development, homeostasis and disease, Nat Rev Gastroenterol Hepatol 17(7) (2020) 391-405.
[13] J.E. Guidotti, O. Brégerie, A. Robert, P. Debey, C. Brechot, C. Desdouets, Liver cell polyploidization: a pivotal role for binuclear hepatocytes, J Biol Chem 278(21) (2003) 19095-101.
[14] S. Celton-Morizur, G. Merlen, D. Couton, G. Margall-Ducos, C. Desdouets, The insulin/Akt pathway controls a specific cell division program that leads to generation of binucleated tetraploid liver cells in rodents, J Clin Invest 119(7) (2009) 1880-7.
[15] H.W. Chao, M. Doi, J.M. Fustin, H. Chen, K. Murase, Y. Maeda, H. Hayashi, R. Tanaka, M. Sugawa, N. Mizukuchi, Y. Yamaguchi, J.I. Yasunaga, M. Matsuoka, M. Sakai, M. Matsumoto, S. Hamada, H. Okamura, Circadian clock regulates hepatic polyploidy by modulating Mkp1-Erk1/2 signaling pathway, Nat Commun 8(1) (2017) 2238.
[16] S.K. Pandit, B. Westendorp, S. Nantasanti, E. van Liere, P.C. Tooten, P.W. Cornelissen, M.J. Toussaint, W.H. Lamers, A. de Bruin, E2F8 is essential for polyploidization in mammalian cells, Nat Cell Biol 14(11) (2012) 1181-91.
[17] H.Z. Chen, M.M. Ouseph, J. Li, T. Pécot, V. Chokshi, L. Kent, S. Bae, M. Byrne, C. Duran, G. Comstock, P. Trikha, M. Mair, S. Senapati, C.K. Martin, S. Gandhi, N. Wilson, B. Liu, Y.W. Huang, J.C. Thompson, S. Raman, S. Singh, M. Leone, R. Machiraju, K. Huang, X. Mo, S. Fernandez, I. Kalaszczynska, D.J. Wolgemuth, P. Sicinski, T. Huang, V. Jin, G. Leone, Canonical and atypical E2Fs regulate the mammalian endocycle, Nat Cell Biol 14(11) (2012) 1192-202.
[18] P.D. Wilkinson, E.R. Delgado, F. Alencastro, M.P. Leek, N. Roy, M.P. Weirich, E.C. Stahl, P.A. Otero, M.I. Chen, W.K. Brown, A.W. Duncan, The Polyploid State Restricts Hepatocyte Proliferation and Liver Regeneration in Mice, Hepatology 69(3) (2019) 1242-1258.
[19] M.J. Wang, F. Chen, J.X. Li, C.C. Liu, H.B. Zhang, Y. Xia, B. Yu, P. You, D. Xiang, L. Lu, H. Yao, U. Borjigin, G.S. Yang, K.J. Wangensteen, Z.Y. He, X. Wang, Y.P. Hu, Reversal of hepatocyte senescence after continuous in vivo cell proliferation, Hepatology 60(1) (2014) 349-61.
[20] G.R. Gorla, H. Malhi, S. Gupta, Polyploidy associated with oxidative injury attenuates proliferative potential of cells, J Cell Sci 114(Pt 16) (2001) 2943-51.

[21] H. Malhi, G.R. Gorla, A.N. Irani, P. Annamaneni, S. Gupta, Cell transplantation after oxidative hepatic preconditioning with radiation and ischemia-reperfusion leads to extensive liver repopulation, Proc Natl Acad Sci U S A 99(20) (2002) 13114-9.
[22] G. Gentric, V. Maillet, V. Paradis, D. Couton, A. L'Hermitte, G. Panasyuk, B. Fromenty, S. Celton-Morizur, C. Desdouets, Oxidative stress promotes pathologic polyploidization in nonalcoholic fatty liver disease, J Clin Invest 125(3) (2015) 981-92.
[23] K. Yin, M. Büttner, I.K. Deligiannis, M. Strzelecki, L. Zhang, C. Talavera-López, F. Theis, D.T. Odom, C.P. Martinez-Jimenez, Polyploidisation pleiotropically buffers ageing in hepatocytes, J Hepatol 81(2) (2024) 289-302.
[24] S. Zhang, K. Zhou, X. Luo, L. Li, H.C. Tu, A. Sehgal, L.H. Nguyen, Y. Zhang, P. Gopal, B.D. Tarlow, D.J. Siegwart, H. Zhu, The Polyploid State Plays a Tumor-Suppressive Role in the Liver, Dev Cell 44(4) (2018) 447-459.e5.
[25] W. Bu, X. Sun, X. Xue, S. Geng, T. Yang, J. Zhang, Y. Li, C. Feng, Q. Liu, X. Zhang, P. Li, Z. Liu, Y. Shi, C. Shao, Early onset of pathological polyploidization and cellular senescence in hepatocytes lacking RAD51 creates a pro-fibrotic and pro-tumorigenic inflammatory microenvironment, Hepatology 81(2) (2025) 491-508.
[26] J. Wang, X. Huang, D. Zheng, Q. Li, M. Mei, S. Bao, PRMT5 determines the pattern of polyploidization and prevents liver from cirrhosis and carcinogenesis, J Genet Genomics 50(2) (2023) 87-98.
[27] S. Tanami, S. Ben-Moshe, A. Elkayam, A. Mayo, K. Bahar Halpern, S. Itzkovitz, Dynamic zonation of liver polyploidy, Cell Tissue Res 368(2) (2017) 405-410.
[28] M.L. Richter, I.K. Deligiannis, K. Yin, A. Danese, E. Lleshi, P. Coupland, C.A. Vallejos, K.P. Matchett, N.C. Henderson, M. Colome-Tatche, C.P. Martinez-Jimenez, Single-nucleus RNA-seq2 reveals functional crosstalk between liver zonation and ploidy, Nat Commun 12(1) (2021) 4264.
[29] L. Yang, X. Wang, J.X. Zheng, Z.R. Xu, L.C. Li, Y.L. Xiong, B.C. Zhou, J. Gao, C.R. Xu, Determination of key events in mouse hepatocyte maturation at the single-cell level, Dev Cell 58(19) (2023) 1996-2010.e6.
[30] X. Wei, S. Fu, H. Li, Y. Liu, S. Wang, W. Feng, Y. Yang, X. Liu, Y.Y. Zeng, M. Cheng, Y. Lai, X. Qiu, L. Wu, N. Zhang, Y. Jiang, J. Xu, X. Su, C. Peng, L. Han, W.P. Lou, C. Liu, Y. Yuan, K. Ma, T. Yang, X. Pan, S. Gao, A. Chen, M.A. Esteban, H. Yang, J. Wang, G. Fan, L. Liu, L. Chen, X. Xu, J.F. Fei, Y. Gu, Single-cell Stereo-seq reveals induced progenitor cells involved in axolotl brain regeneration, Science 377(6610) (2022) eabp9444.
[31] A. Chen, S. Liao, M. Cheng, K. Ma, L. Wu, Y. Lai, X. Qiu, J. Yang, J. Xu, S. Hao, X. Wang, H. Lu, X. Chen, X. Liu, X. Huang, Z. Li, Y. Hong, Y. Jiang, J. Peng, S. Liu, M. Shen, C. Liu, Q. Li, Y. Yuan, X. Wei, H. Zheng, W. Feng, Z. Wang, Y. Liu, Z. Wang, Y. Yang, H. Xiang, L. Han, B. Qin, P. Guo, G. Lai, P. Muñoz-Cánoves, P.H. Maxwell, J.P. Thiery, Q.F. Wu, F. Zhao, B. Chen, M. Li, X. Dai, S. Wang, H. Kuang, J. Hui, L. Wang, J.F. Fei, O. Wang, X. Wei, H. Lu, B. Wang, S. Liu, Y. Gu, M. Ni, W. Zhang, F. Mu, Y. Yin, H. Yang, M. Lisby, R.J. Cornall, J. Mulder, M. Uhlén, M.A. Esteban, Y. Li, L. Liu, X. Xu, J. Wang, Spatiotemporal transcriptomic atlas of mouse organogenesis using DNA nanoball-patterned arrays, Cell 185(10) (2022) 1777-1792.e21.
[32] J. Xu, P. Guo, S. Hao, S. Shangguan, Q. Shi, G. Volpe, K. Huang, J. Zuo, J. An, Y. Yuan, M. Cheng, Q. Deng, X. Zhang, G. Lai, H. Nan, B. Wu, X. Shentu, L. Wu, X. Wei, Y. Jiang, X. Huang, F. Pan, Y. Song, R. Li, Z. Wang, C. Liu, S. Liu, Y. Li, T. Yang, Z. Xu, W. Du, L. Li, T. Ahmed, K. You, Z. Dai, L. Li, B. Qin, Y. Li, L. Lai, D. Qin, J. Chen, R. Fan, Y. Li, J. Hou, M. Ott, A.D. Sharma, T. Cantz, A. Schambach,

K. Kristiansen, A.P. Hutchins, B. Göttgens, P.H. Maxwell, L. Hui, X. Xu, L. Liu, A. Chen, Y. Lai, M.A. Esteban, A spatiotemporal atlas of mouse liver homeostasis and regeneration, Nat Genet 56(5) (2024) 953-969.

[33] S. Liao, X. Zhou, C. Liu, C. Liu, S. Hao, H. Luo, H. Hou, Q. Liu, Z. Zhang, L. Xiao, Y. Xu, Y. Huang, S. Zhou, X. Li, Y. Wang, L. Xie, Z. Zhou, S. Dong, Y. Wang, X. Xu, P. Guo, X. Lin, J. Lei, Q. Wang, Y. Gong, J. Cheng, Z. Yuan, Y. Yang, Z. Huang, S. Li, Y. Zheng, S. Yang, X. Huang, W. Liu, M. Li, Z. Deng, X. Yang, J. Yin, Y. Luo, Y. Lai, Y. Yuan, M. Cheng, B. Wang, J. Ji, M.A. Esteban, Y. Li, Y. Gu, Y. Ruan, L. Chen, X. Wang, J. Xie, J. Wang, L. Liu, A. Chen, X. Xu, Stereo-cell: Spatial enhanced-resolution single-cell sequencing with high-density DNA nanoball-patterned arrays, Science 389(6762) (2025).

[34] M.N. Berry, D.S. Friend, High-yield preparation of isolated rat liver parenchymal cells: a biochemical and fine structural study, J Cell Biol 43(3) (1969) 506-20.

[35] S.A. MacParland, J.C. Liu, X.Z. Ma, B.T. Innes, A.M. Bartczak, B.K. Gage, J. Manuel, N. Khuu, J. Echeverri, I. Linares, R. Gupta, M.L. Cheng, L.Y. Liu, D. Camat, S.W. Chung, R.K. Seliga, Z. Shao, E. Lee, S. Ogawa, M. Ogawa, M.D. Wilson, J.E. Fish, M. Selzner, A. Ghanekar, D. Grant, P. Greig, G. Sapisochin, N. Selzner, N. Winegarden, O. Adeyi, G. Keller, G.D. Bader, I.D. McGilvray, Single cell RNA sequencing of human liver reveals distinct intrahepatic macrophage populations, Nat Commun 9(1) (2018) 4383.

[36] D.A. Casciano, Development and utilization of primary hepatocyte culture systems to evaluate metabolism, DNA binding, and DNA repair of xenobiotics, Drug Metab Rev 32(1) (2000) 1-13.

[37] J.E. Klaunig, P.J. Goldblatt, D.E. Hinton, M.M. Lipsky, J. Chacko, B.F. Trump, Mouse liver cell culture. I. Hepatocyte isolation, In Vitro 17(10) (1981) 913-25.

[38] W.C. Li, K.L. Ralphs, D. Tosh, Isolation and culture of adult mouse hepatocytes, Methods Mol Biol 633 (2010) 185-96.

[39] M. Severgnini, J. Sherman, A. Sehgal, N.K. Jayaprakash, J. Aubin, G. Wang, L. Zhang, C.G. Peng, K. Yucius, J. Butler, K. Fitzgerald, A rapid two-step method for isolation of functional primary mouse hepatocytes: cell characterization and asialoglycoprotein receptor based assay development, Cytotechnology 64(2) (2012) 187-95.

[40] M. Charni-Natan, I. Goldstein, Protocol for Primary Mouse Hepatocyte Isolation, STAR Protoc 1(2) (2020) 100086.

[41] C. Gong, S. Li, L. Wang, F. Zhao, S. Fang, D. Yuan, Z. Zhao, Q. He, M. Li, W. Liu, Z. Li, H. Xie, S. Liao, A. Chen, Y. Zhang, Y. Li, X. Xu, SAW: an efficient and accurate data analysis workflow for Stereo-seq spatial transcriptomics, GigaByte 2024 (2024) gigabyte111.

[42] C. Stringer, T. Wang, M. Michaelos, M. Pachitariu, Cellpose: a generalist algorithm for cellular segmentation, Nat Methods 18(1) (2021) 100-106.

[43] G. Saeter, C.Z. Lee, P.E. Schwarze, S. Ous, D.S. Chen, J.L. Sung, P.O. Seglen, Changes in ploidy distributions in human liver carcinogenesis, J Natl Cancer Inst 80(18) (1988) 1480-5.

[44] A.W. Duncan, M.H. Taylor, R.D. Hickey, A.E. Hanlon Newell, M.L. Lenzi, S.B. Olson, M.J. Finegold, M. Grompe, The ploidy conveyor of mature hepatocytes as a source of genetic variation, Nature 467(7316) (2010) 707-10.

[45] A.W. Duncan, A.E. Hanlon Newell, L. Smith, E.M. Wilson, S.B. Olson, M.J. Thayer, S.C. Strom, M. Grompe, Frequent aneuploidy among normal human hepatocytes, Gastroenterology 142(1) (2012) 25-8.

[46] K.B. Halpern, R. Shenhav, O. Matcovitch-Natan, B. Toth, D. Lemze, M. Golan, E.E. Massasa, S.

Baydatch, S. Landen, A.E. Moor, A. Brandis, A. Giladi, A.S. Avihail, E. David, I. Amit, S. Itzkovitz, Single-cell spatial reconstruction reveals global division of labour in the mammalian liver, Nature 542(7641) (2017) 352-356.
[47] S. Ben-Moshe, S. Itzkovitz, Spatial heterogeneity in the mammalian liver, Nat Rev Gastroenterol Hepatol 16(7) (2019) 395-410.
[48] J.G. Bensley, R. De Matteo, R. Harding, M.J. Black, Three-dimensional direct measurement of cardiomyocyte volume, nuclearity, and ploidy in thick histological sections, Sci Rep 6 (2016) 23756.
[49] K.H. Østergaard, U.T. Baandrup, T. Wang, M.F. Bertelsen, J.B. Andersen, M. Smerup, J.R. Nyengaard, Left ventricular morphology of the giraffe heart examined by stereological methods, Anat Rec (Hoboken) 296(4) (2013) 611-21.
[50] K. Hirose, A.Y. Payumo, S. Cutie, A. Hoang, H. Zhang, R. Guyot, D. Lunn, R.B. Bigley, H. Yu, J. Wang, M. Smith, E. Gillett, S.E. Muroy, T. Schmid, E. Wilson, K.A. Field, D.M. Reeder, M. Maden, M.M. Yartsev, M.J. Wolfgang, F. Grützner, T.S. Scanlan, L.I. Szweda, R. Buffenstein, G. Hu, F. Flamant, J.E. Olgin, G.N. Huang, Evidence for hormonal control of heart regenerative capacity during endothermy acquisition, Science 364(6436) (2019) 184-188.
[51] D.P. Millay, Regulation of the myoblast fusion reaction for muscle development, regeneration, and adaptations, Exp Cell Res 415(2) (2022) 113134.
[52] M.J. Petrany, C.O. Swoboda, C. Sun, K. Chetal, X. Chen, M.T. Weirauch, N. Salomonis, D.P. Millay, Single-nucleus RNA-seq identifies transcriptional heterogeneity in multinucleated skeletal myofibers, Nat Commun 11(1) (2020) 6374.
[53] C. Sun, C.O. Swoboda, F.M. Morales, C. Calvo, M.J. Petrany, S. Parameswaran, A. VonHandorf, M.T. Weirauch, C. Lepper, D.P. Millay, Lineage tracing of nuclei in skeletal myofibers uncovers distinct transcripts and interplay between myonuclear populations, Nat Commun 15(1) (2024) 9372.
[54] B.R. Watson, B. Paul, R.U. Rahman, L. Amir-Zilberstein, Å. Segerstolpe, E.T. Epstein, S. Murphy, L. Geistlinger, T. Lee, A. Shih, J. Deguine, R.J. Xavier, J.R. Moffitt, A.C. Mullen, Spatial transcriptomics of healthy and fibrotic human liver at single-cell resolution, Nat Commun 16(1) (2025) 319.
[55] J. Schindelin, I. Arganda-Carreras, E. Frise, V. Kaynig, M. Longair, T. Pietzsch, S. Preibisch, C. Rueden, S. Saalfeld, B. Schmid, J.Y. Tinevez, D.J. White, V. Hartenstein, K. Eliceiri, P. Tomancak, A. Cardona, Fiji: an open-source platform for biological-image analysis, Nat Methods 9(7) (2012) 676-82.
[56] U. Schmidt, M. Weigert, C. Broaddus, G. Myers, Cell Detection with Star-Convex Polygons, Springer International Publishing, Cham, 2018, pp. 265-273.

Figure 1

Click here to access/download;Manuscript;GS_Fig1.pdf

Fig. 1 Comprehensive Stereo-cell Experimental Workflow for Hepatocytes

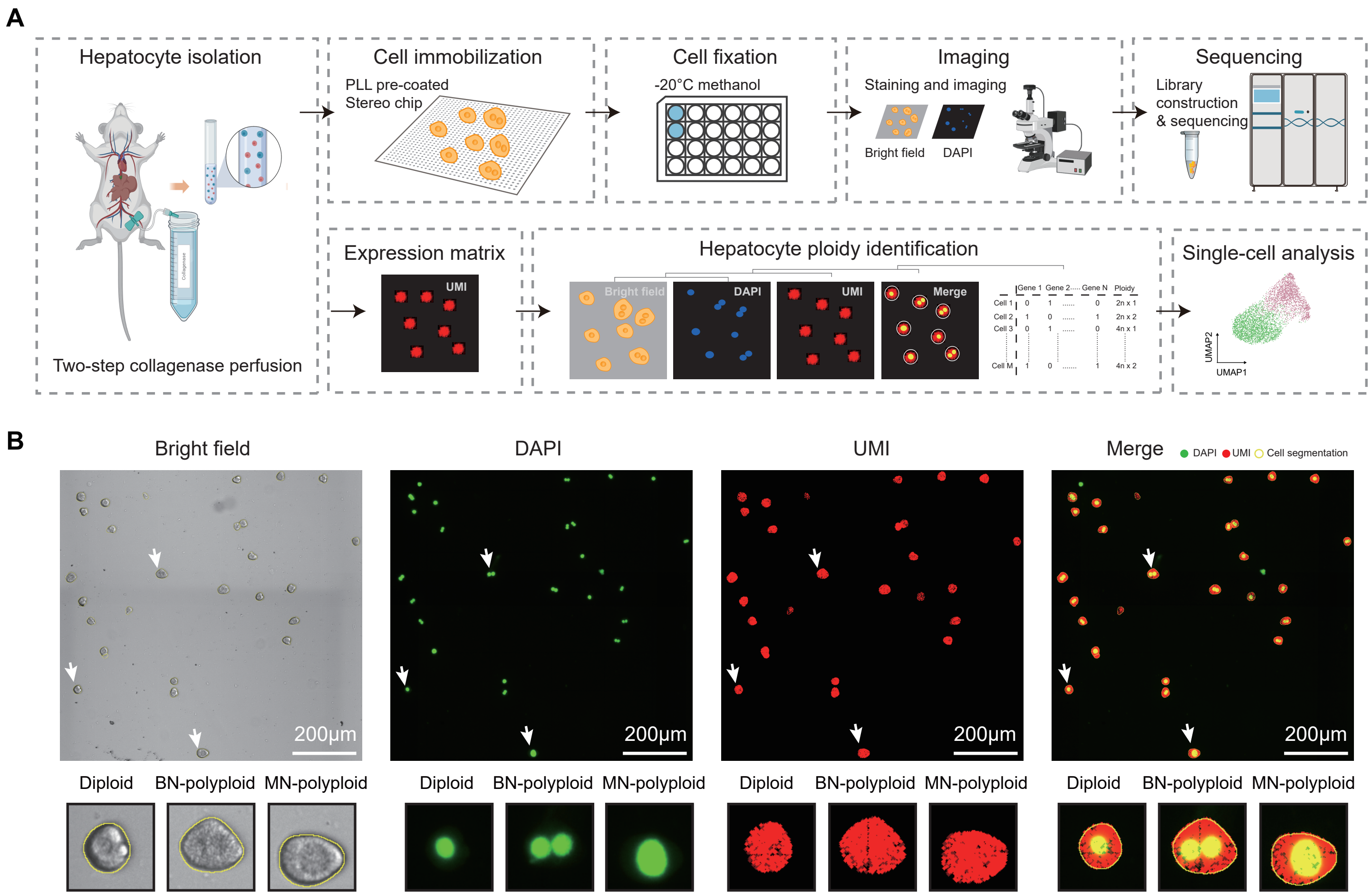

Click here to access/download;Manuscript;GS_fig2.pdf

Fig. 2 SCIPI pipeline for spatially resolved hepatocyte polyploidy profiling

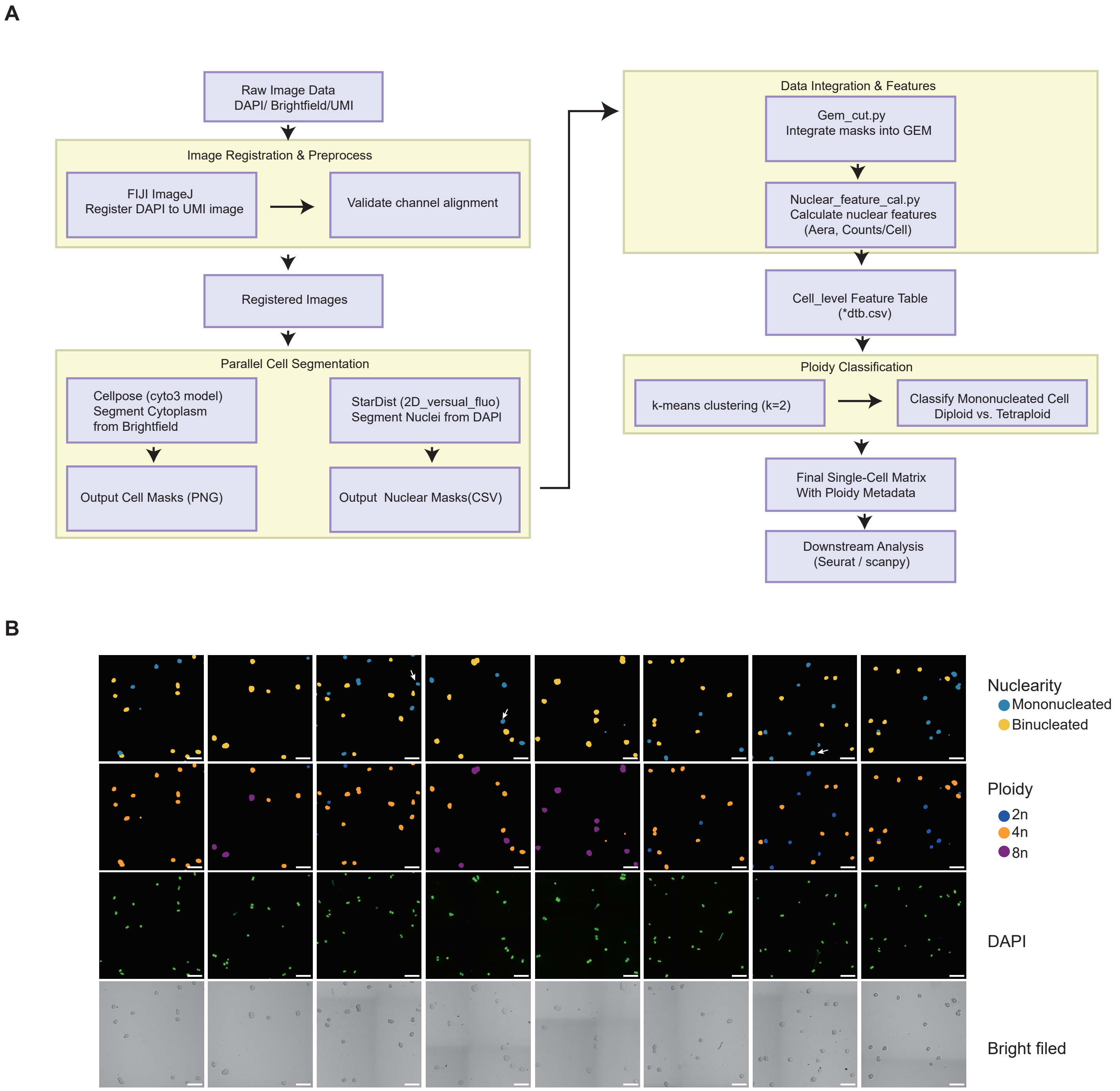

Figure 3

Click here to access/download;Manuscript;GS_fig3.pdf

Fig. 3 Quantifying and Classifying Hepatocyte Polyploidy

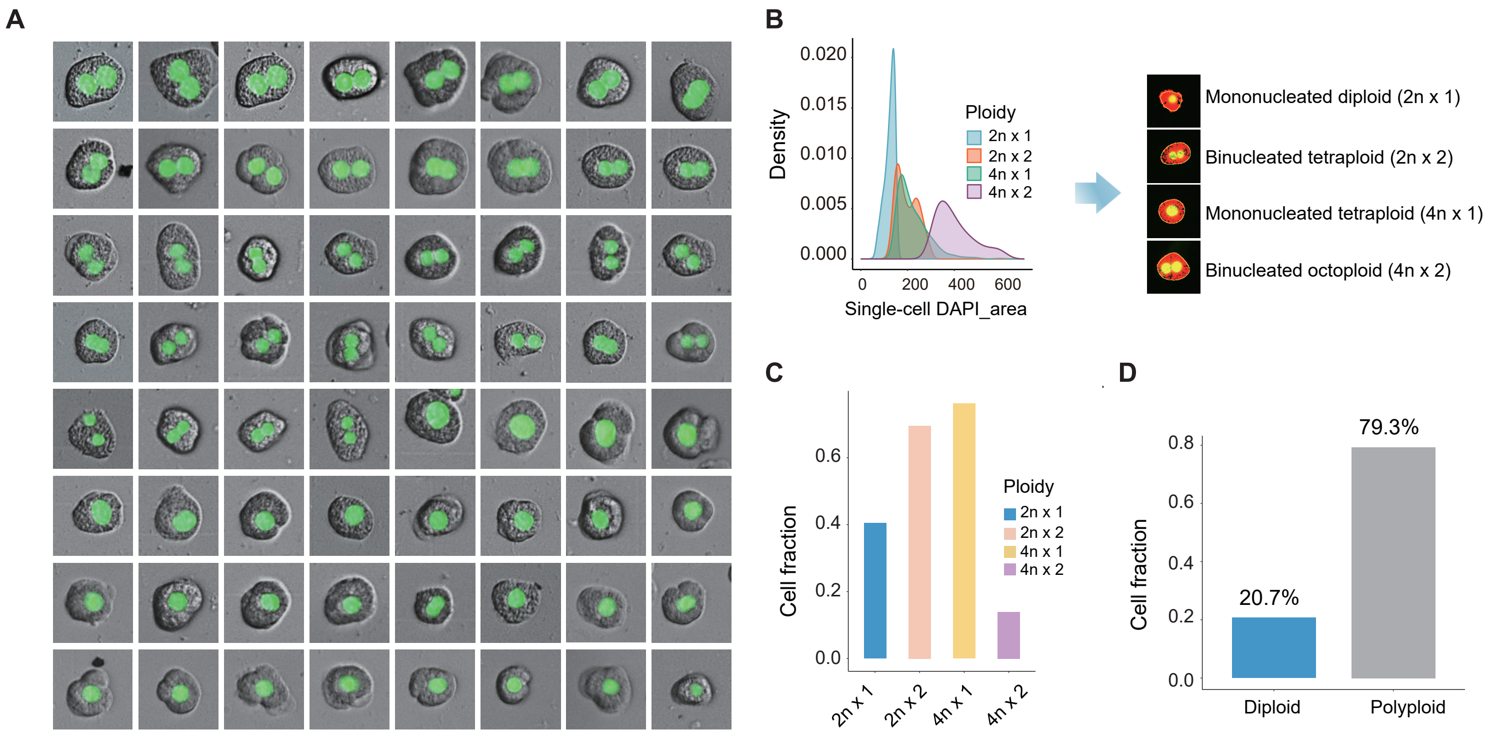

 Click here to access/download;Manuscript;GS_fig4.pdf

Fig. 4 Gene Expression along CV to PV Trajectory by Ploidy

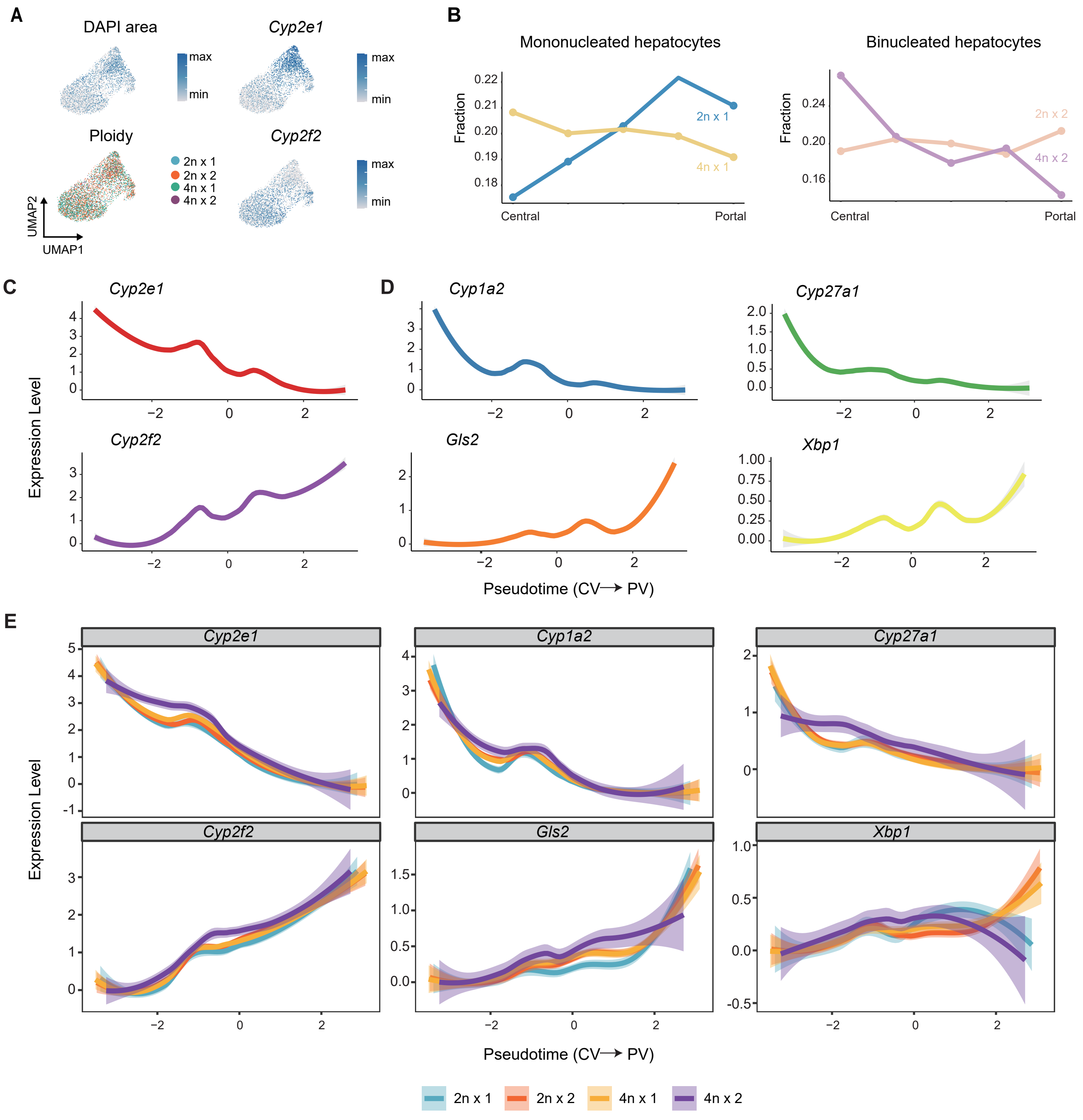

Fig. 5 Transcriptomic Diversity Across Hepatocyte Ploidy Subpopulations

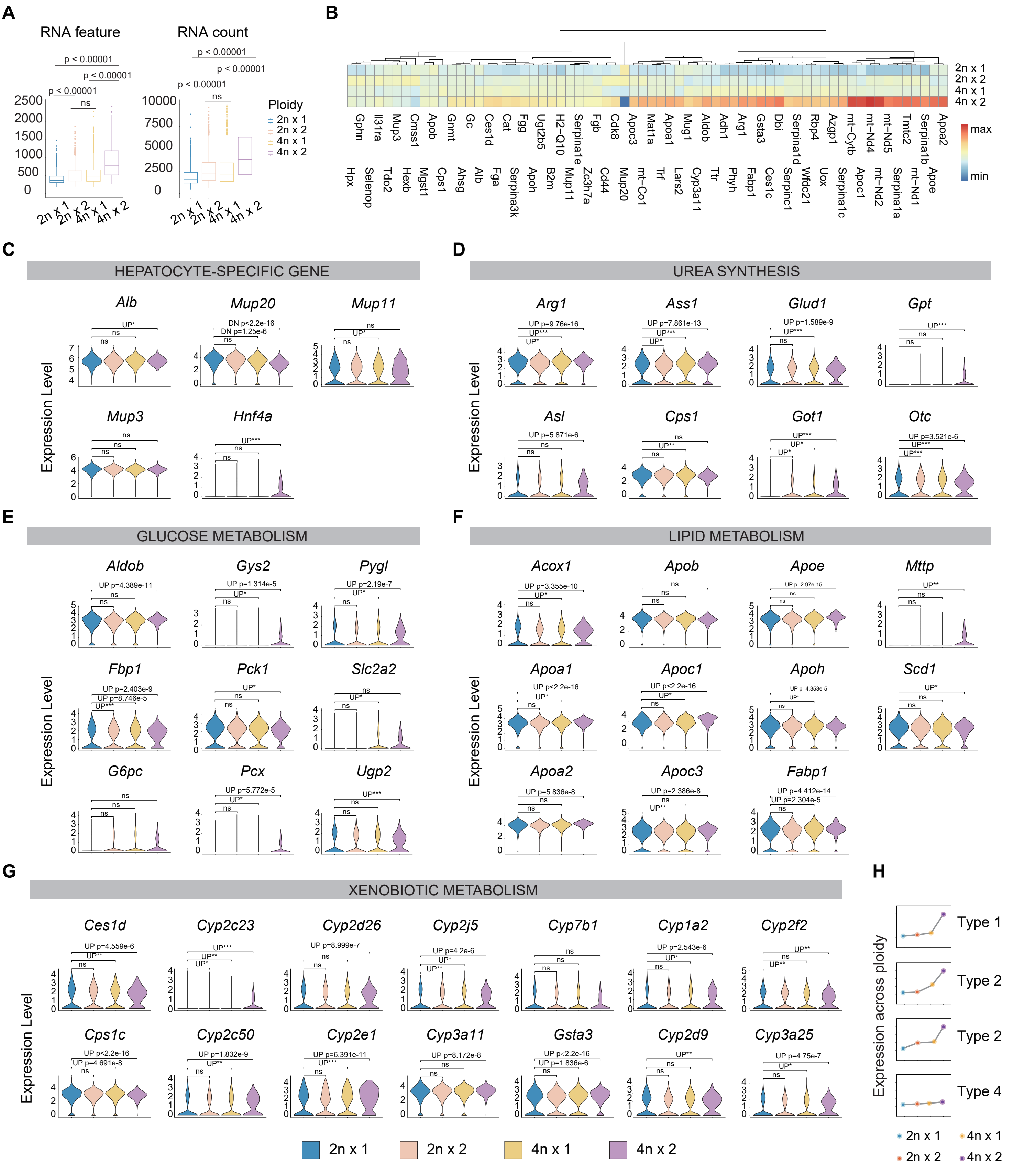

Supplement: giag023_GIGA-D-25-00452_original_submission [file giag023_giga-d-25-00452_original_submission.pdf]
