# Supplementary material for: Stereo-cell deciphers the spatial and functional heterogeneity of polyploid hepatocytes
Source: Gigascience. 2026 Mar 2;15:giag023. doi: 10.1093/gigascience/giag023 (PMC13100898; doi:10.1093/gigascience/giag023)
Supplement: giag023_GIGA-D-25-00452_Revision_1 [file giag023_giga-d-25-00452_revision_1.pdf]

GigaScience

Stereo-cell deciphers the spatial and functional heterogeneity of polyploid hepatocytes

--Manuscript Draft--

**Manuscript Number:**	GIGA-D-25-00452R1	
**Full Title:**	Stereo-cell deciphers the spatial and functional heterogeneity of polyploid hepatocytes	
**Article Type:**	Technical Note	
**Funding Information:**	Zhejiang Provincial Natural Science Foundation of China (LMS26C060001)	Dr Shijie Hao
	National Natural Science Foundation of China (32500590)	Dr Shijie Hao
	National Postdoctoral Program for Innovative Talents (BX20250145)	Dr Shijie Hao
**Abstract:**	A characteristic feature of the liver is the presence of numerous polyploid hepatocytes. However, the functional distinctions among diploid, tetraploid, and octoploid hepatocytes remain poorly understood. In this study, we employed the spatially resolved single-cell sequencing technology, Stereo-cell, to dissect the transcriptomic and functional heterogeneity across hepatocyte ploidy subtypes. We detail the development of Stereo-cell Imaging-based ploidy Identification (SCIPI), a technical pipeline that integrates bright-field cell contour recognition, DAPI-based nuclear area and number quantification, and UMI-barcoded single-cell transcriptomics. This approach enables precise identification of six core hepatocyte subtypes: mononucleated diploid (2n×1), mononucleated tetraploid (4n×1), binucleated tetraploid (2n×2), mononucleated octoploid (8n×1), binucleated octoploid (4n×2), and binucleated hexadecaploid (8n×2) hepatocytes. Single-cell transcriptomic analysis based on ploidy annotation revealed that gene expression levels scale positively with increasing ploidy and nuclear number. Metabolic pathway-associated genes were significantly upregulated in polyploid cells, suggesting that cellular polyploidization enhances the metabolic activity of hepatocytes. Furthermore, this SCIPI strategy is broadly applicable to the study of various polyploid tissues, offering a novel and versatile framework for innovative ploidy-resolved research across diverse biological researches.	
**Corresponding Author:**	Shijie Hao<br>BGI Group<br>Shenzhen, --- Select a state --- CHINA	
**Corresponding Author Secondary Information:**		
**Corresponding Author's Institution:**	BGI Group	
**Corresponding Author's Secondary Institution:**		
**First Author:**	Shijie Hao	
**First Author Secondary Information:**		
**Order of Authors:**	Shijie Hao	
	Yongqing Yang	
	Jiahui Luo	
	Hong Wu	
	Pengcheng Guo	
	Yier Cai	
	Qiang Guo	

Powered by Editorial Manager® and ProduXion Manager® from Aries Systems Corporation

	Longqi Liu
**Order of Authors Secondary Information:**	
**Response to Reviewers:**	Point-by-point response to the reviewers’ comments<br><br>We sincerely thank the reviewers for their thoughtful comments and constructive feedback on our work. We particularly appreciate their valuable suggestions regarding the technical details of the SCIPI platform, the depth of data analysis, and the exploration of biological mechanisms. These comments have been instrumental in helping us further improve the quality of our manuscript and enhance the rigor and scientific value of the study. In response, we have carefully addressed each point raised by the reviewers through detailed explanations, additional experiments, and deeper analyses.<br><br>Reviewers' comments<br><br>Reviewer #1: In this study, the authors developed a specific Sterreo-Cell-based technique to analyze cell ploidy and transcriptomics at the same time. The so-called SCIPI platform owns possible advantages in comparison to other conventional techniques and warrants further application in delineating physiological and pathological condistions. I have a few minor comments.<br><br>We greatly appreciate the multiple suggestions you provided for this manuscript. We have revised various problematic or insufficient parts in light of your comments, which has significantly improved the quality of the article. Below are the specific revisions we have made; please kindly review them again.<br><br>1. The capture efficiency in this chip was not specifically presented. The results showed different proportion for each ploidy status. Is this comparable to the real situation in mouse liver? I s here any capture bias for specific hepatocyte ploidy populations?<br><br>Our reply: This is a crucial question concerning potential capture bias of ploidy subtypes by the SCIPI technology. To address this, we performed fluorescence-activated cell sorting (FACS) based on DAPI fluorescence intensity (DNA content). The sorted populations consisted of 15.5% diploid (2c), 59.6% tetraploid (4c), 22.7% octoploid (8c), and 2.1% hexadecaploid (16c) hepatocytes (see below Rebuttal Fig. 1A). The corresponding FACS results have been added to the revised manuscript.<br><br>In parallel, the proportions of hepatocytes captured by the Stereo-cell-based SCIPI technique were 11.4% diploid, 66.2% tetraploid, 21.2% octoploid, and 1.8% hexadecaploid (see below Rebuttal Fig. 1B). The ploidy distribution obtained by SCIPI shows high concordance with that determined by FACS. Furthermore, no significant difference was observed between the ploidy proportions identified by SCIPI and those derived from liver tissue section analysis (see below Rebuttal Fig. 1C). Taken together, we conclude that the differences in subtype proportions between the SCIPI and FACS methods fall within an acceptable range of technical variation. Therefore, the SCIPI technique does not exhibit systematic capture bias against specific hepatocyte ploidy subtypes.<br><br><br>Rebuttal Fig. 1<br>(A) Proportions of the hepatocyte ploidy subpopulations as identified by SCIPI. (B) Flow cytometric DNA content analysis (DAPI staining) of hepatocytes, showing diploid (2c, 15.5%), tetraploid (4c, 59.6%), octoploid (8c, 22.7%) and hexadecaploid (16c, 2.1%) cells. (C) Hepatocyte ploidy fractions (diploid vs. polyploid) from SCIPI (red) and liver section analysis (green). Two-sided unpaired Student’ s t-test; ns, no significance.<br><br>2. As shown by the authors, the mean genes detected per cell in the developed platform was fairly adequate, but remained substantially lower than the FACS-based single-cell RNA profiling strategy. This may represent a limitation for the current pipeline, particularly for analysis of more complicatied disease contexts. This should be furtherdiscussed.

Our reply: Regarding gene detection sensitivity, we acknowledge that our current SCIPI approach yields a lower gene detection rate compared to FACS-based single-cell RNA profiling strategies, which we anticipate will be significantly improved through future technological upgrades. However, compared to FACS-sorted scRNA-seq (~350 cells; DOI: 10.1016/j.devcel.2023.07.006), our method captured a substantially larger number of qualified cells (~7,700) and achieved higher ploidy subtype classification accuracy. This point is now discussed in the revised manuscript.

Furthermore, to demonstrate the functional utility of our data despite the lower per-cell gene count, we successfully integrated our dataset with the published scRNA-seq data of homeostasis adult mouse liver (DOI: 10.1038/s41588-024-01709-7). The integrated analysis shows excellent alignment among the datasets (see below Rebuttal Fig. 2), indicating the data quality remains sufficient for robust downstream omics analyses, including clustering, integration, and biological interpretation.

Rebuttal Fig. 2
(A) UMAP plots of integrated scRNA-seq data showing the zonation annotation. (B) UMAP plots of integrated scRNA-seq data showing the distribution of ploidy subpopulations across mononuclear diploid (2n×1), mononuclear tetraploid (4n×1), binuclear diploid (2n×2), mononuclear octoploid (8n×1), and binuclear tetraploid (4n×2) cells. (C) UMAP plots show hepatocyte ploidy subpopulation distribution from this study dataset.

3. There were a few wirtten errors, i.e. Fig.4C-G Fig.4H should be Fig.5.

Our reply: Thank you for pointing out this error. We have revised the manuscript accordingly and carefully checked for any remaining typographical mistakes elsewhere.

Reviewer #2: This study develops an imaging-based ploidy identification (SCIPI) pipeline using the Stereo-cell spatially resolved single-cell sequencing technology. It successfully achieves, for the first time, the simultaneous acquisition of transcriptomic and ploidy information from the same cell, systematically resolving the spatial distribution and functional heterogeneity of four key hepatocyte polyploid subtypes (2n×1, 2n×2, 4n×1, 4n×2) in mice. The technical design is highly innovative, the data quality is robust, and the work represents a significant advance in the field of hepatic polyploidy. I recommend conditional acceptance pending revisions, which should address the need for additional key validations and more detailed data analysis in certain areas.

We sincerely appreciate your recognition of our manuscript. You have also pointed out several urgent issues that need to be addressed in our research and provided valuable insights for solving them—this has been extremely helpful to us, further enriching and enhancing the significance of our manuscript and making our conclusions more robust. Following your suggestions, we have supplemented several experiments and analytical content, revised errors in the manuscript, and filled in the insufficient parts. Below are the specific revisions; please kindly review them.

1.Weaknesses and suggestions
(1)Technical validation requires sreangthening
Issue: The accuracy of SCIPI's ploidy classification relies on nuclear area measurement and K-means clustering but lacks systematic validation against a gold standard (e.g., DNA content quantification by flow cytometry).
Suggestions:
a.Perform flow cytometric DNA content analysis on a random subset of cells and compare the results with SCIPI classifications (e.g., using a confusion matrix) to establish concordance.

b.Validate the linear relationship between nuclear area and DNA content (e.g., by isolating hepatocyte nuclei and quantifying DAPI fluorescence intensity).

Our reply: Your suggestion is highly critical, as this validation was indeed missing from

our previous work. We performed correlation analysis between DAPI fluorescence intensity and the corresponding nuclear area using FACS-sorted ploidy subpopulations, with three biological replicates. The relevant results have been updated in the manuscript (new Fig. 3H-M, Fig. S1B-G). For the FACS-sorted diploid (2c), tetraploid (4c), and octoploid (8c) hepatocytes, correlation analysis between the DAPI fluorescence intensity and nuclear area of each ploidy subtype showed a strong positive correlation, with a coefficient of 0.82 ($p=2.7\times10^{-5}$). Additionally, the fluorescence intensity of FACS-sorted diploid, tetraploid, and octoploid cells was highly positively correlated with the nuclear area of corresponding cell types identified by SCIPI ($R=0.9$, $p=5.1\times10^{-7}$).

In summary, nuclear area can serve as a reliable proxy for DNA content in ploidy identification. These results further validate the rationale and accuracy of the SCIPI technique for ploidy classification based on nuclear area measurement.

(2) Biological mechanism exploration in superficial
Issue: The study is primarily descriptive. The mechanistic drivers behind the functional differences among polyploid subtypes (e.g., why 4n×2 cells show enhanced metabolic gene expression) are not deeply explored.
Suggestions:
a.Perform pathway enrichment analysis (e.g., on metabolic pathways, cell cycle pathways) on differentially expressed genes to elucidate the molecular basis of functional enhancements in polyploids.

b.Integrate with published scRNA-seq datasets of hepatocytes to investigate if specific polyploid subtypes correspond to distinct functional states (e.g., oxidative metabolism, detoxification).

Our reply: Thank you for your valuable suggestion! We have additionally performed differential expression analysis and functional enrichment analysis based on ploidy groups and distinct karyotype-resolved ploidy subtypes and the relevant results have been updated in the manuscript (new Fig. 5A–G , Fig. S3A–C). These analyses primarily characterize the molecular alterations that occur in distinct polyploid groups compared with diploid hepatocytes.

We observed that tetraploid hepatocytes exhibited fewer upregulated differentially expressed genes (DEGs) compared to octoploid cells. Furthermore, the set of genes upregulated in octoploid cells encompassed those upregulated in tetraploid cells. In contrast, no upregulated DEGs were detected in diploid cells relative to polyploid hepatocytes. Gene Ontology (GO) enrichment analysis of these DEGs revealed significant enrichment in pathways related to cytoplasmic translation, small molecule catabolism, and generation of precursor metabolites and energy, reflecting enhanced metabolic and biosynthetic activity in octoploid cells.

To dissect differences attributable specifically to nuclear number independent of total DNA content, we next compared mononucleated and binucleated polyploid cells. We found that binucleated hepatocytes, both within tetraploid and octoploid populations, displayed a greater number of upregulated DEGs compared to their mononucleated counterparts. Notably, the DEGs upregulated in both mononucleated and binucleated tetraploid cells were largely contained within the octoploid DEG set. GO analysis of these DEGs showed that both tetraploid and octoploid hepatocytes shared enrichment in pathways including oxidative phosphorylation, ATP synthesis coupled electron transport, and aerobic respiration. Additionally, octoploid cells were uniquely enriched in pathways related to small molecule metabolism, organic acid metabolism, and fatty acid metabolism.

We integrated the sequencing data obtained by SCIPI with a published single-cell transcriptomic dataset of homeostasis adult mouse hepatocytes (DOI: 10.1038/s41588-024-01709-7). While the SCIPI data integrated well with the published dataset, we observed that hepatocytes of different ploidy subtypes exhibited an irregularly intermixed and seemingly random distribution, regardless of integration (see Rebuttal Fig. 2B-C). Consequently, it was not feasible to assign the ploidy information from SCIPI to the cells in the published scRNA-seq dataset using methods based on

clustering or spatial proximity projection. Therefore, regarding hepatocyte ploidy subtypes, we were unable to perform a combined analysis with the published data to conduct a more in-depth exploration of functional pathway differences across ploidies.

(3)Data statistics and presentation need optimization
Issue:Statistical significance is not indicated in figures 3D and 4C-E; The heatmap in figure 5B lacks functional annotations for gene clusters, making interpretation difficult.

Our reply: Thank you for your valuable comments. We have now updated the relevant figures accordingly. In Fig. 3D we performed significance tests and added error bars along with overlaid scatter plots of the data. In Fig. 4C–E, we have also included significance testing. These statistical methods are described in the corresponding figure legends of the manuscript. Furthermore, we have comprehensively revised the heatmap originally shown in Fig. 5B to improve its clarity and readability, now presented as new Fig. 5C. Additionally, relevant gene enrichment analysis has been included as new Fig. 5D.

(4)Discussion of technical limitations should be deepened
Issue: Potential RNA diffusion between adjacent cells is mentioned but its specific impact on the polyploid transcriptome data is not assessed.

Our reply: Since the Stereo-cell system employs an open reaction setup, RNA diffusion between neighboring cells is theoretically possible. As reported in the published Stereo-cell methodology paper, the peak proportion of heterologous RNA in individual cells after co-seeding human-mouse mixed cells onto the Stereo-cell chip is ~0.027, a value that does not compromise data accuracy (DOI: 10.1126/science.adr0475). In our study, we addressed this potential issue through multiple experimental and computational strategies. Experimentally, we optimized the cell loading density on the chip to achieve a moderate cell density, which spatially minimizes diffusion effects. During data quality control, we implemented a multi-modal imaging-based cell contour QC pipeline to filter out cells with abnormal nucleus-to-cytoplasm ratios or positional overlap. We further employed a deep learning model (StarDist) to enhance segmentation accuracy for multinucleated and polyploid cells. Subsequently, spatial transcript clustering analysis was performed to verify that transcriptomic signals align with cellular morphology.

Through this integrated approach—encompassing density optimization, morphological filtering, multi-modal QC, high-precision computational segmentation, and spatial transcript verification—we obtained high-quality Stereo-cell data for hepatocytes. Moreover, our hepatocyte data show excellent integration with published single-cell liver datasets. In conclusion, we believe the potential impact of diffusion on our final data is minimal.

(5)Lack of details crucial for reproducibility
Issue: Key parameters like cell adhesion efficiency and RNA capture efficiency are not quantified.
Suggestions:
a.Quantify and report hepatocyte adhesion rates to the chip and RNA capture efficiency (perhaps compared to standard scRNA-seq protocols).

Our reply: We acknowledge the initial omission in our data reporting and have now supplemented the relevant statistical analyses. As demonstrated in the published Stereo-cell technology article, direct comparison on PBMC samples showed that Stereo-cell outperformed 10x Chromium in terms of RNA capture efficiency and specificity (DOI: 10.1126/science.adr0475). In our study, for the two independent biological replicates (hep56_H8 and hep56_M1), a high inter-replicate correlation was observed (R^2=0.87), confirming the technical robustness and reproducibility of the Stereo-cell platform (manuscript, Fig. S1A). Regarding the experimental details, we loaded 5,573 cells onto the Stereo-chip for sample hep56_H8, with 3,164 cells retained for analysis after quality control, resulting in an effective cell recovery rate of ~57%. For sample hep56_M1, 2,148 cells were loaded, and 1,724 cells passed QC, yielding an effective recovery rate of ~80%. The overall cell capture performance was satisfactory, though some variation existed between replicates. This variation may be attributed to our stringent quality control criteria, which were implemented to ensure high-quality,

Powered by Editorial Manager® and ProduXion Manager® from Aries Systems Corporation

	low-contamination single-cell data.<br><br>The RNA capture metrics for the two biological replicates were as follows: for hep56_H8, the mean gene count was 444 and the mean UMI count was 2,136; for hep56_M1, the mean gene count was 688 and the mean UMI count was 3,785 (see below Rebuttal Fig. 3A). The calculated RNA capture efficiency ranged from 4.7% to 5.2%, with a mitochondrial read proportion between 1.2% and 3.5%. These values are moderately lower than the median gene count (~1,000, Liang et al., see below Rebuttal Fig. 3B) reported for P56 hepatocytes captured using the 10x Genomics Chromium platform (DOI: 10.1016/j.devcel.2022.01.004).<br><br><br>Rebuttal Fig. 3<br>(A) Quality control of hepatocyte Stereo-cell sequencing dataset from our study. (B) The number of genes detected in hepatocytes at each chosen time point from Liang et al.'s .<br><br>b.Ensure the GitHub code repository includes example test data and detailed setup/execution guidelines.<br><br>Our reply: We have now updated the documentation on GitHub, where a detailed tutorial has been added to the GitHub introduction page and workable example data has been uploaded. You may access the updated content at the following link: https://github.com/JeffLuo9/Hep_Ploidy_protocol/.<br><br>2.Specific revision recommendations<br>(1)Methods: Clarify the normalization method for nuclear areas used in K-means clustering (e.g., is it corrected for cell size variation?).<br><br>Our reply: For K-means clustering, the nuclear area was standardized solely by converting from pixel units to square micrometers (μm²). Beyond this conversion, no additional correction or normalization of nuclear or cellular area was performed. The detailed procedure has been added to the Results section of the manuscript on lines 126-131.<br><br>(2)Results: Include a direct transcriptomic comparison between 4n×1 and 2n×2 cells to explore the specific influence of nuclear number independent of total ploidy.<br><br>Our reply: We further investigated the functional differences associated with nuclear number, independent of total DNA content. The corresponding results have been added to the Results section of the manuscript on lines 259-268 (new Fig. 5E-I, Fig. S3A-D).<br><br>(3)Discussion: Compare the advantages and limitations of the SCIPI approach for polyploid analysis against other recent spatial omics technologies (e.g., MERFISH).<br><br>Our reply: The advantages and limitations of the SCIPI technique compared to other methods, such as FACS-sorted scRNA seq and MERFISH, have been added to the Discussion section of the manuscript on lines 310-326.<br><br>(4)Figures: For Figure 4B, consider adding quantitative statistics for spatial distribution (e.g., density curves along the CV-PV axis).<br><br>Our reply: We have updated Fig. 4B by adding density curves to visualize the CV-PV axis scores across different ploidy subtypes (manuscript, new Fig. 4B–C).
**Additional Information:**	
**Question**	**Response**
Are you submitting this manuscript to a special series or article collection?	No
**Experimental design and statistics**	Yes

Powered by Editorial Manager® and ProduXion Manager® from Aries Systems Corporation

Full details of the experimental design and statistical methods used should be given in the Methods section, as detailed in our Minimum Standards Reporting Checklist. Information essential to interpreting the data presented should be made available in the figure legends.<br><br>Have you included all the information requested in your manuscript?	
**Resources**<br><br>A description of all resources used, including antibodies, cell lines, animals and software tools, with enough information to allow them to be uniquely identified, should be included in the Methods section. Authors are strongly encouraged to cite Research Resource Identifiers (RRIDs) for antibodies, model organisms and tools, where possible.<br><br>Have you included the information requested as detailed in our Minimum Standards Reporting Checklist?	Yes
**Availability of data and materials**<br><br>All datasets and code on which the conclusions of the paper rely must be either included in your submission or deposited in publicly available repositories (where available and ethically appropriate), referencing such data using a unique identifier in the references and in the "Availability of Data and Materials" section of your manuscript.<br><br>Have you have met the above requirement as detailed in our Minimum Standards Reporting Checklist?	Yes
GigaScience has policies and guidelines in place for the use of generative AI-	No

writing tools such as ChatGPT. If you have used such writing tools to assist with writing the manuscript this must be declared and cited in the text. Authors should not list AI-writing tools and other AI-assisted technologies as an author or co-author and should acknowledge that they are fully responsible for text generated or refined by AI-writing tools.<p><br><br>A summary of use (particularly in the introduction or among methods) needs to be included at the end of the paper, and the outputs should also be included as a supplementary file hosted in GigaDB or other open repositories. Please read our guidelines for more information. <p><br><br>By submitting to GigaScience, you are aware of the journal's AI-writing tools policy, and if you have declared use of such tools below, you have acknowledged this where appropriate in your manuscript and have made a summary of use and outputs available. <p><br><b>AI-assisted writing tools have been used in the preparation of this manuscript?	

Click here to access/download;Manuscript;GS_manuscript_revised_Final_2

Stereo-cell deciphers the spatial and functional heterogeneity of polyploid hepatocytes

Yongqing Yang[1,2,†], Jiahui Luo[2,3,†], Yier Cai[4], Pengcheng Guo[2,3,5], Qiang Guo[2], Hong Wu[1,2], Longqi Liu[2,5] and Shijie Hao[2,3,5,*]

[1]College of Life Sciences, University of Chinese Academy of Sciences, Beijing 100049, China.
[2]BGI Research, Hangzhou 310030, China.
[3]School of Biology and Biological Engineering, South China University of Technology, Guangzhou 510006, China.
[4]Guangdong Country Garden School, Foshan 528311, China.
[5]State Key Laboratory of Genome and Multi-omics Technologies, BGI Research, Hangzhou 310030, China.
[†]These authors contributed equally to this work.
[*]To whom correspondence should be addressed: Shijie Hao, E-mail: haoshijie@genomics.cn.

Abstract

A characteristic feature of the liver is the presence of numerous polyploid hepatocytes. However, the functional distinctions among diploid, tetraploid, and octoploid hepatocytes remain poorly understood. In this study, we employed the spatially resolved single-cell sequencing technology, Stereo-cell, to dissect the transcriptomic and functional heterogeneity across hepatocyte ploidy subtypes. We detail the development of Stereo-cell Imaging-based ploidy Identification (SCIPI), a technical pipeline that integrates bright-field cell contour recognition, DAPI-based nuclear area and number quantification, and UMI-barcoded single-cell transcriptomics. This approach enables precise identification of six core hepatocyte subtypes: mononucleated diploid (2n×1), mononucleated tetraploid (4n×1), binucleated tetraploid (2n×2), mononucleated octoploid (8n×1), binucleated octoploid (4n×2), and binucleated hexadecaploid (8n×2) hepatocytes. Single-cell transcriptomic analysis based on ploidy annotation revealed that gene expression levels scale positively with increasing ploidy and nuclear number. Metabolic pathway-associated genes were significantly upregulated in polyploid cells, suggesting that cellular polyploidization enhances the metabolic activity of hepatocytes. Furthermore, this SCIPI

strategy is broadly applicable to the study of various polyploid tissues, offering a novel and versatile framework for innovative ploidy-resolved research across diverse biological researches.

Background

Polyploidy, a state characterized by cells containing more than a diploid DNA content, represents a physiological function and a biological phenomenon of particular significance in the mammalian liver[1-5]. The liver has the ability to dynamically adjust its ploidy constituent throughout development and in response to diverse stresses, employing polyploidization as a fundamental mechanism to govern growth regulation and functional adaptation[6,7]. Tetraploid hepatocytes are predominantly found in the liver and arise as a result of a developmentally programmed cytokinesis failure during postnatal maturation[6,8,9]. The formation of tetraploid hepatocytes begins during the weaning transition, where a diploid hepatocyte either completes normal cytokinesis to generate two diploid cells or undergoes incomplete division to form a binucleated tetraploid (2n×2)[9-11]. Hepatocytes of even higher ploidy are generated from binucleated tetraploids via aberrant cell cycles[12,13]. Polyploidization is critically regulated by the PI3K-AKT pathway[14], MAPK pathway[15], and E2F transcription factors[16-18], and knockdown of E2F8 demonstrates a decrease in polyploidy in the liver. Previous studies have suggested that polyploidy serves as a genomic buffer to enhance organ resilience under physiological conditions[19], but accelerates liver dysfunction in the pathological process[20-22]. Upon hepatocyte-specific knockout of the master regulators HNF4A or CEBPA, polyploid hepatocytes buffer the genetic lesion by non-randomly selecting the wild-type allele, thereby compensating for the haploinsufficiency and alleviating senescence in the liver through the enrichment of completely wild-type nuclei[23]. Premature weaning or knockdown of E2F8 to alter liver ploidy revealed that polyploidy acts as a tumor suppressor to prevent oncogenic progression in the liver[24]. In contrast, in nonalcoholic fatty liver disease (NAFLD), oxidative stress activates the ATR/p53/p21 DNA damage checkpoint, promoting endoreplication that generates highly polyploid mononuclear cells which facilitate hepatocellular carcinoma (HCC) development[22]. Hepatocyte-specific RAD51[25] or PRMT5[26] knockout also induces

G2/M arrest and promotes the accumulation of highly polyploid mononucleated cells, ultimately leading to progression to fibrosis and HCC. These findings imply polyploidization as a double-edged sword, physiologically beneficial yet pathologically malignant when dysregulated. However, the precise hepatocyte ploidy subtypes and underlying functional heterogeneity remain largely unexplored.

The state of hepatocyte ploidy is accompanied by a concomitant increase in nuclear size[15]. Two primary approaches have been used to study hepatocyte polyploidy, each with inherent limitations. The first method involves co-staining liver cryosections with nucleic acid and plasma membrane markers by identifying ploidy through measurement of maximum nuclear diameter or area in situ[15,27]. While this technique classifies all ploidy subtypes, it does not connect the ploidy state with transcriptomic information. The second method is fluorescence-activated cell sorting (FACS), which is employed to isolate cells or nuclei based on nucleic acid fluorescence intensity[28,29]. The sorted diploid, tetraploid, and higher-ploidy populations obtained through this method can subsequently be analyzed by single-cell RNA-seq (scRNA-seq) or single-nucleus RNA-seq (snRNA-seq). Although polyploidy can be characterized with FACS at the single-cell level, it must be noted that this method fundamentally loses the critical biological distinction between mononucleated and binucleated polyploid subtypes.

To address these limitations, we developed Stereo-cell[30-35] imaging-based ploidy identification (SCIPI), a comprehensive pipeline which uniquely integrates spatial transcriptomics with imaging to enable simultaneous transcriptome profiling and ploidy determination within the same cell[36]. The strategy precisely classifies six key hepatocyte ploidy subtypes: mononucleated diploid (2n×1), mononucleated tetraploid (4n×1), binucleated tetraploid (2n×2), mononucleated octoploid (8n×1), binucleated octoploid (4n×2), and binucleated hexadecaploid (8n×2) hepatocytes. We performed single-cell transcriptomic analysis of these polyploid hepatocytes, constructing transcriptomic profiles of ploidy subtypes and providing a comprehensive resource for gene expression patterns in hepatocyte polyploidy.

Results

Integrated Stereo-cell matrix of gene expression and ploidy

Polyploidization is critical for liver development, functional maturation, and disease pathogenesis[12]. To investigate the functional implications of under characterized hepatocyte ploidy subtypes, we isolated single hepatocytes from C57BL/6 mice at 8 weeks of age using an optimized two-step collagenase perfusion protocol[37-43] to obtain highly viable cells. Due to the sensitivity of primary hepatocytes to handling-induced death, Stereo-cell[33] sequencing was performed immediately to ensure viability. We conducted two biologically independent replicates (hep56_H8 and hep56_M1) in total. Given the large size of hepatocytes, we loaded a total of 7,721 cells onto two Stereo chips to achieve an optimal cell density the chip surface. After quality control, 4,888 cells were obtained, resulting in an overall cell capture rate of 68.5%. High reproducibility was observed between the two biological replicate chips (supplementary **Fig. S1A**).

To obtain high-confidence identification of polyploid hepatocytes, we established the SCIPI multimodal analytical framework by integrating spatially resolved transcriptional profiles with the concurrent bright-field images and DAPI-based nuclear quantification (**Fig. 1A**). This tripartite data architecture enables the systematic segmentation of individual hepatocytes, followed by automated ploidy classification through nuclear area quantification that distinguishes mononucleated and binucleated subtypes (**Fig. 1B**). Identity-matched matrices subsequently align these ploidy annotations with their corresponding transcriptomes while preserving spatial coordinates, permitting comprehensive ploidy-resolved single-cell analysis. The whole experimental workflow from hepatocyte isolation to sequencing typically requires 4 days. SCIPI integrates the multimodal data (imaging and transcriptomics) described above, requiring approximately 1 day from raw data to final ploidy annotation.

Fig. 1 Comprehensive Stereo-cell experimental workflow for hepatocytes

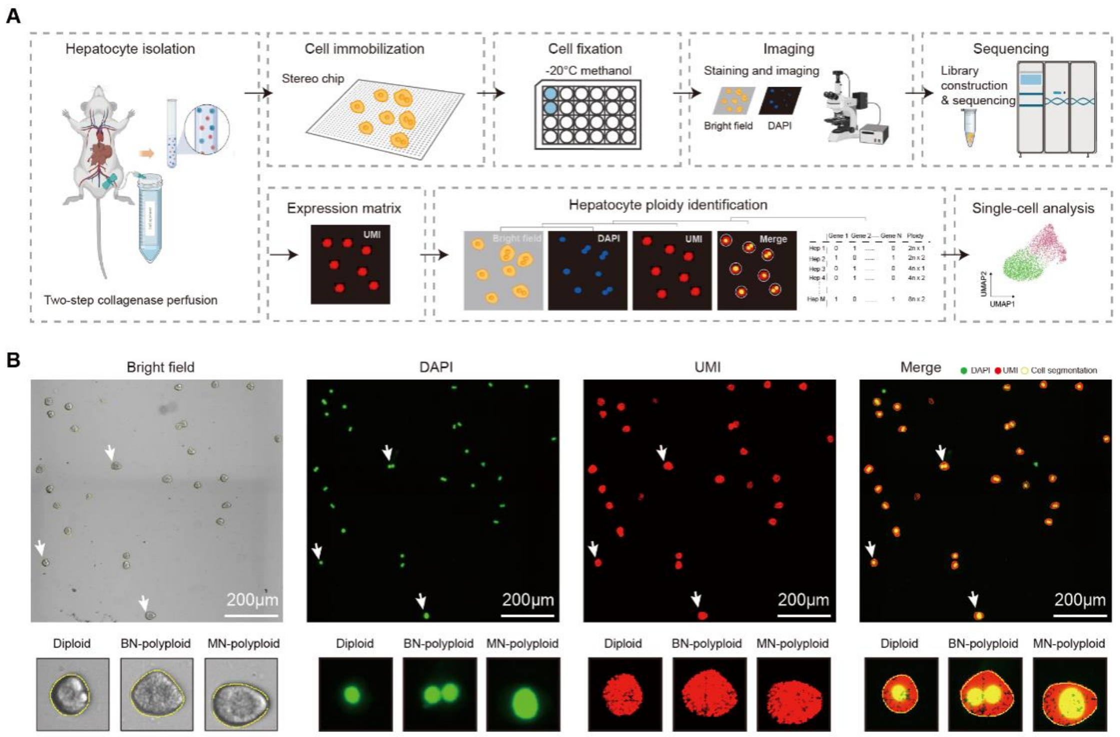

Figure 1 Comprehensive Stereo-cell experimental workflow for hepatocytes. (A) Schematic of the Stereo-cell experimental workflow for hepatocytes. (B) Representative images show hepatocytes across multiple fields of view on the Stereo chip. Arrowheads indicate selected cells. BN-polyploid (binucleated polyploid hepatocyte); MN-polyploid (mononucleated polyploid hepatocyte). Scale bar: 200 µm.

Ploidy identification workflow and accuracy assessment

The Stereo-cell raw sequencing data was processed with SAW[44] to obtain GEM-format files that have undergone sequence demultiplexing and alignment. This file contains Gene IDs, gene coordinates (x, y), molecular identifier count (MID Count), and exon counts. Based on this information, we can generate in situ UMI counts image (UMI image) with script gem2mask.py. (**Fig. 1A, UMI**). Then, staining DAPI image was registered with UMI image. Perform cell segmentation on the DAPI image that has been registered to the UMI image and on the brightfield image. According to DAPI masks, cell masks, DAPI area and nuclear count per cell to classify hepatocyte ploidy subtypes (**Fig. 2A**). To classify hepatocytes into mononucleated diploid, tetraploid, and octoploid subtypes, we applied k-means clustering to all nuclei segmented by StarDist. This algorithm groups nuclei into clusters by maximizing between-

cluster differences in nuclear area, a feature that correlates with the stepwise increase in DNA content from diploid to polyploid states. Based on this nuclear classification, we then assigned a ploidy status to each individual cell.

The accuracy of ploidy identification is primarily monitored through the following key steps. Firstly, ensure perfect registration between DAPI image and UMI image across multiple regions, with UMIs remaining untransformed. Switch between brightfield (green channel) and UMI (red channel) to confirm that cell boundaries in brightfield images align with UMI image in multiple selected regions. Secondly, assess cell size parameters during cell segmentation with Cellpose[45]. Manually verify that the majority of hepatocyte boundaries are accurately identified post-segmentation, and adjust size settings if necessary (Cellpose's automatic size estimation is generally reliable). Finally, cross-validate results using original DAPI, brightfield, and nuclear feature maps. Since the majority of cell nuclei are either single or double, and although a few reports have mentioned cells with three nuclei, for the sake of universality and accuracy, only cells with 1 or 2 nuclei are retained here for accuracy testing. Ensure the misidentification error within multiple randomly selected region is below 3 cells per region (**Fig. 2B**).

Fig. 2 SCIPI pipeline for spatially resolved hepatocyte ploidy profiling

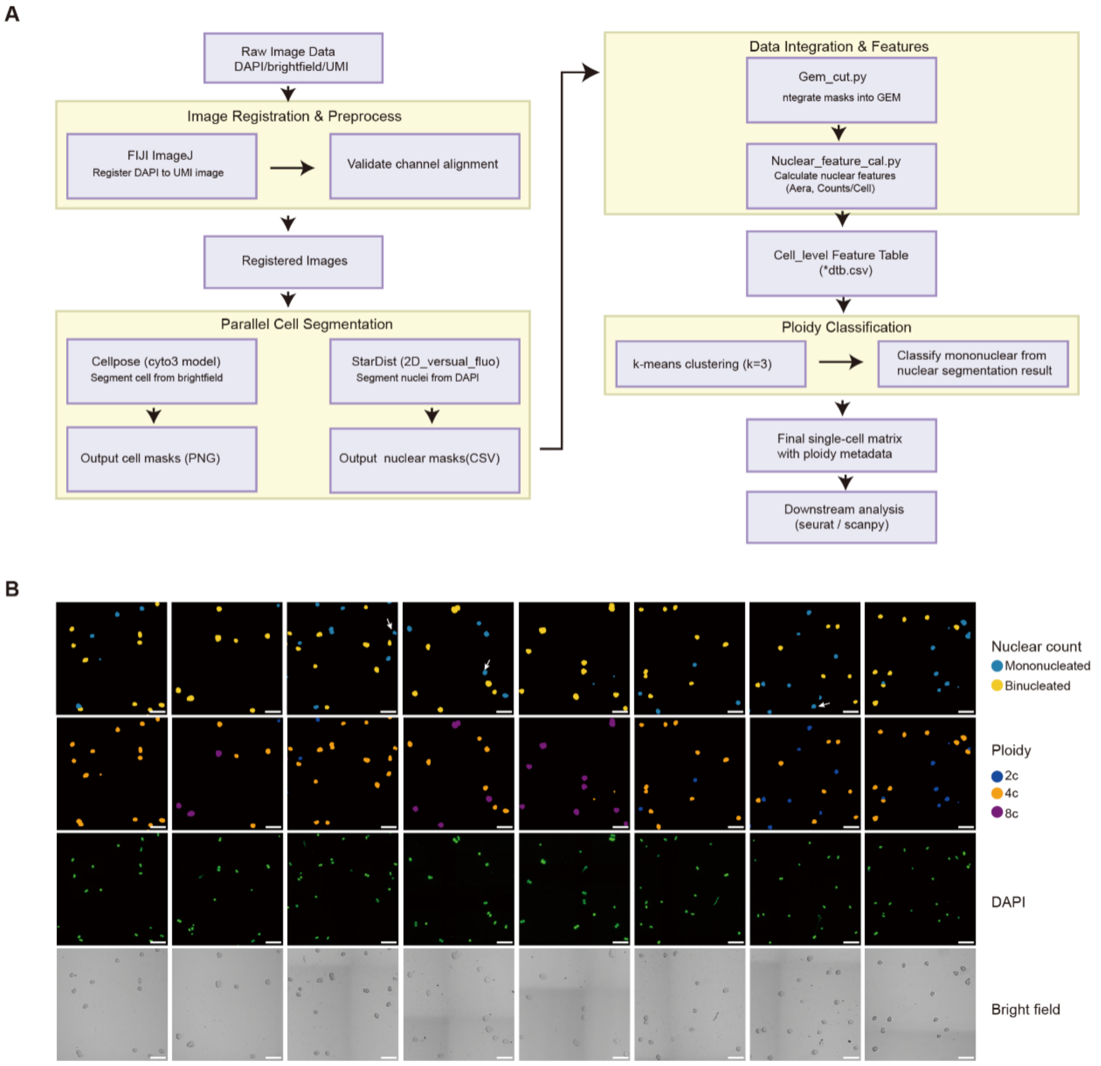

Figure 2 SCIPI pipeline for spatially resolved hepatocyte ploidy profiling. (A) Workflow for identifying hepatocyte ploidy subtypes. (B) Representative field showing identification of hepatocyte ploidy subtypes. Arrows indicate misidentified cells. Scale bar: 100 μm.

SCIPI for quantifying polyploid hepatocyte subpopulation

Polyploidization constitutes an essential biological feature of mammalian hepatocytes, with developmental programming driving nuclear DNA accumulation through both cytokinesis failure and nuclear duplication[8]. In our staining image, the nuclear area of polyploid hepatocytes was significantly larger than that of diploid nuclei (**Fig. 3A**), consistent with previously reported studies showing a linear correlation between nuclear area and DNA

content[15]. Based on nuclear area distribution, we classified hepatocytes into six ploidy classes (**Fig. 3B**): 2n×1: diploid; 4n×1: mononucleate tetraploid; 2n×2: binucleate tetraploid; 8n×1: mononucleate octoploid; 4n×2: binucleate octoploid; 8n×2: binucleate hexadecaploid. Distinct nuclear area distribution patterns were observed across diploid (2c), tetraploid (4c), octoploid (8c) and hexadecaploid (16c) cells levels, whereas similar nuclear area distribution profiles were exhibited by different nuclear types within the same ploidy level.

Quantitative analysis revealed that polyploid hepatocytes accounted for over 85% of total hepatocytes in P56 mice (**Fig. 3C-D**). The ploidy distribution was as follows: 11.4% diploid, 66.2% tetraploid (33.9% mononucleated and 32.3% binucleated), 21.2% octoploid (13.0% mononucleated and 8.2% binucleated), and 1.8% hexadecaploid. This distribution is consistent with previously reported proportions in adult mouse liver[10,46-48]. To further validate the robustness of the SCIPI method for ploidy identification, we performed immunofluorescence staining on liver tissue sections and applied the same ploidy recognition strategy. This analysis yielded a similar overall ploidy distribution profile, and no significant difference was observed in the identification of polyploid versus diploid cells between the SCIPI-based and tissue-section-based methods (**Fig. 3 F-G**).

To confirm that the Stereo-cell platform underlying SCIPI technology captures all hepatocyte ploidy types without bias, we performed fluorescence-activated cell sorting (FACS) to isolate diploid, tetraploid, octoploid, and hexadecaploid hepatocytes. The resulting ploidy proportions were highly consistent with those obtained by SCIPI **(Fig. 3H,** supplementary **Fig. S1B-F**). Furthermore, the FACS-sorted diploid, tetraploid, and octoploid hepatocytes were plated on slides for microscopic imaging (**Fig. 3I-K**). Statistical analysis of the DAPI fluorescence intensity versus the corresponding nuclear area for these FACS-sorted cells showed a significant positive correlation ($R=0.82$, $p=2.7e-05$, **Fig. 3L**). Finally, we assessed the consistency between the fluorescence intensity of FACS-sorted cells of different ploidies and the nuclear area measured by SCIPI for corresponding ploidy classes. A high correlation was also observed here ($R=0.9$, $p=5.1e-07$, **Fig. 3M**). In summary, these results demonstrate the accuracy and reliability of the SCIPI method for classifying hepatocyte ploidy.

Fig. 3 Quantifying and classifying hepatocyte polyploidy

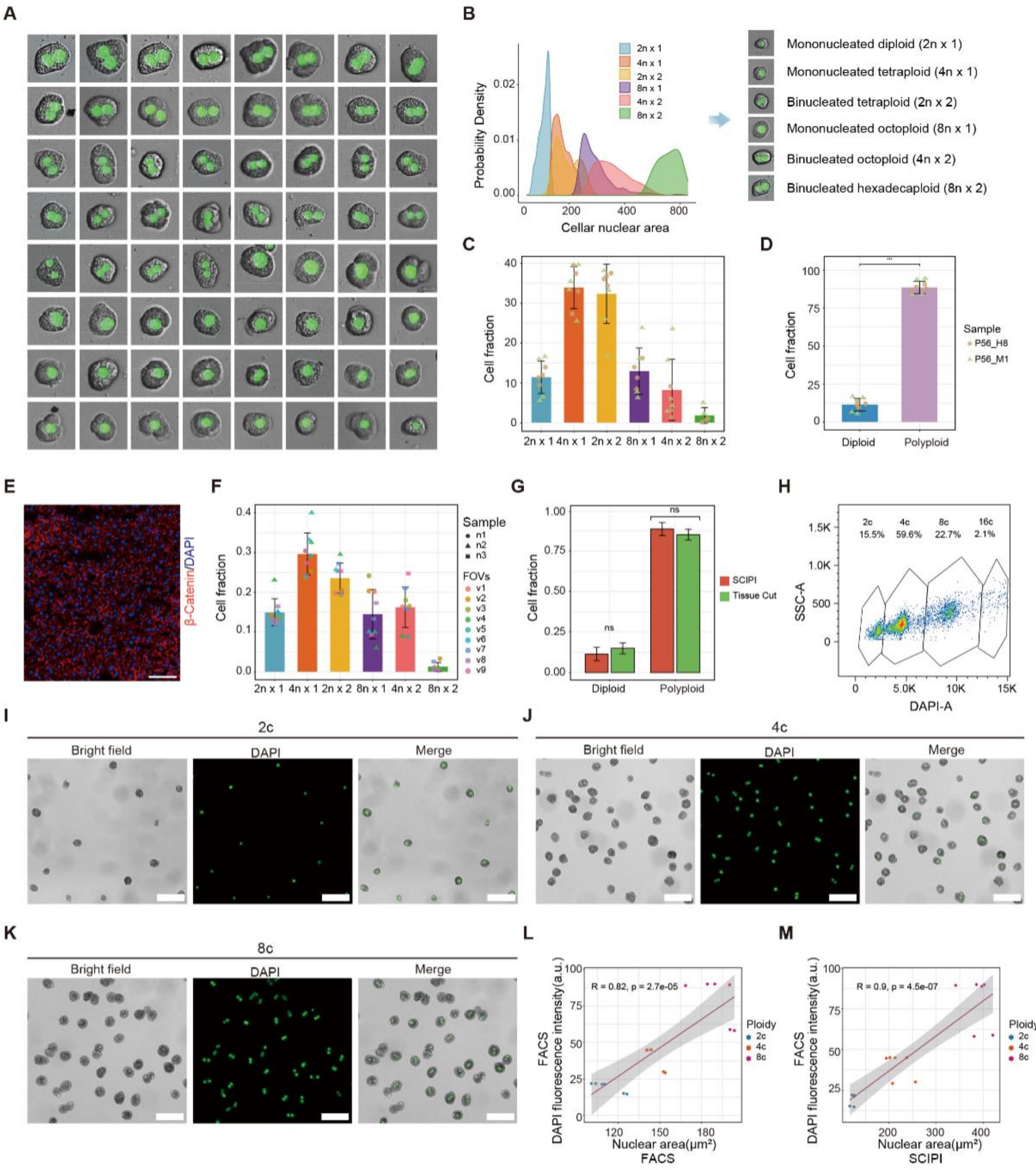

Figure 3 Quantifying and classifying hepatocyte polyploidy. (A) Representative hepatocytes with varying nuclear sizes, showing binucleated and mononucleated types. DAPI (green). (B) Density curves for nuclear area distribution of hepatocyte ploidy subpopulations (2n×1: diploid; 4n×1: mononucleate tetraploid; 2n×2: binucleate tetraploid; 8n×1: mononucleate octoploid; 4n×2: binucleate octoploid; 8n×2: binucleate hexadecaploid). (C) Proportions of the hepatocyte ploidy subpopulations as identified by SCIPI. (D) Proportion of diploid and polyploid hepatocytes in SCIPI. Two-sided unpaired Student's t-test; *p<0.05, **p<0.01, ***p<0.001; ns, no significance. (E) Immunofluorescence of β-Catenin (red) and DAPI (blue) in P56 C57BL/6 mouse liver sections (n=3 biologically independent animals). Scale bar: 100 μm. (F) Hepatocyte ploidy subpopulation fractions in P56 C57BL/6 mouse liver sections. Data

are from 3 mice (n1–n3), with 3 random fields of view (v1–v9) per mouse. (G) Hepatocyte ploidy fractions (diploid vs. polyploid) from SCIPI (red) and liver section analysis (green). Two-sided unpaired Student's t-test; ns, no significance. (H) Flow cytometric DNA content analysis (DAPI staining) of hepatocytes, showing diploid (2c, 15.5%), tetraploid (4c, 59.6%), octoploid (8c, 22.7%) and hexadecaploid (16c, 2.1%) cells. (I-K) Brightfield, DAPI, and merged images of flow-sorted diploid (2c) in (I), tetraploid (4c) in (J), and octoploid cells in (K). Scale bar, 100 µm. (L) Correlation between nuclear area and fluorescence intensity in flow-sorted diploid, tetraploid, and octoploid hepatocytes (n=3 P56 C57BL/6 mice; R=0.84, p=2.7e-05). (M) Correlation between nuclear area (measured by SCIPI) and DAPI fluorescence intensity (assessed by FACS) in flow-sorted 2c, 4c, and 8c hepatocytes (n=3 P56 C57BL/6 mice; Pearson correlation R= 0.9, p= 4.5e-07).

Polyploid hepatocyte distribution across liver lobule

Following ploidy identification, we obtained high-quality single-cell data annotated with ploidy information. These data were integrated with a published single-cell transcriptomic dataset of homeostasis adult mouse hepatocytes[32], demonstrating successful integration (supplementary **Fig. S2A, S2C-H**). Regardless of integration, hepatocytes of different ploidy subtypes showed an irregularly intermixed distribution in uniform manifold approximation and projection (UMAP) (**Fig. 4A,** supplementary **Fig. S2B**).

Based on the expression of liver zonation marker genes (e.g., pericentral marker *Cyp2e1* and periportal marker *Cyp2f2*), we categorized hepatocytes into pericentral (PC) and periportal (PP) lobule zone subpopulations (**Fig. 4A**). Weighted gene signature scoring for PC- and PP-specific genes allowed us to accurately mapped the spatial distribution of different hepatocyte ploidy subtypes across the hepatic lobule (**Fig. 4B-C**). Due to low cell counts and high statistical variance, hexadecaploid hepatocytes were excluded from this analysis. The lobule distribution patterns of SCIPI-identified hepatocyte ploidy subtypes aligned with results from previous studies based on nuclear and membrane staining of liver tissue sections[15]. SCIPI analysis revealed significant spatial distribution features: 4n×2 and 8n×1 hepatocytes were enriched in the pericentral region, whereas 2n×1 and 2n×2 hepatocytes were predominantly located in the

periportal region, suggesting a preferential enrichment of higher-ploidy hepatocytes in the pericentral zone.

To investigate the expression dynamics of zonation-related genes across different hepatocyte ploidy states, we analyzed the expression patterns of central vein (CV)-enriched marker genes (*Cyp2e1*, *Cyp1a2*, *Cyp27a1*) and portal vein (PV)-enriched marker genes (*Cyp2f2*, *Gls2*, *Hal*) along the hepatic lobular axis (**Fig. 4D**). The expression gradients of these genes along the CV-to-PV axis were consistent with the well-characterized zonation patterns reported previously[28,32,49,50].

Subsequently, we stratified the analysis by five hepatocyte ploidy subtypes (2n×1, 4n×1, 2n×2, 8n×1, 4n×2) (**Fig. 4E**). Most genes retained a conserved zonated expression trend across these subtypes. However, their expression levels along the CV-PV axis showed significant differences correlated with increasing ploidy.

Fig. 4 Gene expression along CV to PV trajectory by ploidy

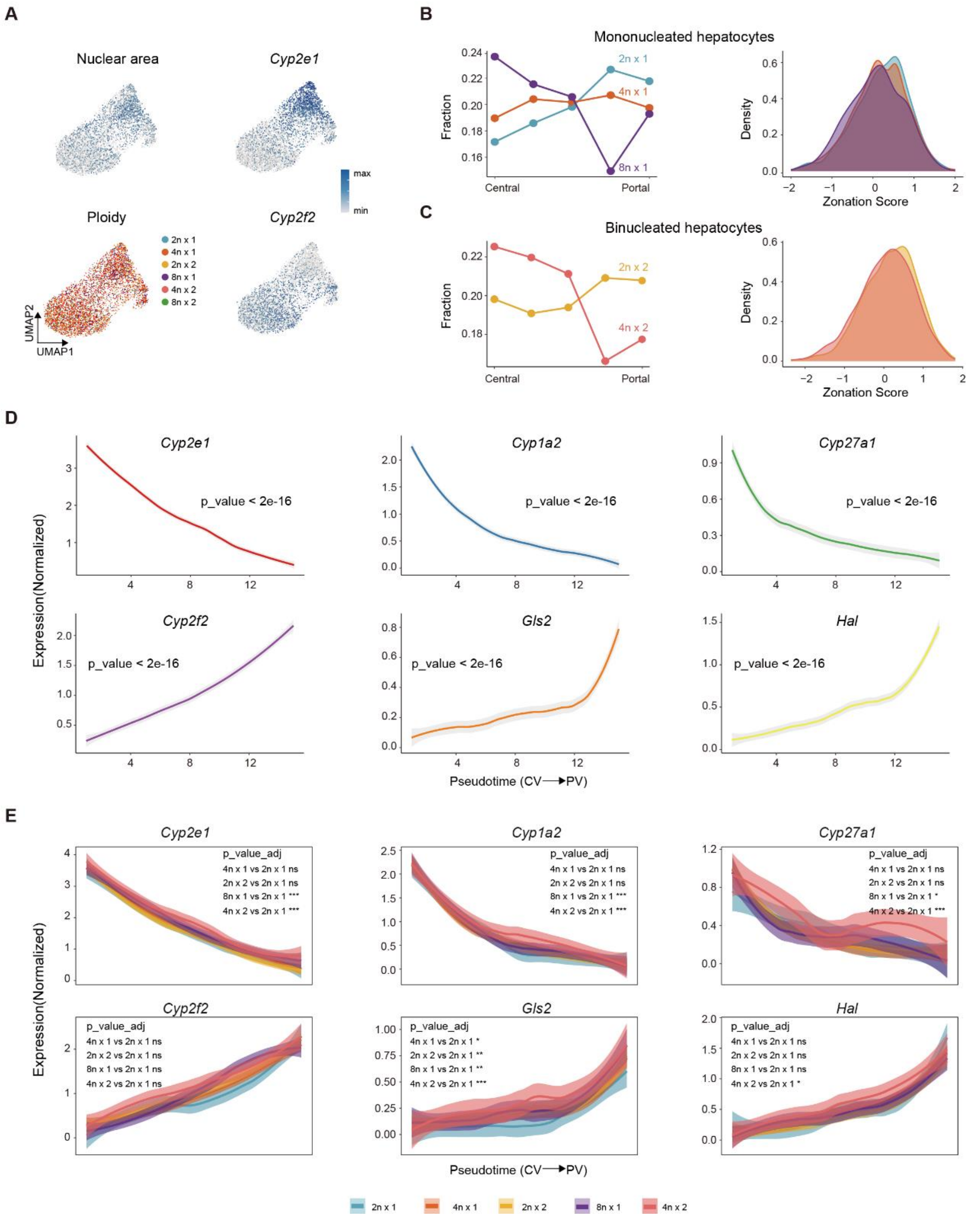

Figure 4 Gene expression along CV to PV trajectory by ploidy. (A) UMAP plots show hepatocyte nuclear area, ploidy subpopulation distribution, and expression of zonation genes *Cyp2e1* and *Cyp2f2*. (B) Zonal distribution of mononucleated hepatocytes (2n×1; 4n×1; 8n×1) from the central vein (CV) to the portal vein (PV), and the corresponding density curves. (C) Zonal distribution of binucleated hepatocytes (2n×2; 4n×2) from the CV to the PV, and the corresponding density curves. (D) Zonation gene expression from

the CV to the PV. ANOVA F-test, with all p-values<2e-16. (E) Expression profiles of zonation genes in hepatocyte ploidy subpopulations along the CV–PV axis. Wilcoxon rank-sum test with Benjamini-Hochberg (BH) correction; *p<0.05, **p<0.01, ***p<0.001; ns, no significance.

The transcriptomic profiles analysis of polyploidy hepatocyte

To investigate whether gene expression is correspondingly enhanced with increasing genomic ploidy, we performed comparative transcriptomic profiling of diploid (2c), tetraploid (4c), and octoploid (8c) hepatocytes. Differentially expressed genes (DEGs) were identified by comparing tetraploid vs. diploid, octoploid vs. diploid, and diploid vs. polyploid populations, with significance defined as an adjusted p-value<0.05. We observed that tetraploid cells exhibited fewer upregulated DEGs compared with octoploid cells (**Fig. 5A**), and the upregulated gene set in octoploid hepatocytes encompassed those upregulated in tetraploid cells (**Fig. 5B**). No upregulated DEGs were detected in diploid cells relative to polyploid hepatocytes. Heatmap analysis revealed a gradual increase in expression of these octoploid-associated genes from 2c to 8c (**Fig. 5C**). GO enrichment analysis of the DEGs highlighted terms related to cytoplasmic translation, small molecule catabolism, generation of precursor metabolites and energy, reflecting enhanced metabolic and biosynthetic activity in octoploid hepatocytes (**Fig. 5D**).

Leveraging the advantage of SCIPI, we further explored nuclear count-associated transcriptional changes independent of total DNA content. We found that binucleated hepatocytes displayed more upregulated DEGs than mononucleated cells, both within tetraploid and octoploid populations (**Fig. 5E**). Notably, the upregulated DEGs in both mononucleated and binucleated tetraploid hepatocytes were largely contained within the octoploid DEG set (**Fig. 5F**). GO analysis revealed that tetraploid and octoploid hepatocytes shared enriched pathways such as oxidative phosphorylation, ATP synthesis coupled electron transport, and aerobic respiration, while octoploid cells additionally showed enrichment in small molecule metabolism, organic acid metabolism, and fatty acid metabolism **(Fig. 5G**, supplementary **Fig. S3A–C**). Representative lipid metabolism genes (e.g., *Acox1*, *Apoa1*) and xenobiotic metabolism genes (e.g., *Ces1d*, *Cyp2e1*) confirmed that their expression levels were progressively elevated with increasing ploidy and nuclear number (**Fig. 5H–I**). Hepatic

transcription factors (e.g., *Hnf4a*, *Cebpa*) displayed a similar trend, with their expression increasing progressively with higher ploidy (supplementary **Fig. S3D**).

Fig. 5 Transcriptomic diversity across hepatocyte ploidy subpopulations

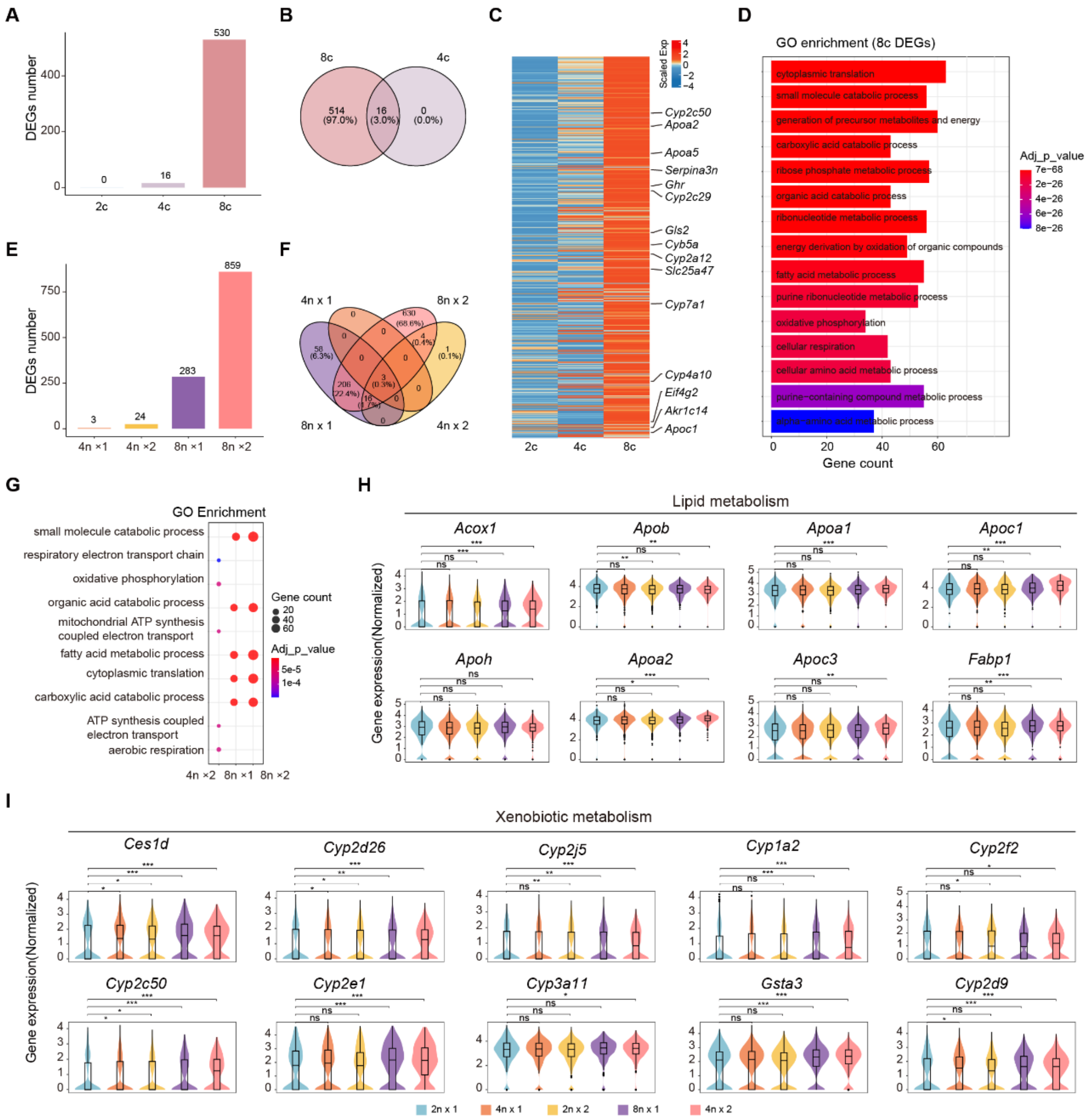

Figure 5 Transcriptomic diversity across hepatocyte ploidy subpopulations. (A) Number of upregulated differentially expressed genes (DEGs) in diploid (2c), tetraploid (4c), and octoploid (8c) cells, identified by adjusted p-value (adj_p_val) < 0.05 and average log2 fold change (avg_log2FC) >0. (B) Venn diagram depicting the overlap of upregulated DEGs between 8c and 4c cells. (C) Heatmap illustrating the scaled expression profiles of upregulated DEGs across 2c, 4c, and 8c cells, with representative genes labeled on the right. (D) Bar plot of Gene Ontology (GO) biological process enrichment analysis for upregulated DEGs in 8c cells. (E) Number of upregulated DEGs in mononuclear tetraploid (4n×1), binuclear tetraploid (2n×2), mononuclear octoploid (8n×1), and binuclear octoploid (4n×2) cells. Adj_p_val < 0.05

and avg_log2FC >0. (F) Venn diagram showing the overlap of upregulated DEGs among 4n×1, 2n×2, 8n×1, and 4n×2 cells. (F) Dot plot of integrated GO enrichment analysis, displaying the top functional pathways of upregulated DEGs in 2n×2, 8n×1, and 4n×2 cells. (H-I) Violin plots showing the normalized expression levels of representative lipid metabolism-related and xenobiotic metabolism-related genes across 2n×1, 4n×1, 2n×2, 8n×1, and 4n×2 cells. Kruskal-Wallis overall test followed by Wilcoxon pairwise tests with Benjamini-Hochberg (BH) correction (ns: not significant; *p < 0.05; **p < 0.01; ***p < 0.001).

Discussion

We developed the Stereo-Cell Image-based Ploidy Identification (SCIPI) method, which reliably classifies hepatocyte ploidy subtypes. In P56 mice, the identified proportions were as follows: 2n×1 (11.4%), 4n×1 (33.9%), 2n×2 (32.3%), 8n×1 (13.0%), 4n×2 (8.1%), and 8n×2 (1.8%). These results showed high concordance with ploidy distributions determined by nuclear area measurement in liver tissue sections and by FACS. During the imaging of FACS-sorted cells, our found the contamination of the sorted diploid cell population with approximately 13% binucleated cells (supplementary **Fig. S1G**). This observation underscores the necessity for stringent gating in future FACS-based studies investigating transcriptional differences across ploidy types.

Our study provides a comprehensive map of transcriptional heterogeneity in polyploid hepatocytes. The proportion of 8n×1 and 4n×2 hepatocytes progressively decreased along the central vein (CV) to portal vein (PV) axis, whereas the proportions of 2n×1 and 2n×2 hepatocytes gradually increased. Transcriptomic analysis revealed that octoploid hepatocytes upregulated a greater number of differentially expressed genes (DEGs) compared to tetraploid cells, relative to diploid counterparts. Comparative analysis between cellular ploidy and nuclear number indicated that binucleated polyploid hepatocytes exhibited more upregulated DEGs than their mononucleated counterparts. The upregulated DEGs in polyploid cells were significantly enriched in metabolic pathways, suggesting that polyploid hepatocytes possess more active metabolic functions.

Compared to FACS-sorted scRNA-seq or snRNA-seq[28,29], SCIPI enables the recovery of a greater number of qualified cells for analysis and achieves higher accuracy in ploidy classification, though it exhibits a relatively lower RNA capture efficiency. Relative to image-based ploidy identification methods like MERFISH[51], SCIPI offers distinct advantages. In tissue sections used for MERFISH, nuclei are three-dimensional and only partially visible, preventing accurate conversion of nuclear area to total DNA content, relying instead on nuclear count to define "multinucleated/mononucleated" status. In contrast, SCIPI dissociates tissue and spreads cells onto a two-dimensional plane, allowing the acquisition of complete, unobstructed nuclear images. By combining bright-field cell contour recognition with DAPI-based nuclear area measurement, it directly correlates nuclear area with DNA content, thereby enabling precise ploidy determination and detailed karyotype analysis of polyploid cells. However, this approach inevitably results in the loss of native tissue microenvironmental information. Furthermore, while MERFISH is limited to a predefined panel of 317 genes focused on markers for hepatocytes and non-parenchymal cells, SCIPI is built on a universal high-throughput sequencing platform, enabling whole-transcriptome analysis. This comprehensive coverage provides a more complete molecular interpretation of functional differences associated with ploidy.

In summary, the SCIPI method provides a novel technical approach for in-depth investigation of the regulatory mechanisms of hepatic polyploidy in liver regeneration, development, and various liver diseases[22,25,26]. This methodological advancement also opens new avenues for studying polyploid cells—such as cardiomyocytes[3,52-54] and skeletal muscle fibers[55-57]—which have been challenging to profile reliably with single-cell transcriptomics.

Methods

Hepatocytes isolation and preparation

Primary hepatocytes were isolated from C57BL/6J 8-week-old male mice using a modified

two-step collagenase perfusion protocol[37-43], in which mouse livers were perfused via the inferior vena cava sequentially with perfusion buffer 0.05 mM EDTA (Thermo, AM9260G) in Ca^{2+}-free HBSS, Gibco, 14175-095) and digestion buffer (0.1 mg/mL collagenase IV, Sigma, C5138-5G; and 1.6 mM $CaCl_2$, Sigma, 793639-500G). The digested liver tissue was transferred to ice-cold DMEM (Cytiva, SH30243.01) for gentle cell dissociation. The cell suspension was sequentially filtered through 70 μm and 40 μm cell strainers to remove residual clusters. The cell suspension was centrifuged at 50×g for 3 min to purify the hepatocytes. The cell suspension was layered over a 50% Percoll solution (Sigma, P7828-500ML) and centrifuged at 100×g for 10 min to isolate viable hepatocytes. The pelleted hepatocytes were resuspended in DMEM, washed twice, and finally resuspended in DPBS (Sigma, D8537-500ML) with 0.04% BSA (Aladdin, A116563-25g). Hepatocytes were counted with a hemocytometer, and cell viability was confirmed to be >96%. Hepatocytes were immediately processed for Stereo-cell sequencing to prevent cell death associated with prolonged storage (≤ 1 h post-isolation at 4 °C).

Stereo chip preprocessing for hepatocyte loading and microimaging

The manufacturer's kits were used (Stereo-seq Spatiotemporal Single Cell Transcriptome Reagent Kit, STOmics, STC1000R; Stereo-seq Spatiotemporal Single Cell Barcode Amplification Reagent Kit, STOmics, STC1000P), and the experimental procedures were performed according to the previously reported protocol[33]. The Stereo chip had been placed in a 24-well plate was washed twice with nuclease-free water. Transfer the chip to a new well, coat it with 400 μL of 0.01% poly-L-lysine solution, and incubate it on an orbital shaker (50-250 rpm) at room temperature for one hour. After aspiration of the coating solution, the chip was rinsed twice with nuclease-free water and moved to a clean well. After aspiration of the coating solution, the chip was rinsed twice with nuclease-free water and moved to a clean well. A suspension of hepatocytes in 20 μL of DPBS containing 0.04% BSA was carefully applied to the chip surface. It is critical to gently spread the suspension with a pipette tip for even cell distribution while avoiding direct contact with the chip surface. The chip was incubated at room temperature for 15 min to allow cell attachment, followed by fixation in ice-cold methanol (-20 °C) for 30 min. Fixed cells are subsequently stained with DAPI solution (Thermo, 62248),

with systematic exclusion of glycerol in mounting protocols to prevent hydrodynamic displacement of large polyploid hepatocytes during high-resolution imaging. Final micrographs are acquired using a Motic PA53 FS6 microscope operating synchronized bright-field and DAPI fluorescence channels, ensuring co-registered.

Library construction and sequencing

The Stereo-cell library construction for hepatocytes refers to Liao et al in 2025[33]. After imaging, hepatocytes were permeabilized with 0.1% pepsin at 37 °C for 30 s. RNA released from the permeabilized cells was captured by the chip's DNA Nanoballs (DNBs) and subsequently reverse transcribed overnight at 42 °C. Chips containing cDNA were then treated overnight at 55 °C for 3 h with a cDNA release mixture. The released cDNA was collected and amplified using sequencing sample barcode primers to generate Stereo-cell libraries. These libraries were quantified using the Qubit dsDNA Assay Kit (Thermo, Q32854) and either the Agilent 2100 Bioanalyzer or Qsep, respectively. 80 ng of DNA samples was used as input for DNA nanoball (DNB) generation. The DNB library was sequenced on an MGI DNBSEQ-Tx platform using ~50 bp paired-end sequencing.

Immunohistochemistry and analysis

For liver frozen sections, all experiments were performed using the BeyoTSA™ Triple-Label Four-Color Multiplex Fluorescent Staining Kit (Beyotime, P1351S). Samples were fixed and permeabilized separately at room temperature (RT). Endogenous peroxidase was blocked with blocking solution (P0100A) at RT for 10–15 min. Nonspecific background was blocked with appropriate blocking buffer (P0102) at RT. β-Catenin antibody (Cell Signaling, 9582) was diluted properly and incubated with samples at RT for 2 h. Corresponding secondary antibody was diluted and incubated at RT for 60 min. TSA staining working solution was added for 10 min at RT in the dark. Subsequently, DAPI staining solution was added for nuclear counterstaining at RT in the dark for 5 min. Staining results were observed under a fluorescence microscope. After completing microscopic imaging, three fields of view were randomly

selected for each mouse replicate. Following the same workflow as Stereo-cell (without the need for registration), we performed cell segmentation on β-Catenin fluorescence images and nuclear segmentation on DAPI fluorescence images, respectively. Subsequently, ploidy identification was conducted using the identical method, and the proportions of different ploidy types were quantified separately.

Flow cytometry and analysis

For hepatocyte flow cytometry sorting, 1–2×10^6 prepared hepatocyte suspension was adjusted to 500 µL and placed in a 15 mL centrifuge tube. Ice-cold absolute ethanol was slowly added to 2 mL (final concentration 75%), and the cells were fixed at 4 °C for 60 min. After centrifugation at 100×g for 5 min, the supernatant was discarded. Then 1 mL of 1×PBS (Gibco, 10010001) was added to resuspend the cells, followed by two additional centrifugation washes. The supernatant was discarded, and 2 µg/mL DAPI staining solution was added; after mixing, the cells were stained at 4 °C for 20 min in the dark. Finally, the cells were washed twice with 1×PBS, filtered through a 40 µm filter, and hepatocyte populations with different fluorescence intensities were sorted by flow cytometry. For each ploidy type (2c, 4c, 8c) across different batches, cells were evenly spread onto glass slides and imaged under a microscope. Sorting was performed with imaging via the BASIC channel and DAPI channel. Ultimately, cell images and nuclear images for each ploidy type were acquired. Subsequently, we completed cell segmentation and assign nuclei to their corresponding cells. This process yielded *_nucle_info.csv files, which contain metrics such as the number of nuclei per cell and nuclear area. Cells with fewer than 1 or more than 2 nuclei were first filtered out, and the average nuclear area was then calculated. By integrating the mean fluorescence intensity of different ploidy populations previously determined via flow cytometry, the correlation between nuclear area and DNA content can be directly assessed.

Raw sequencing data preprocessing

After sequencing, fastq format files will be obtained, which contain DNA base sequences, spatial coordinate sequences, and unique molecular identifier (UMI) sequences， after

processing with SAW[44], we can obtain GEM-format files that have undergone sequence demultiplexing and alignment. This file contains Gene IDs, gene coordinates (x, y), molecular identifier count (MID Count), and exon counts. Based on this information, we can generate in situ UMI counts image (UMI image) with script gem2mask.py.

Image registration and preprocessing

Image registration was performed using FIJI ImageJ[58]. Since the microscope stage remained stationary during sequential imaging, the brightfield and DAPI images were inherently aligned. Thus, ImageJ was solely used to register the DAPI image with the corresponding UMI image. Both DAPI and brightfield images were converted from RGB to 8-bit to reduce memory usage, and brightness was adjusted to achieve consistency between the DAPI and UMI signals. A TrackEM2 project was created, and the folder containing the brightfield, DAPI, and UMI images was imported. Switch the active image to the UMI image. Unlink the UMI image from other images to ensure that adjustments to the DAPI image do not affect the UMI image. Maintain the link between the DAPI and brightfield images. Select the DAPI layer as the active layer. Configure the UMI image as the red channel and DAPI as the green channel in the layer settings. Register the DAPI image using the Transform tool, after registration, validate the alignment by zooming into multiple fields of view to ensure that the majority of the DAPI signal (green channel) overlaps with the UMI regions (red channel). Export and save both the DAPI and brightfield images in TIFF format.

Cell segmentation and ploidy analysis

Perform cell segmentation on both the DAPI and brightfield images that have been co-registered with the UMI image. For the brightfield image, use Cellpose with the cyto3 model[45]. Typically, first run the software to determine the diameter using the calibrate option for automatic assessment. Save the results as PNG-format images. For the DAPI image, use StarDist with the 2D_versatile_fluo model[59] to generate DAPI masks. Save the output as CSV-format files. Next, identify hepatocytes polyploidy. First, perform cell segmentation on the GEM file using the results from Cellpose. The processed GEM file will include an

additional mask column, where different mask values represent individual cells. Calculate the correspondence between DAPI masks and cell segmentation results. We define that if ≥50% of a DAPI mask's area overlaps with a cell region, the DAPI mask is assigned to that cell. Record the correspondence between DAPI masks and cell masks, DAPI areas, DAPI counts per cell, and other relevant nuclear information in a new DataFrame, which is then saved. Generate a single-cell matrix file from the new GEM file, which can then be imported into Seurat or Scanpy for downstream analysis. Nuclear information is incorporated into the metadata.

Pseudotime analysis of hepatic zonation

To construct the pseudotime of liver zonation, we utilized the AddModuleScore function in Seurat software. Specifically, we defined two gene modules: the central vein (CV) module consisting of (*Cyp2e1*, *Glul*, *Cyp1a2*, *Cyb5a*, *Cyp2a5*, *Cyp27a1*) and the periportal (PV) module consisting of (*Cyp2f2*, *Cdh1*, *Hal*, *Sds*, *Aldob*, *Gls2*). Subsequently, we calculated the Zonation Score as the difference between the PV score and the CV score (PV score − CV score). This Zonation Score was then evenly divided into 20 bins to generate the pseudotime score for liver zonation.

Data Availability

Raw sequencing data supporting the findings of this study have been deposited in the CNGB Nucleotide Sequence Archive (CNSA) under accession number CNP0008257. Supporting data, including processed data, imaging data, and code used to generate figures, are available in the GigaDB[60].

Availability of Source Code and Requirements

Project name: Hep_Ploidy_protocol

Project homepage: https://github.com/JeffLuo9/Hep_Ploidy_protocol

License: MIT license

Operating system: CentOS Stream 10 (Linux 6.12.0-126.el10.x86_64); Windows 11

Programming language: Python; R; Shell.

Package management: Conda; pip (Python); Bioconductor/CRAN (R)

Hardware requirements: Minimum: 64 GB RAM, 4-core CPU, 256 GB SSD; Recommended: 64 GB RAM, 16-core CPU, NVIDIA RTX 5090 GPU, 1 TB SSD

RRID: SCR_027937

BioTools ID: scipi

Ethics approval

All relevant animal experimental procedures described in this study comply with ethical regulations for animal research and were performed under the approval of the Institute of Review Board of Bioethics and Biosafety of BGI (BGI-IRB), with the license number BGI-IRB A24013-T1.

Acknowledgements

We thank all team members of the Stereo-cell R&D group, with special acknowledgements to Xiaoxi Zhou, Hongyu Luo, Chang Liu, Yaling Huang, and Xuerong Li, for their robust support and valuable technical assistance. We also thank Zhi Huang for his help with code development and optimization. All project analyses were performed using the STOmic Cloud platform.

Author contributions

S.H. conceived and supervised this study, and designed the original analytical strategy for hepatocyte ploidy classification. Y.Y. implemented and optimized the Stereo-cell experimental protocol with assistance from J.L., Y.C., P.G., Q.G., H.W. and Q.L.. J.L. developed the computational pipeline and conducted most of the data analyses with assistance from Y.Y.. Y.Y. and J.L. drafted the manuscript, with input from all authors.

Competing interests

The authors declare no competing interests.

Funding

This work was supported by the Zhejiang Provincial Natural Science Foundation of China (Grant No. LMS26C060001); the National Natural Science Foundation of China (Grant No. 32500590); and the China National Postdoctoral Program for Innovative Talents (Grant No. BX20250145).

Reference

1 Rios, A. C., Fu, N. Y., Jamieson, P. R. *et al.* Essential role for a novel population of binucleated mammary epithelial cells in lactation. *Nat Commun* **7**, 11400 (2016). https://doi.org/10.1038/ncomms11400

2 Unhavaithaya, Y. & Orr-Weaver, T. L. Polyploidization of glia in neural development links tissue growth to blood-brain barrier integrity. *Genes Dev* **26**, 31-36 (2012). https://doi.org/10.1101/gad.177436.111

3 Anatskaya, O. V. & Vinogradov, A. E. Genome multiplication as adaptation to tissue survival: evidence from gene expression in mammalian heart and liver. *Genomics* **89**, 70-80 (2007). https://doi.org/10.1016/j.ygeno.2006.08.014

4 Zanet, J., Freije, A., Ruiz, M. *et al.* A mitosis block links active cell cycle with human epidermal differentiation and results in endoreplication. *PLoS One* **5**, e15701 (2010). https://doi.org/10.1371/journal.pone.0015701

5 Sher, N., Von Stetina, J. R., Bell, G. W. *et al.* Fundamental differences in endoreplication in mammals and Drosophila revealed by analysis of endocycling and endomitotic cells. *Proc Natl Acad Sci U S A* **110**, 9368-9373 (2013). https://doi.org/10.1073/pnas.1304889110

6 Anatskaya, O. V., Vinogradov, A. E. & Kudryavtsev, B. N. Hepatocyte polyploidy and metabolism/life-history traits: hypotheses testing. *J Theor Biol* **168**, 191-199 (1994). https://doi.org/10.1006/jtbi.1994.1098

7 Gentric, G. & Desdouets, C. Liver polyploidy: Dr Jekyll or Mr Hide? *Oncotarget* **6**, 8430-8431 (2015). https://doi.org/10.18632/oncotarget.3809

8 Wang, M. J., Chen, F., Lau, J. T. Y. & Hu, Y. P. Hepatocyte polyploidization and its association with pathophysiological processes. *Cell Death Dis* **8**, e2805 (2017). https://doi.org/10.1038/cddis.2017.167

9 Margall-Ducos, G., Celton-Morizur, S., Couton, D., Brégerie, O. & Desdouets, C. Liver tetraploidization is controlled by a new process of incomplete cytokinesis. *J Cell Sci* **120**, 3633-3639 (2007). https://doi.org/10.1242/jcs.016907

10 Fang, J., de Bruin, A., Villunger, A. *et al.* Cellular polyploidy in organ homeostasis and regeneration. *Protein Cell* **14**, 560-578 (2023). https://doi.org/10.1093/procel/pwac064

11 Fortier, M., Celton-Morizur, S. & Desdouets, C. Incomplete cytokinesis/binucleation in mammals: The powerful system of hepatocytes. *Methods Cell Biol* **137**, 119-142 (2017). https://doi.org/10.1016/bs.mcb.2016.04.006

12 Donne, R., Saroul-Aïnama, M., Cordier, P., Celton-Morizur, S. & Desdouets, C. Polyploidy in liver development, homeostasis and disease. *Nat Rev Gastroenterol Hepatol* **17**, 391-405 (2020). https://doi.org/10.1038/s41575-020-0284-x

13 Guidotti, J. E., Brégerie, O., Robert, A. *et al.* Liver cell polyploidization: a pivotal role for binuclear hepatocytes. *J Biol Chem* **278**, 19095-19101 (2003). https://doi.org/10.1074/jbc.M300982200

14 Celton-Morizur, S., Merlen, G., Couton, D., Margall-Ducos, G. & Desdouets, C. The insulin/Akt pathway controls a specific cell division program that leads to generation of binucleated tetraploid liver cells in rodents. *J Clin Invest* **119**, 1880-1887 (2009). https://doi.org/10.1172/jci38677

15 Chao, H. W., Doi, M., Fustin, J. M. *et al.* Circadian clock regulates hepatic polyploidy by modulating Mkp1-Erk1/2 signaling pathway. *Nat Commun* **8**, 2238 (2017). https://doi.org/10.1038/s41467-017-02207-7

16 Pandit, S. K., Westendorp, B., Nantasanti, S. *et al.* E2F8 is essential for polyploidization in mammalian cells. *Nat Cell Biol* **14**, 1181-1191 (2012). https://doi.org/10.1038/ncb2585

17 Chen, H. Z., Ouseph, M. M., Li, J. *et al.* Canonical and atypical E2Fs regulate the mammalian endocycle. *Nat Cell Biol* **14**, 1192-1202 (2012). https://doi.org/10.1038/ncb2595

18 Wilkinson, P. D., Delgado, E. R., Alencastro, F. *et al.* The Polyploid State Restricts Hepatocyte Proliferation and Liver Regeneration in Mice. *Hepatology* **69**, 1242-1258 (2019). https://doi.org/10.1002/hep.30286

19 Wang, M. J., Chen, F., Li, J. X. *et al.* Reversal of hepatocyte senescence after continuous in vivo cell proliferation. *Hepatology* **60**, 349-361 (2014). https://doi.org/10.1002/hep.27094

20 Gorla, G. R., Malhi, H. & Gupta, S. Polyploidy associated with oxidative injury attenuates proliferative potential of cells. *J Cell Sci* **114**, 2943-2951 (2001). https://doi.org/10.1242/jcs.114.16.2943

21 Malhi, H., Gorla, G. R., Irani, A. N., Annamaneni, P. & Gupta, S. Cell transplantation after oxidative hepatic preconditioning with radiation and ischemia-reperfusion leads to extensive liver repopulation. *Proc Natl Acad Sci U S A* **99**, 13114-13119 (2002). https://doi.org/10.1073/pnas.192365499

22 Gentric, G., Maillet, V., Paradis, V. *et al.* Oxidative stress promotes pathologic polyploidization in nonalcoholic fatty liver disease. *J Clin Invest* **125**, 981-992 (2015). https://doi.org/10.1172/jci73957

23 Yin, K., Büttner, M., Deligiannis, I. K. *et al.* Polyploidisation pleiotropically buffers ageing in hepatocytes. *J Hepatol* **81**, 289-302 (2024). https://doi.org/10.1016/j.jhep.2024.03.043

24 Zhang, S., Zhou, K., Luo, X. *et al.* The Polyploid State Plays a Tumor-Suppressive Role in the Liver. *Dev Cell* **44**, 447-459.e445 (2018). https://doi.org/10.1016/j.devcel.2018.01.010

25 Bu, W., Sun, X., Xue, X. *et al.* Early onset of pathological polyploidization and cellular senescence in hepatocytes lacking RAD51 creates a pro-fibrotic and pro-tumorigenic inflammatory microenvironment. *Hepatology* **81**, 491-508 (2025). https://doi.org/10.1097/hep.0000000000000821

26 Wang, J., Huang, X., Zheng, D. *et al.* PRMT5 determines the pattern of polyploidization and prevents liver from cirrhosis and carcinogenesis. *J Genet Genomics* **50**, 87-98 (2023). https://doi.org/10.1016/j.jgg.2022.04.008

27 Tanami, S., Ben-Moshe, S., Elkayam, A. *et al.* Dynamic zonation of liver polyploidy. *Cell Tissue Res* **368**, 405-410 (2017). https://doi.org/10.1007/s00441-016-2427-5

28 Richter, M. L., Deligiannis, I. K., Yin, K. *et al.* Single-nucleus RNA-seq2 reveals functional crosstalk between liver zonation and ploidy. *Nat Commun* **12**, 4264 (2021). https://doi.org/10.1038/s41467-021-24543-5

29 Yang, L., Wang, X., Zheng, J. X. *et al.* Determination of key events in mouse hepatocyte maturation at the single-cell level. *Dev Cell* **58**, 1996-2010.e1996 (2023). https://doi.org/10.1016/j.devcel.2023.07.006

30 Wei, X., Fu, S., Li, H. *et al.* Single-cell Stereo-seq reveals induced progenitor cells involved in axolotl brain regeneration. *Science* **377**, eabp9444 (2022). https://doi.org/10.1126/science.abp9444

31 Chen, A., Liao, S., Cheng, M. *et al.* Spatiotemporal transcriptomic atlas of mouse organogenesis using DNA nanoball-patterned arrays. *Cell* **185**, 1777-1792.e1721 (2022). https://doi.org/10.1016/j.cell.2022.04.003

32 Xu, J., Guo, P., Hao, S. *et al.* A spatiotemporal atlas of mouse liver homeostasis and regeneration. *Nat Genet* **56**, 953-969 (2024). https://doi.org/10.1038/s41588-024-01709-7

33 Liao, S., Zhou, X., Liu, C. *et al.* Stereo-cell: Spatial enhanced-resolution single-cell sequencing with high-density DNA nanoball-patterned arrays. *Science* **389** (2025). https://doi.org/10.1126/science.adr0475

34 Zhou, L., Peng, X., Chen, M. *et al.* Unveiling patterns in spatial transcriptomics data: a novel approach utilizing graph attention autoencoder and multiscale deep subspace clustering network. *GigaScience* **14** (2025). https://doi.org/10.1093/gigascience/giae103

35 Liu, X., Qu, C., Liu, C. *et al.* StereoSiTE: a framework to spatially and quantitatively profile the cellular neighborhood organized iTME. *GigaScience* **13** (2024). https://doi.org/10.1093/gigascience/giae078

36 Jiahui, L., Shijie, H., Yongqing, Y. & Zhi, H. SCIPI: a Tool for hepatocyte ploidy subpopulation identification. (2026). https://doi.org/10.48546/WORKFLOWHUB.WORKFLOW.2079.3

37 Berry, M. N. & Friend, D. S. High-yield preparation of isolated rat liver parenchymal cells: a biochemical and fine structural study. *J Cell Biol* **43**, 506-520 (1969). https://doi.org/10.1083/jcb.43.3.506

38 MacParland, S. A., Liu, J. C., Ma, X. Z. *et al.* Single cell RNA sequencing of human liver reveals distinct intrahepatic macrophage populations. *Nat Commun* **9**, 4383 (2018). https://doi.org/10.1038/s41467-018-06318-7

39 Casciano, D. A. Development and utilization of primary hepatocyte culture systems to evaluate metabolism, DNA binding, and DNA repair of xenobiotics. *Drug Metab Rev* **32**, 1-13 (2000). https://doi.org/10.1081/dmr-100100561

40 Klaunig, J. E., Goldblatt, P. J., Hinton, D. E. *et al.* Mouse liver cell culture. I. Hepatocyte isolation. *In Vitro* **17**, 913-925 (1981). https://doi.org/10.1007/bf02618288

41 Li, W. C., Ralphs, K. L. & Tosh, D. Isolation and culture of adult mouse hepatocytes. *Methods Mol Biol* **633**, 185-196 (2010). https://doi.org/10.1007/978-1-59745-019-5_13

42 Severgnini, M., Sherman, J., Sehgal, A. *et al.* A rapid two-step method for isolation of

functional primary mouse hepatocytes: cell characterization and asialoglycoprotein receptor based assay development. *Cytotechnology* **64**, 187-195 (2012). https://doi.org/10.1007/s10616-011-9407-0

43 Charni-Natan, M. & Goldstein, I. Protocol for Primary Mouse Hepatocyte Isolation. *STAR Protoc* **1**, 100086 (2020). https://doi.org/10.1016/j.xpro.2020.100086

44 Gong, C., Li, S., Wang, L. *et al.* SAW: an efficient and accurate data analysis workflow for Stereo-seq spatial transcriptomics. *GigaByte* **2024**, gigabyte111 (2024). https://doi.org/10.46471/gigabyte.111

45 Stringer, C., Wang, T., Michaelos, M. & Pachitariu, M. Cellpose: a generalist algorithm for cellular segmentation. *Nat Methods* **18**, 100-106 (2021). https://doi.org/10.1038/s41592-020-01018-x

46 Saeter, G., Lee, C. Z., Schwarze, P. E. *et al.* Changes in ploidy distributions in human liver carcinogenesis. *J Natl Cancer Inst* **80**, 1480-1485 (1988). https://doi.org/10.1093/jnci/80.18.1480

47 Duncan, A. W., Taylor, M. H., Hickey, R. D. *et al.* The ploidy conveyor of mature hepatocytes as a source of genetic variation. *Nature* **467**, 707-710 (2010). https://doi.org/10.1038/nature09414

48 Duncan, A. W., Hanlon Newell, A. E., Smith, L. *et al.* Frequent aneuploidy among normal human hepatocytes. *Gastroenterology* **142**, 25-28 (2012). https://doi.org/10.1053/j.gastro.2011.10.029

49 Halpern, K. B., Shenhav, R., Matcovitch-Natan, O. *et al.* Single-cell spatial reconstruction reveals global division of labour in the mammalian liver. *Nature* **542**, 352-356 (2017). https://doi.org/10.1038/nature21065

50 Ben-Moshe, S. & Itzkovitz, S. Spatial heterogeneity in the mammalian liver. *Nat Rev Gastroenterol Hepatol* **16**, 395-410 (2019). https://doi.org/10.1038/s41575-019-0134-x

51 Watson, B. R., Paul, B., Rahman, R. U. *et al.* Spatial transcriptomics of healthy and fibrotic human liver at single-cell resolution. *Nat Commun* **16**, 319 (2025). https://doi.org/10.1038/s41467-024-55325-4

52 Bensley, J. G., De Matteo, R., Harding, R. & Black, M. J. Three-dimensional direct measurement of cardiomyocyte volume, nuclearity, and ploidy in thick histological sections. *Sci Rep* **6**, 23756 (2016). https://doi.org/10.1038/srep23756

53 Østergaard, K. H., Baandrup, U. T., Wang, T. *et al.* Left ventricular morphology of the giraffe heart examined by stereological methods. *Anat Rec (Hoboken)* **296**, 611-621 (2013). https://doi.org/10.1002/ar.22672

54 Hirose, K., Payumo, A. Y., Cutie, S. *et al.* Evidence for hormonal control of heart regenerative capacity during endothermy acquisition. *Science* **364**, 184-188 (2019). https://doi.org/10.1126/science.aar2038

55 Millay, D. P. Regulation of the myoblast fusion reaction for muscle development, regeneration, and adaptations. *Exp Cell Res* **415**, 113134 (2022). https://doi.org/10.1016/j.yexcr.2022.113134

56 Petrany, M. J., Swoboda, C. O., Sun, C. *et al.* Single-nucleus RNA-seq identifies transcriptional heterogeneity in multinucleated skeletal myofibers. *Nat Commun* **11**, 6374 (2020). https://doi.org/10.1038/s41467-020-20063-w

57 Sun, C., Swoboda, C. O., Morales, F. M. *et al.* Lineage tracing of nuclei in skeletal myofibers uncovers distinct transcripts and interplay between myonuclear populations. *Nat Commun* **15**, 9372 (2024). https://doi.org/10.1038/s41467-024-53510-z

58 Schindelin, J., Arganda-Carreras, I., Frise, E. *et al.* Fiji: an open-source platform for biological-

image analysis. *Nat Methods* **9**, 676-682 (2012). https://doi.org/10.1038/nmeth.2019
59 Schmidt, U., Weigert, M., Broaddus, C. & Myers, G. Medical Image Computing and Computer Assisted Intervention – MICCAI 2018 11071, 265 – 273 (2018). https://doi.org/10.1007/978-3-030-00934-2_30
60 Yongqing, Y., Jiahui, L., Yier, C. *et al.* Stereo-cell deciphers the spatial and functional heterogeneity of polyploid hepatocytes. *GigaScience Database* (2026). https://doi.org/10.5524/102774

Fig1

Click here to access/download;Figure;GS_Fig1_revised_260226.pdf

Fig. 1 Comprehensive Stereo-cell experimental workflow for hepatocytes

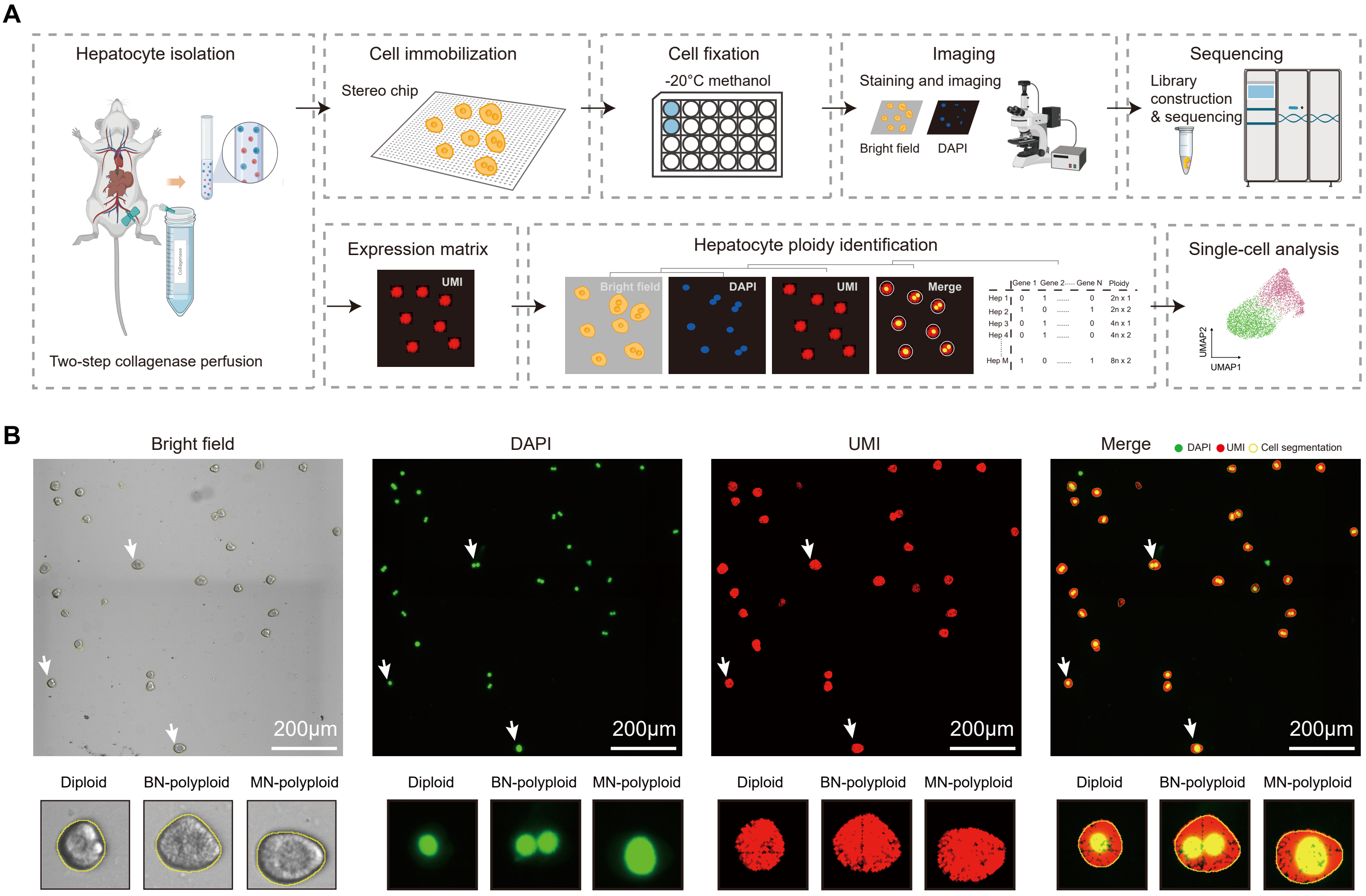

Click here to access/download;Figure;GS_Fig2_revised_260226.pdf

Fig. 2 SCIPI pipeline for spatially resolved hepatocyte ploidy profiling

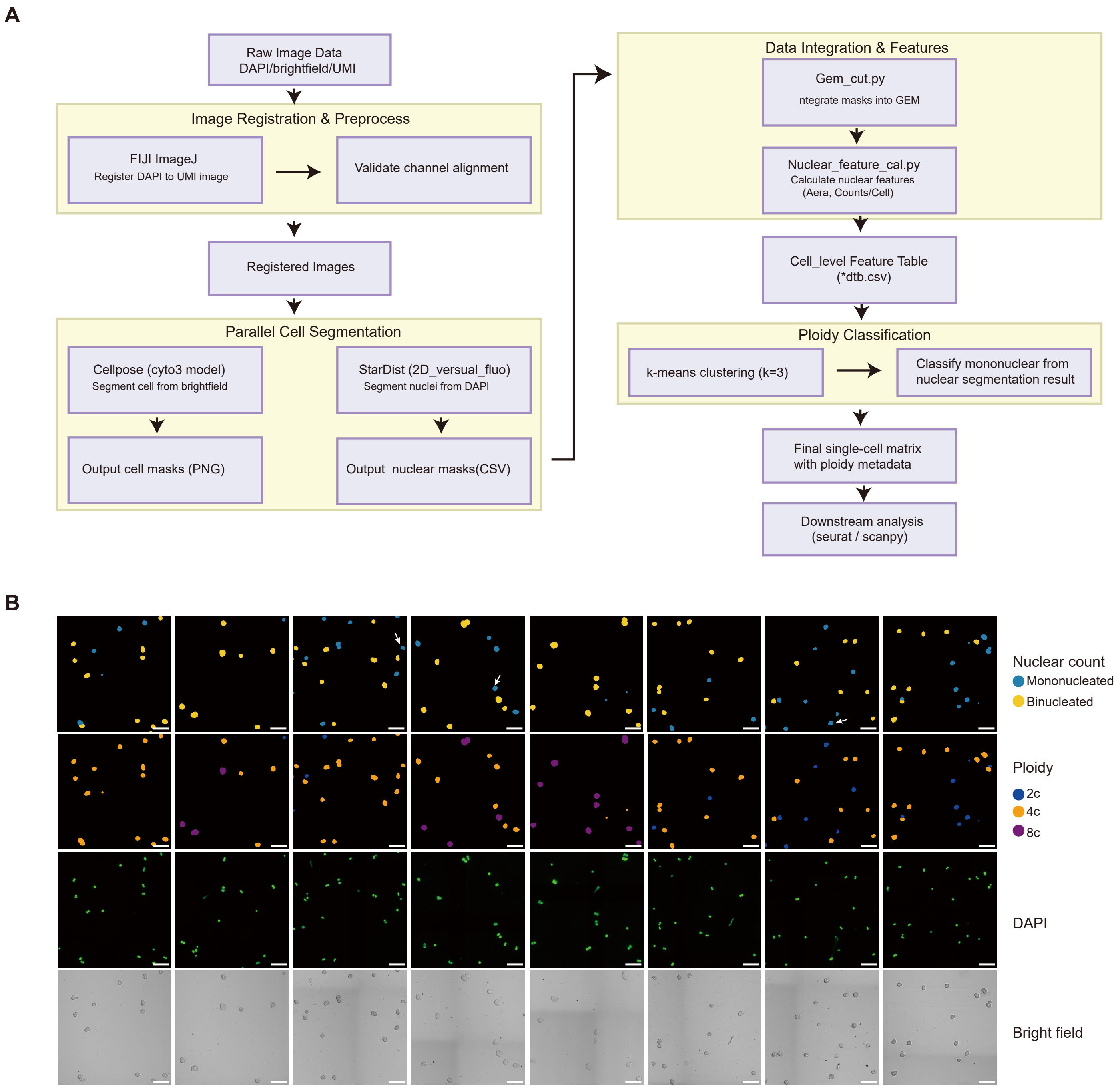

Fig3

Click here to access/download;Figure;GS_Fig3_revised_260226.pdf

Fig. 3 Quantifying and classifying hepatocyte polyploidy

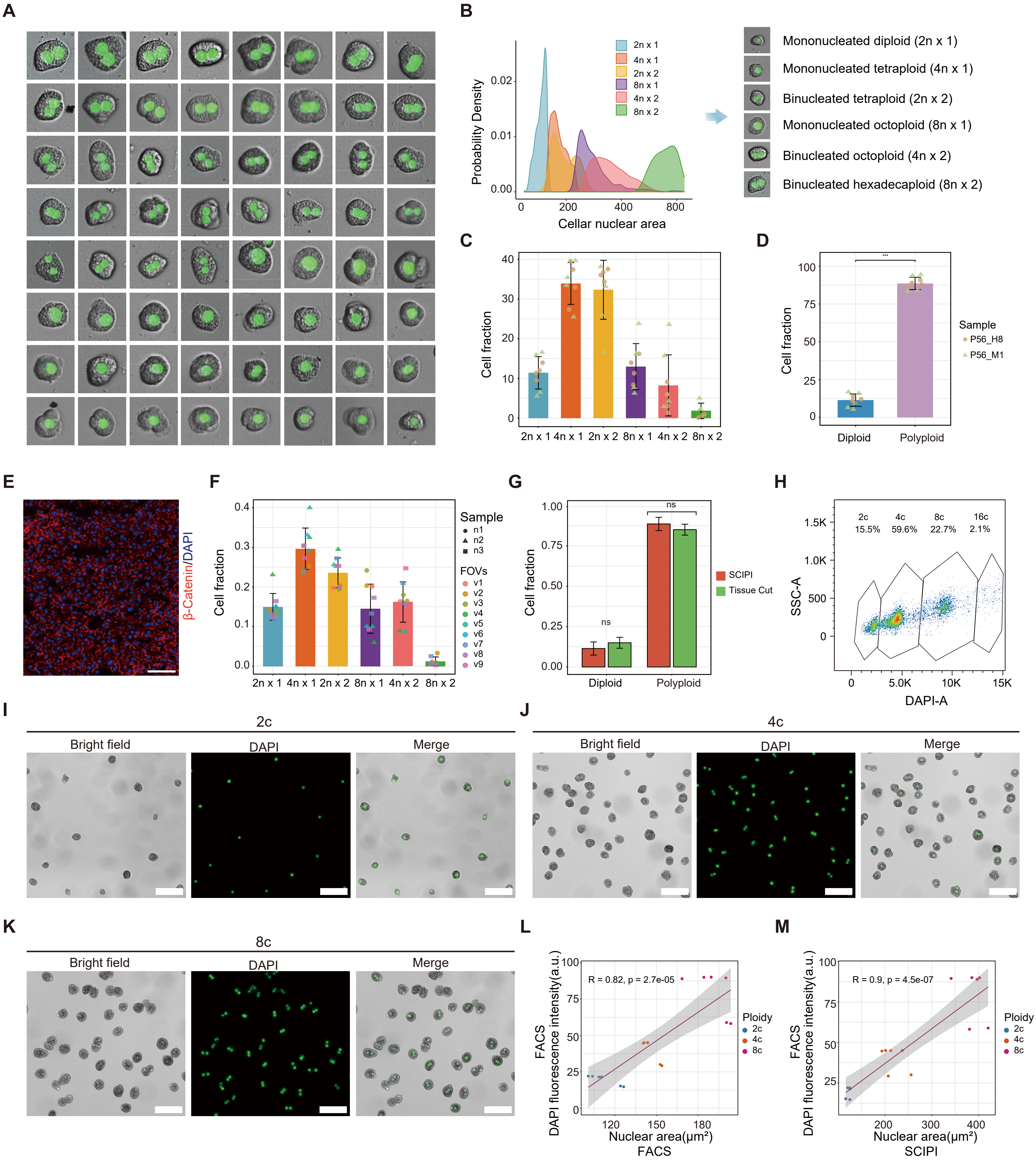

fig4
Click here to access/download;Figure;GS_fig4_revised_260226.pdf

Fig. 4 Gene expression along CV to PV trajectory by ploidy

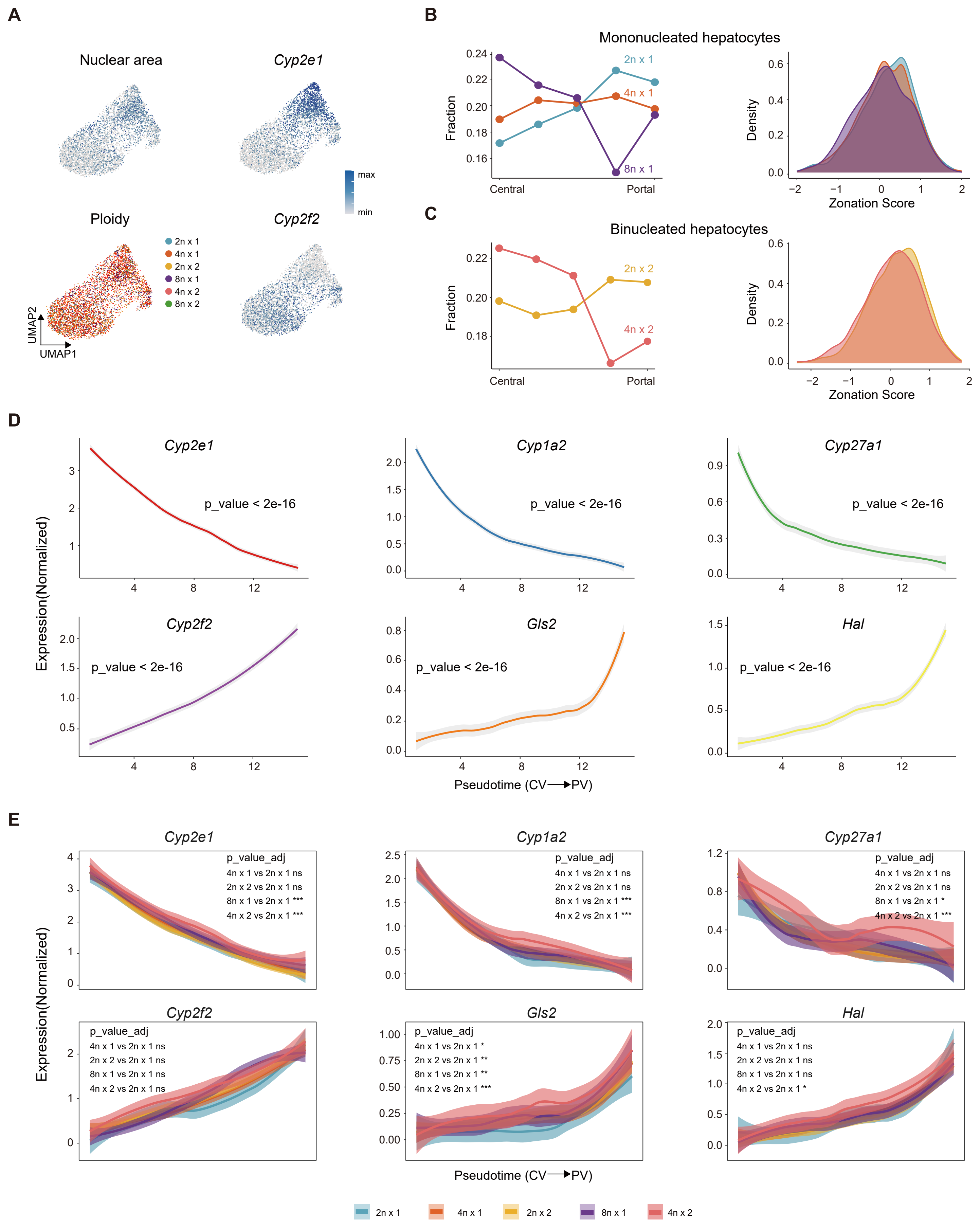

Fig5

Click here to access/download;Figure;GS_Fig5_revised_260226.pdf

Fig. 5 Transcriptomic diversity across hepatocyte ploidy subpopulations

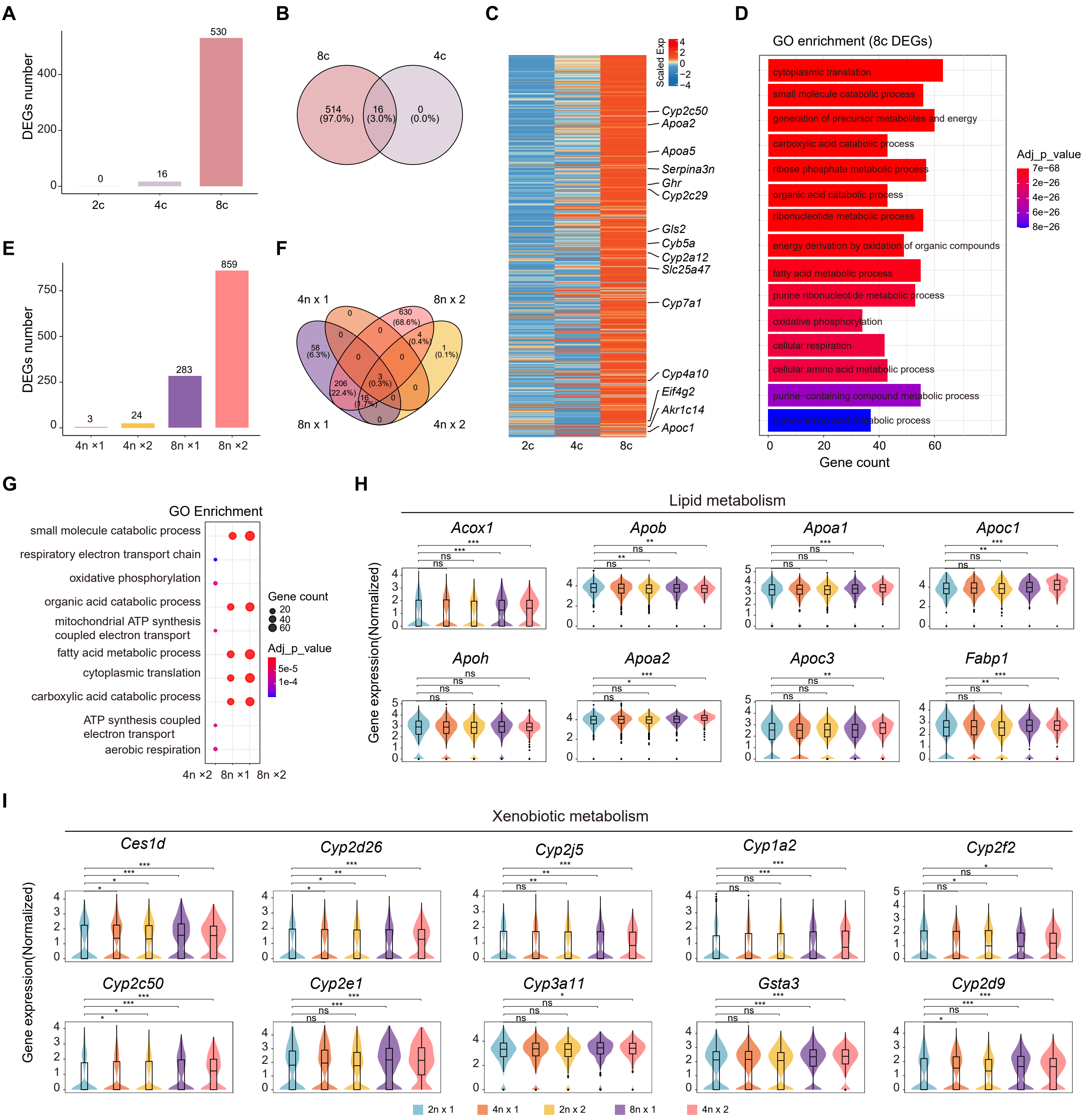

Click here to access/download;Figure;Supplementary_Fig1_260226.pdf

Supplementary Fig. 1 Flow cytometric DNA content analysis combined with nuclear area assessment

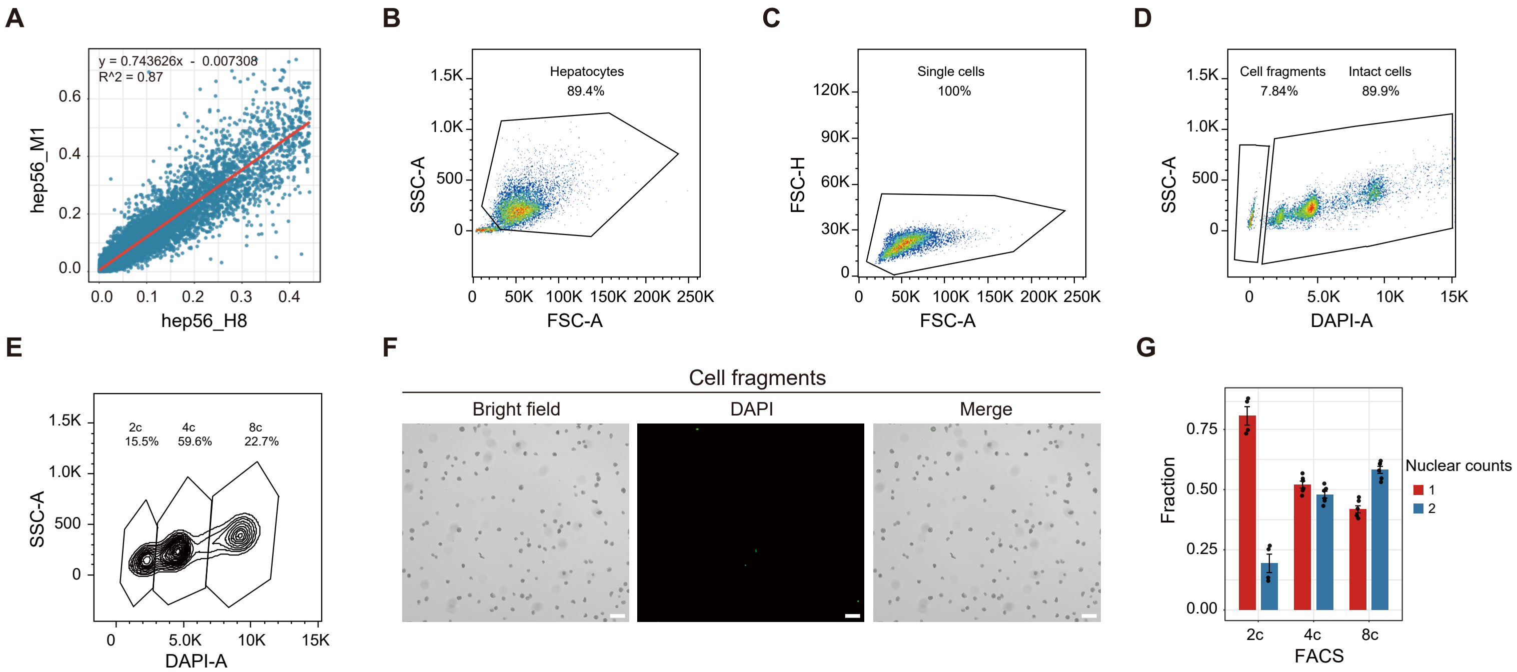

Click here to access/download;Figure;Supplementary_Fig2_260226.pdf

Supplementary Fig. 2 Integrated analysis of stereo-cell data and published scRNA-seq datasets

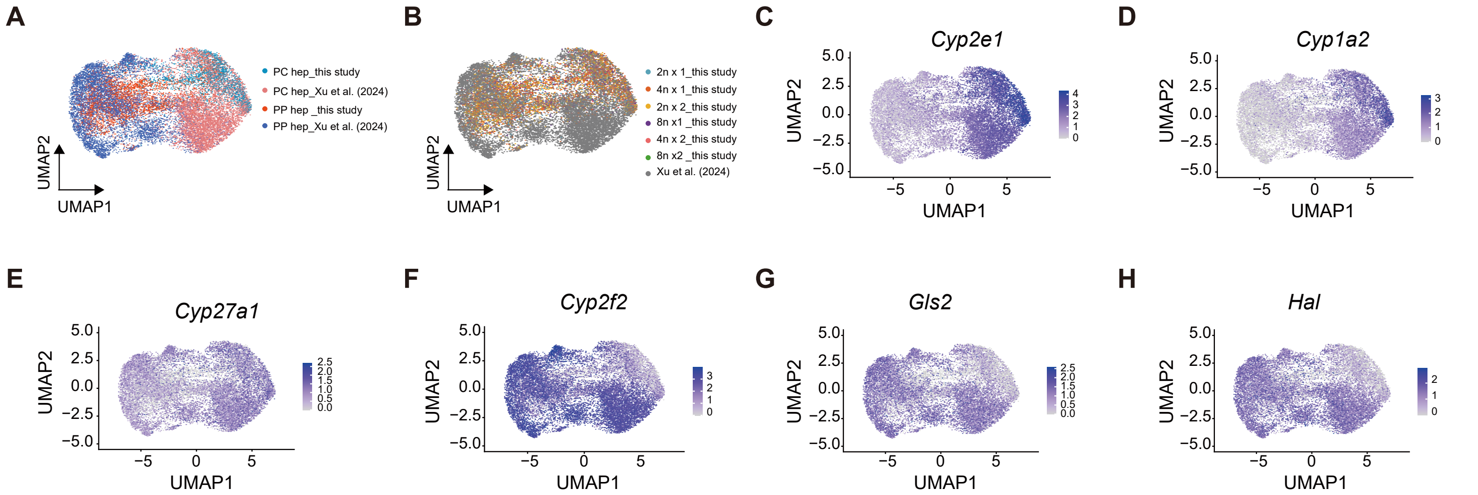

Click here to access/download;Figure;Supplementary_Fig3_260226.pdf

Supplementary Fig. 3 GO enrichment analysis for the specific effects of nuclear and cellular ploidy

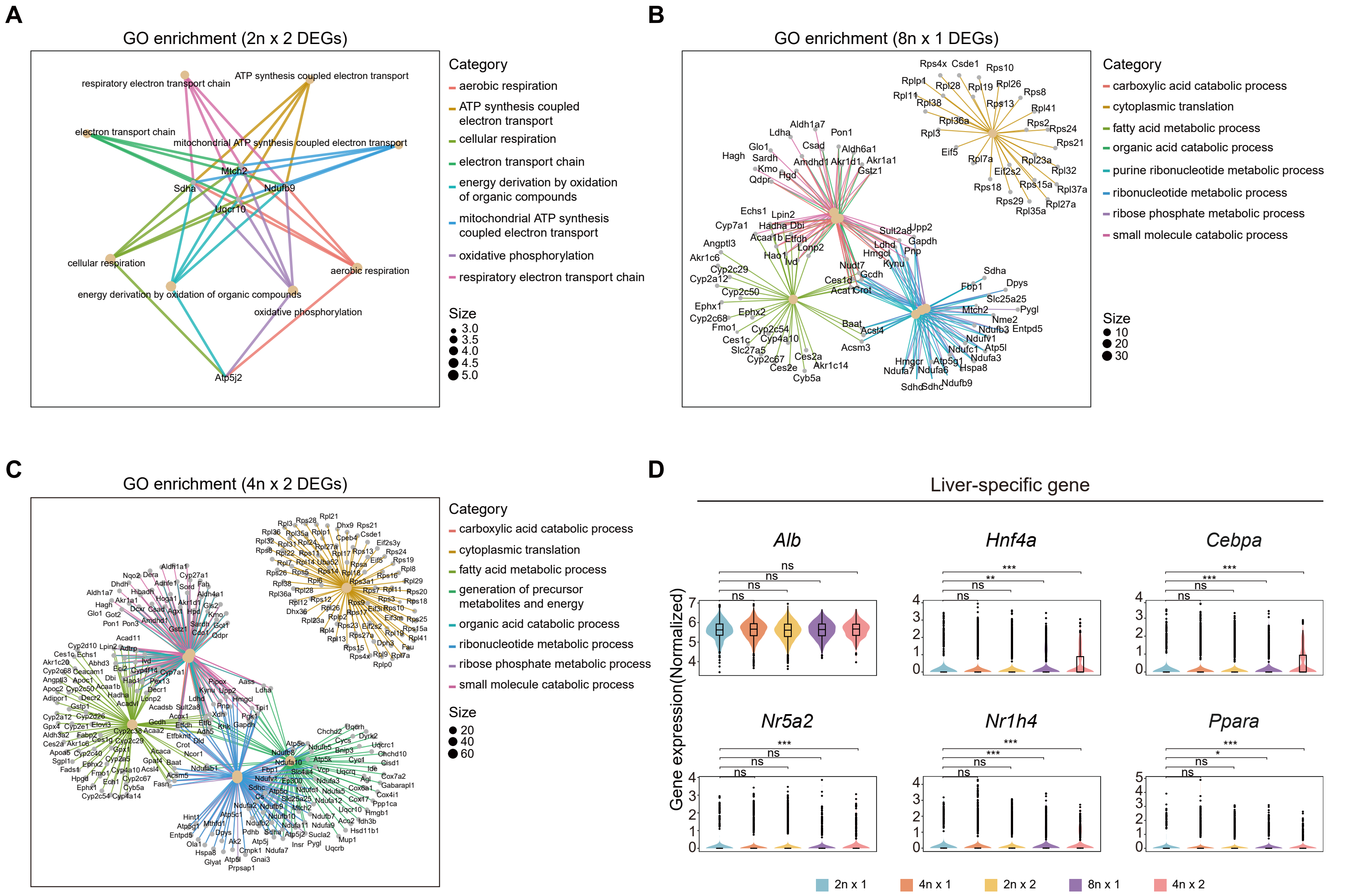

Proposed cover image

Click here to access/download
Supplementary Material
cover_design_A.psd

Proposed cover image

Click here to access/download
Supplementary Material
cover_design_A.jpg

Click here to access/download
Supplementary Material
Supplementary figure legend_260211.docx

Click here to access/download
Supplementary Material
Rebuttal letter to Reviewers comments_260211.docx

cover letter

Click here to access/download
Supplementary Material
GS_cover letter_260211.docx
