# Supplementary material for: Stereo-cell deciphers the spatial and functional heterogeneity of polyploid hepatocytes
Source: Gigascience. 2026 Mar 2;15:giag023. doi: 10.1093/gigascience/giag023 (PMC13100898; doi:10.1093/gigascience/giag023)
Supplement: giag023_Authors_Response_To_Reviewer_Comments_original_submission [file giag023_authors_response_to_reviewer_comments_original_submission.pdf]

Author's Response To Reviewer Comments

Point-by-point response to the reviewers’ comments

We sincerely thank the reviewers for their thoughtful comments and constructive feedback on our work. We particularly appreciate their valuable suggestions regarding the technical details of the SCIPI platform, the depth of data analysis, and the exploration of biological mechanisms. These comments have been instrumental in helping us further improve the quality of our manuscript and enhance the rigor and scientific value of the study. In response, we have carefully addressed each point raised by the reviewers through detailed explanations, additional experiments, and deeper analyses.

Reviewers' comments

Reviewer #1: In this study, the authors developed a specific Sterreo-Cell-based technique to analyze cell ploidy and transcriptomics at the same time. The so-called SCIPI platform owns possible advantages in comparison to other conventional techniques and warrants further application in delineating physiological and pathological condistions. I have a few minor comments.

We greatly appreciate the multiple suggestions you provided for this manuscript. We have revised various problematic or insufficient parts in light of your comments, which has significantly improved the quality of the article. Below are the specific revisions we have made; please kindly review them again.

1. The capture efficiency in this chip was not specifically presented. The results showed different proportion for each ploidy status. Is this comparable to the real situation in mouse liver? I s here any capture bias for specific hepatocyte ploidy populations?

Our reply: This is a crucial question concerning potential capture bias of ploidy subtypes by the SCIPI technology. To address this, we performed fluorescence-activated cell sorting (FACS) based on DAPI fluorescence intensity (DNA content). The sorted populations consisted of 15.5% diploid (2c), 59.6% tetraploid (4c), 22.7% octoploid (8c), and 2.1% hexadecaploid (16c) hepatocytes (see below Rebuttal Fig. 1A). The corresponding FACS results have been added to the revised manuscript.

In parallel, the proportions of hepatocytes captured by the Stereo-cell-based SCIPI technique were 11.4% diploid, 66.2% tetraploid, 21.2% octoploid, and 1.8% hexadecaploid (see below Rebuttal Fig. 1B). The ploidy distribution obtained by SCIPI shows high concordance with that determined by FACS. Furthermore, no significant difference was observed between the ploidy proportions identified by SCIPI and those derived from liver tissue section analysis (see below Rebuttal Fig. 1C). Taken together, we conclude that the differences in subtype proportions between the SCIPI and FACS methods fall within an acceptable range of technical variation. Therefore, the SCIPI technique does not exhibit systematic capture bias against specific hepatocyte ploidy subtypes.

Rebuttal Fig. 1
(A) Proportions of the hepatocyte ploidy subpopulations as identified by SCIPI. (B) Flow cytometric DNA content analysis (DAPI staining) of hepatocytes, showing diploid (2c, 15.5%), tetraploid (4c, 59.6%), octoploid (8c, 22.7%) and hexadecaploid (16c, 2.1%) cells. (C) Hepatocyte ploidy fractions (diploid vs. polyploid) from SCIPI (red) and liver section analysis (green). Two-sided unpaired Student’ s t-test; ns, no significance.

2. As shown by the authors, the mean genes detected per cell in the developed platform was fairly adequate, but remained substantially lower than the FACS-based single-cell RNA profiling strategy. This may represent a limitation for the current pipeline, particularly for analysis of more complicatied disease contexts. This should be furtherdiscussed.

Our reply: Regarding gene detection sensitivity, we acknowledge that our current SCIPI approach yields a

lower gene detection rate compared to FACS-based single-cell RNA profiling strategies, which we anticipate will be significantly improved through future technological upgrades. However, compared to FACS-sorted scRNA-seq (~350 cells; DOI: 10.1016/j.devcel.2023.07.006), our method captured a substantially larger number of qualified cells (~7,700) and achieved higher ploidy subtype classification accuracy. This point is now discussed in the revised manuscript.

Furthermore, to demonstrate the functional utility of our data despite the lower per-cell gene count, we successfully integrated our dataset with the published scRNA-seq data of homeostasis adult mouse liver (DOI: 10.1038/s41588-024-01709-7). The integrated analysis shows excellent alignment among the datasets (see below Rebuttal Fig. 2), indicating the data quality remains sufficient for robust downstream omics analyses, including clustering, integration, and biological interpretation.

Rebuttal Fig. 2
(A) UMAP plots of integrated scRNA-seq data showing the zonation annotation. (B) UMAP plots of integrated scRNA-seq data showing the distribution of ploidy subpopulations across mononuclear diploid (2n×1), mononuclear tetraploid (4n×1), binuclear diploid (2n×2), mononuclear octoploid (8n×1), and binuclear tetraploid (4n×2) cells. (C) UMAP plots show hepatocyte ploidy subpopulation distribution from this study dataset.

3. There were a few wirtten errors, i.e. Fig.4C-G Fig.4H should be Fig.5.

Our reply: Thank you for pointing out this error. We have revised the manuscript accordingly and carefully checked for any remaining typographical mistakes elsewhere.

Reviewer #2: This study develops an imaging-based ploidy identification (SCIPI) pipeline using the Stereo-cell spatially resolved single-cell sequencing technology. It successfully achieves, for the first time, the simultaneous acquisition of transcriptomic and ploidy information from the same cell, systematically resolving the spatial distribution and functional heterogeneity of four key hepatocyte polyploid subtypes (2n×1, 2n×2, 4n×1, 4n×2) in mice. The technical design is highly innovative, the data quality is robust, and the work represents a significant advance in the field of hepatic polyploidy. I recommend conditional acceptance pending revisions, which should address the need for additional key validations and more detailed data analysis in certain areas.

We sincerely appreciate your recognition of our manuscript. You have also pointed out several urgent issues that need to be addressed in our research and provided valuable insights for solving them—this has been extremely helpful to us, further enriching and enhancing the significance of our manuscript and making our conclusions more robust. Following your suggestions, we have supplemented several experiments and analytical content, revised errors in the manuscript, and filled in the insufficient parts. Below are the specific revisions; please kindly review them.

1.Weaknesses and suggestions
(1)Technical validation requires sreangthening
Issue: The accuracy of SCIPI's ploidy classification relies on nuclear area measurement and K-means clustering but lacks systematic validation against a gold standard (e.g., DNA content quantification by flow cytometry).
Suggestions:
a.Perform flow cytometric DNA content analysis on a random subset of cells and compare the results with SCIPI classifications (e.g., using a confusion matrix) to establish concordance.

b.Validate the linear relationship between nuclear area and DNA content (e.g., by isolating hepatocyte nuclei and quantifying DAPI fluorescence intensity).

Our reply: Your suggestion is highly critical, as this validation was indeed missing from our previous work. We performed correlation analysis between DAPI fluorescence intensity and the corresponding nuclear area using FACS-sorted ploidy subpopulations, with three biological replicates. The relevant results have been

updated in the manuscript (new Fig. 3H-M, Fig. S1B-G). For the FACS-sorted diploid (2c), tetraploid (4c), and octoploid (8c) hepatocytes, correlation analysis between the DAPI fluorescence intensity and nuclear area of each ploidy subtype showed a strong positive correlation, with a coefficient of 0.82 ($p=2.7\times10^{-5}$). Additionally, the fluorescence intensity of FACS-sorted diploid, tetraploid, and octoploid cells was highly positively correlated with the nuclear area of corresponding cell types identified by SCIPI ($R=0.9$, $p=5.1\times10^{-7}$).

In summary, nuclear area can serve as a reliable proxy for DNA content in ploidy identification. These results further validate the rationale and accuracy of the SCIPI technique for ploidy classification based on nuclear area measurement.

(2) Biological mechanism exploration in superficial
Issue: The study is primarily descriptive. The mechanistic drivers behind the functional differences among polyploid subtypes (e.g., why 4n×2 cells show enhanced metabolic gene expression) are not deeply explored.
Suggestions:
a.Perform pathway enrichment analysis (e.g., on metabolic pathways, cell cycle pathways) on differentially expressed genes to elucidate the molecular basis of functional enhancements in polyploids.

b.Integrate with published scRNA-seq datasets of hepatocytes to investigate if specific polyploid subtypes correspond to distinct functional states (e.g., oxidative metabolism, detoxification).

Our reply: Thank you for your valuable suggestion! We have additionally performed differential expression analysis and functional enrichment analysis based on ploidy groups and distinct karyotype-resolved ploidy subtypes and the relevant results have been updated in the manuscript (new Fig. 5A–G, Fig. S3A–C). These analyses primarily characterize the molecular alterations that occur in distinct polyploid groups compared with diploid hepatocytes.

We observed that tetraploid hepatocytes exhibited fewer upregulated differentially expressed genes (DEGs) compared to octoploid cells. Furthermore, the set of genes upregulated in octoploid cells encompassed those upregulated in tetraploid cells. In contrast, no upregulated DEGs were detected in diploid cells relative to polyploid hepatocytes. Gene Ontology (GO) enrichment analysis of these DEGs revealed significant enrichment in pathways related to cytoplasmic translation, small molecule catabolism, and generation of precursor metabolites and energy, reflecting enhanced metabolic and biosynthetic activity in octoploid cells.

To dissect differences attributable specifically to nuclear number independent of total DNA content, we next compared mononucleated and binucleated polyploid cells. We found that binucleated hepatocytes, both within tetraploid and octoploid populations, displayed a greater number of upregulated DEGs compared to their mononucleated counterparts. Notably, the DEGs upregulated in both mononucleated and binucleated tetraploid cells were largely contained within the octoploid DEG set. GO analysis of these DEGs showed that both tetraploid and octoploid hepatocytes shared enrichment in pathways including oxidative phosphorylation, ATP synthesis coupled electron transport, and aerobic respiration. Additionally, octoploid cells were uniquely enriched in pathways related to small molecule metabolism, organic acid metabolism, and fatty acid metabolism.

We integrated the sequencing data obtained by SCIPI with a published single-cell transcriptomic dataset of homeostasis adult mouse hepatocytes (DOI: 10.1038/s41588-024-01709-7). While the SCIPI data integrated well with the published dataset, we observed that hepatocytes of different ploidy subtypes exhibited an irregularly intermixed and seemingly random distribution, regardless of integration (see Rebuttal Fig. 2B-C). Consequently, it was not feasible to assign the ploidy information from SCIPI to the cells in the published scRNA-seq dataset using methods based on clustering or spatial proximity projection. Therefore, regarding hepatocyte ploidy subtypes, we were unable to perform a combined analysis with the published data to conduct a more in-depth exploration of functional pathway differences across ploidies.

(3)Data statistics and presentation need optimization

Issue:Statistical significance is not indicated in figures 3D and 4C-E; The heatmap in figure 5B lacks functional annotations for gene clusters, making interpretation difficult.

Our reply: Thank you for your valuable comments. We have now updated the relevant figures accordingly. In Fig. 3D we performed significance tests and added error bars along with overlaid scatter plots of the data. In Fig. 4C–E, we have also included significance testing. These statistical methods are described in the corresponding figure legends of the manuscript. Furthermore, we have comprehensively revised the heatmap originally shown in Fig. 5B to improve its clarity and readability, now presented as new Fig. 5C. Additionally, relevant gene enrichment analysis has been included as new Fig. 5D.

(4)Discussion of technical limitations should be deepened
Issue: Potential RNA diffusion between adjacent cells is mentioned but its specific impact on the polyploid transcriptome data is not assessed.

Our reply: Since the Stereo-cell system employs an open reaction setup, RNA diffusion between neighboring cells is theoretically possible. As reported in the published Stereo-cell methodology paper, the peak proportion of heterologous RNA in individual cells after co-seeding human-mouse mixed cells onto the Stereo-cell chip is ~0.027, a value that does not compromise data accuracy (DOI: 10.1126/science.adr0475). In our study, we addressed this potential issue through multiple experimental and computational strategies. Experimentally, we optimized the cell loading density on the chip to achieve a moderate cell density, which spatially minimizes diffusion effects. During data quality control, we implemented a multi-modal imaging-based cell contour QC pipeline to filter out cells with abnormal nucleus-to-cytoplasm ratios or positional overlap. We further employed a deep learning model (StarDist) to enhance segmentation accuracy for multinucleated and polyploid cells. Subsequently, spatial transcript clustering analysis was performed to verify that transcriptomic signals align with cellular morphology.

Through this integrated approach—encompassing density optimization, morphological filtering, multi-modal QC, high-precision computational segmentation, and spatial transcript verification—we obtained high-quality Stereo-cell data for hepatocytes. Moreover, our hepatocyte data show excellent integration with published single-cell liver datasets. In conclusion, we believe the potential impact of diffusion on our final data is minimal.

(5)Lack of details crucial for reproducibility
Issue: Key parameters like cell adhesion efficiency and RNA capture efficiency are not quantified.
Suggestions:
a.Quantify and report hepatocyte adhesion rates to the chip and RNA capture efficiency (perhaps compared to standard scRNA-seq protocols).

Our reply: We acknowledge the initial omission in our data reporting and have now supplemented the relevant statistical analyses. As demonstrated in the published Stereo-cell technology article, direct comparison on PBMC samples showed that Stereo-cell outperformed 10x Chromium in terms of RNA capture efficiency and specificity (DOI: 10.1126/science.adr0475). In our study, for the two independent biological replicates (hep56_H8 and hep56_M1), a high inter-replicate correlation was observed (R^2=0.87), confirming the technical robustness and reproducibility of the Stereo-cell platform (manuscript, Fig. S1A). Regarding the experimental details, we loaded 5,573 cells onto the Stereo-chip for sample hep56_H8, with 3,164 cells retained for analysis after quality control, resulting in an effective cell recovery rate of ~57%. For sample hep56_M1, 2,148 cells were loaded, and 1,724 cells passed QC, yielding an effective recovery rate of ~80%. The overall cell capture performance was satisfactory, though some variation existed between replicates. This variation may be attributed to our stringent quality control criteria, which were implemented to ensure high-quality, low-contamination single-cell data.

The RNA capture metrics for the two biological replicates were as follows: for hep56_H8, the mean gene count was 444 and the mean UMI count was 2,136; for hep56_M1, the mean gene count was 688 and the mean UMI count was 3,785 (see below Rebuttal Fig. 3A). The calculated RNA capture efficiency ranged from 4.7% to 5.2%, with a mitochondrial read proportion between 1.2% and 3.5%. These values are moderately lower than the median gene count (~1,000, Liang et al., see below Rebuttal Fig. 3B) reported for P56 hepatocytes captured using the 10x Genomics Chromium platform (DOI:

10.1016/j.devcel.2022.01.004).

Rebuttal Fig. 3
(A) Quality control of hepatocyte Stereo-cell sequencing dataset from our study. (B) The number of genes detected in hepatocytes at each chosen time point from Liang et al.'s .

b.Ensure the GitHub code repository includes example test data and detailed setup/execution guidelines.

Our reply: We have now updated the documentation on GitHub, where a detailed tutorial has been added to the GitHub introduction page and workable example data has been uploaded. You may access the updated content at the following link: https://github.com/JeffLuo9/Hep_Ploidy_protocol/.

2.Specific revision recommendations
(1)Methods: Clarify the normalization method for nuclear areas used in K-means clustering (e.g., is it corrected for cell size variation?).

Our reply: For K-means clustering, the nuclear area was standardized solely by converting from pixel units to square micrometers (μm²). Beyond this conversion, no additional correction or normalization of nuclear or cellular area was performed. The detailed procedure has been added to the Results section of the manuscript on lines 126-131.

(2)Results: Include a direct transcriptomic comparison between 4n×1 and 2n×2 cells to explore the specific influence of nuclear number independent of total ploidy.

Our reply: We further investigated the functional differences associated with nuclear number, independent of total DNA content. The corresponding results have been added to the Results section of the manuscript on lines 259-268 (new Fig. 5E-I, Fig. S3A-D).

(3)Discussion: Compare the advantages and limitations of the SCIPI approach for polyploid analysis against other recent spatial omics technologies (e.g., MERFISH).

Our reply: The advantages and limitations of the SCIPI technique compared to other methods, such as FACS-sorted scRNA seq and MERFISH, have been added to the Discussion section of the manuscript on lines 310-326.

(4)Figures: For Figure 4B, consider adding quantitative statistics for spatial distribution (e.g., density curves along the CV-PV axis).

Our reply: We have updated Fig. 4B by adding density curves to visualize the CV-PV axis scores across different ploidy subtypes (manuscript, new Fig. 4B–C).

Editorial Manager®

Cookie Preference Center

We use cookies which are necessary to make our site work. We may also use additional cookies to analyze, improve and personalize our content and your digital experience. For more information, see our Cookie Policy.

You may choose not to allow some types of cookies. However, blocking some types may impact your experience of our site and the services we are able to offer. See the different category headings below to find out more or change your settings.

Allow all

Manage Consent Preferences

Strictly Necessary Cookies

Always active

These cookies are necessary for the website to function and cannot be switched off in our systems. They are usually only set in response to actions made by you which amount to a request for services, such as setting your privacy preferences, logging in or filling in forms. You can set your browser to block or alert you about these cookies, but some parts of the site will not then work.

Functional Cookies

These cookies enable the website to provide enhanced functionality and personalisation. They may be set by us or by third party providers whose services we have added to our pages. If you do not allow these cookies then some or all of these services may not function properly.

Performance Cookies

These cookies allow us to count visits and traffic sources so we can measure and improve the performance of our site. They help us to know which pages are the most and least popular and see how visitors move around the site.

Targeting Cookies

These cookies may be set through our site by our advertising partners. They may be used by those companies to build a profile of your interests and show you relevant adverts on other sites. If you do not allow these cookies, you will experience less targeted advertising.

Cookie List

Clear

Apply Cancel
Consent Leg.Interest

Confirm my choices

Power
