# Supplementary material for: Stereo-cell deciphers the spatial and functional heterogeneity of polyploid hepatocytes
Source: Gigascience. 2026 Mar 2;15:giag023. doi: 10.1093/gigascience/giag023 (PMC13100898; doi:10.1093/gigascience/giag023)
Supplement: giag023_Reviewer_1_Report_original_submission — Reviewer 1 -- 12/30/2025 [file giag023_reviewer_1_report_original_submission.pdf]

Reviewer Report

Title: Stereo-cell deciphers the spatial and functional heterogeneity of polyploid hepatocytes

Version: Original Submission **Date:** 12/30/2025

Reviewer name: Reviewer 1

Reviewer Comments to Author:

In this study, the authors developed a specific Sterreo-Cell-based technique to analyze cell ploidy and transcriptomics at the same time. The so-called SCIPI platform owns possible advantages in comparison to other conventional techniques and warrants further application in delineating physiological and pathological condistions. I have a few minor comments.1. The capture efficiency in this chip was not specifically presented. The results showed different proportion for each ploidy status. Is this comparable to the real situation in mouse liver? Is there any capture bias for specific hepatocyte ploidy populations?2. As shown by the authors, the mean genes detected per cell in the developed platform was fairly adequate, but remained substantially lower than the FACS-based single-cell RNA profiling strategy. This may represent a limitation for the current pipeline , particularly for analysis of more complicatied disease contexts. This should be further discussed.3. There were a few wirtten errors, i.e. Fig.4C-G Fig.4H should be Fig.5.

Level of Interest

Please indicate how interesting you found the manuscript: Choose an item.

Quality of Written English

Please indicate the quality of language in the manuscript: Choose an item.

Declaration of Competing Interests

Please complete a declaration of competing interests, considering the following questions:

- Have you in the past five years received reimbursements, fees, funding, or salary from an organisation that may in any way gain or lose financially from the publication of this manuscript, either now or in the future?
- Do you hold any stocks or shares in an organisation that may in any way gain or lose financially from the publication of this manuscript, either now or in the future?
- Do you hold or are you currently applying for any patents relating to the content of the manuscript?
- Have you received reimbursements, fees, funding, or salary from an organization that holds or has applied for patents relating to the content of the manuscript?
- Do you have any other financial competing interests?

- Do you have any non-financial competing interests in relation to this paper?

If you can answer no to all of the above, write 'I declare that I have no competing interests' below. If your reply is yes to any, please give details below.

I declare that I have no competing interests.

I agree to the open peer review policy of the journal. I understand that my name will be included on my report to the authors and, if the manuscript is accepted for publication, my named report including any attachments I upload will be posted on the website along with the authors' responses. I agree for my report to be made available under an Open Access Creative Commons CC-BY license (http://creativecommons.org/licenses/by/4.0/). I understand that any comments which I do not wish to be included in my named report can be included as confidential comments to the editors, which will not be published.

Choose an item.

To further support our reviewers, we have joined with Publons, where you can gain additional credit to further highlight your hard work (see: https://publons.com/journal/530/gigascience). On publication of this paper, your review will be automatically added to Publons, you can then choose whether or not to claim your Publons credit. I understand this statement.

Yes Choose an item.
