# Supplementary material for: Stereo-cell deciphers the spatial and functional heterogeneity of polyploid hepatocytes
Source: Gigascience. 2026 Mar 2;15:giag023. doi: 10.1093/gigascience/giag023 (PMC13100898; doi:10.1093/gigascience/giag023)
Supplement: giag023_Reviewer_2_Report_original_submission — Reviewer 2 -- 1/4/2026 [file giag023_reviewer_2_report_original_submission.pdf]

**Reviewer Report**

**Title: Stereo-cell deciphers the spatial and functional heterogeneity of polyploid hepatocytes**

**Version: Original Submission** **Date:** 1/4/2026

**Reviewer name: Reviewer 2**

**Reviewer Comments to Author:**

This study develops an imaging-based ploidy identification (SCIPI) pipeline using the Stereo-cell spatially resolved single-cell sequencing technology. It successfully achieves, for the first time, the simultaneous acquisition of transcriptomic and ploidy information from the same cell, systematically resolving the spatial distribution and functional heterogeneity of four key hepatocyte polyploid subtypes (2nÃ—1, 2nÃ—2, 4nÃ—1, 4nÃ—2) in mice. The technical design is highly innovative, the data quality is robust, and the work represents a significant advance in the field of hepatic polyploidy. I recommend conditional acceptance pending revisions, which should address the need for additional key validations and more detailed data analysis in certain areas.1.Weaknesses and suggestions(1)Technical validation requires sreangtheningIssue: The accuracy of SCIPI's ploidy classification relies on nuclear area measurement and K-means clustering but lacks systematic validation against a gold standard (e.g., DNA content quantification by flow cytometry).Suggestions:a.Perform flow cytometric DNA content analysis on a random subset of cells and compare the results with SCIPI classifications (e.g., using a confusion matrix) to establish concordance.b.Validate the linear relationship between nuclear area and DNA content (e.g., by isolating hepatocyte nuclei and quantifying DAPI fluorescence intensity).(2)Biological mechanism exploration in superficialIssue: The study is primarily descriptive. The mechanistic drivers behind the functional differences among polyploid subtypes (e.g., why 4nÃ—2 cells show enhanced metabolic gene expression) are not deeply explored.Suggestions:a.Perform pathway enrichment analysis (e.g., on metabolic pathways, cell cycle pathways) on differentially expressed genes to elucidate the molecular basis of functional enhancements in polyploids.b.Integrate with published scRNA-seq datasets of hepatocytes to investigate if specific polyploid subtypes correspond to distinct functional states (e.g., oxidative metabolism, detoxification).(3)Data statistics and presentation need optimizationIssue:Statistical significance is not indicated in figures 3D and 4C-E; The heatmap in figure 5B lacks functional annotations for gene clusters, making interpretation difficult.(4)Discussion of technical limitations should be deepenedIssue: Potential RNA diffusion between adjacent cells is mentioned but its specific impact on the polyploid transcriptome data is not assessed.(5)Lack of details crucial for reproducibilityIssue: Key parameters like cell adhesion efficiency and RNA capture efficiency are not quantified.Suggestions:a.Quantify and report hepatocyte adhesion rates to the chip and RNA capture efficiency (perhaps compared to standard scRNA-seq protocols).b.Ensure the GitHub code repository includes example test data and detailed setup/execution guidelines.2.Specific revision recommendations(1)Methods: Clarify the normalization method for nuclear areas used in K-means clustering (e.g., is it corrected for cell size variation?).(2)Results: Include a direct transcriptomic comparison between 4nÃ—1 and 2nÃ—2 cells to explore the specific influence of nuclear number independent of total ploidy.(3)Discussion: Compare the advantages and limitations of the SCIPI

approach for polyploid analysis against other recent spatial omics technologies (e.g., MERFISH).(4)Figures: For Figure 4B, consider adding quantitative statistics for spatial distribution (e.g., density curves along the CV-PV axis).

**Level of Interest**

Please indicate how interesting you found the manuscript: Choose an item.

**Quality of Written English**

Please indicate the quality of language in the manuscript: Choose an item.

**Declaration of Competing Interests**

Please complete a declaration of competing interests, considering the following questions:

- Have you in the past five years received reimbursements, fees, funding, or salary from an organisation that may in any way gain or lose financially from the publication of this manuscript, either now or in the future?
- Do you hold any stocks or shares in an organisation that may in any way gain or lose financially from the publication of this manuscript, either now or in the future?
- Do you hold or are you currently applying for any patents relating to the content of the manuscript?
- Have you received reimbursements, fees, funding, or salary from an organization that holds or has applied for patents relating to the content of the manuscript?
- Do you have any other financial competing interests?
- Do you have any non-financial competing interests in relation to this paper?

If you can answer no to all of the above, write 'I declare that I have no competing interests' below. If your reply is yes to any, please give details below.

I declare that I have no competing interests

I agree to the open peer review policy of the journal. I understand that my name will be included on my report to the authors and, if the manuscript is accepted for publication, my named report including any attachments I upload will be posted on the website along with the authors' responses. I agree for my report to be made available under an Open Access Creative Commons CC-BY license (http://creativecommons.org/licenses/by/4.0/). I understand that any comments which I do not wish to be included in my named report can be included as confidential comments to the editors, which will not be published.

Choose an item.

To further support our reviewers, we have joined with Publons, where you can gain additional credit to further highlight your hard work (see: https://publons.com/journal/530/gigascience). On publication of this paper, your review will be automatically added to Publons, you can then choose whether or not to claim your Publons credit. I understand this statement.

Yes Choose an item.
